# The Far Side of Carboranes: Anticancer Active Monocations and Ambiently Stable Dications

**DOI:** 10.1002/anie.202521595

**Published:** 2026-04-02

**Authors:** Vlastimil Němec, Josef Holub, Maksim A. Samsonov, Zdeňka Růžičková, Josef Cvačka, Ján Vančo, Zdeněk Trávníček, Jan Belza, Zdeněk Dvořák, Jan Vrána, Aleš Růžička

**Affiliations:** ^1^ Department of General and Inorganic Chemistry Faculty of Chemical Technology University of Pardubice Pardubice Czech Republic; ^2^ Institute of Inorganic Chemistry Czech Academy of Sciences Řež Czech Republic; ^3^ Institute of Organic Chemistry and Biochemistry Czech Academy of Sciences Flemingovo náměstí Praha Czech Republic; ^4^ Regional Centre of Advanced Technologies and Materials Czech Advanced Technology and Research Institute Palacký University Olomouc Czech Republic; ^5^ Department of Cell Biology and Genetics Faculty of Sciences Palacký University Olomouc Czech Republic

**Keywords:** carborane cluster, cation, cellular effects, in vitro cytotoxicity, proton‐coupled electron transfer

## Abstract

Polyhedral carboranes are highly biologically stable, non‐toxic clusters. Whereas they are typically encountered in anionic or neutral forms, positively charged species have only recently been discovered. The in vitro antiproliferative effects of selected carboranes were assessed using a panel of human cancer cell lines, and off‐target toxicity was evaluated at normal cell lines. The results demonstrated significant anticancer activity and a favorable resistance factor (RF ≈ 1) for monocationic carborane ***o*‐2a**, surpassing the effects of *doxorubicin* and *cisplatin*. In pursuit of even more efficient substrates, the first dicationic polyhedral boranes were synthesized. These water‐stable dications exhibit a reversible *closo‐*/*nido‐* cage opening, triggered either by a strong base (DMAP) or by a combination of triethylamine and molecular hydrogen, and reversed upon the addition of acid. The former transformation proceeds without a redox change, while the latter involves a H_2_/2H^+^ conversion in a proton‐coupled electron process.

## Graphical abstract

This work describes the synthesis of bench‐stable dicationic carboranes. Their enhanced electrophilicity is documented by opening the clusters with weak nucleophiles or dihydrogen. The surprising stability of positively charged carboranes in water enabled a study of their in vitro antiproliferative activity, which surpassed the effects of doxorubicin and cisplatin.

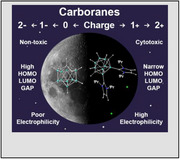

## 1 Introduction

Polyhedral boranes and heteroboranes occupy a unique position at the intersection of inorganic and organic chemistry, showing reactivity reminiscent of both main‐group hydrides and aromatic systems [1, 2, 3]. Due to the delocalization of electron density across the entire cluster, neutral and anionic (hetero)boranes possess exceptional thermal stability among molecular compounds. The small electronegativity difference between boron and hydrogen renders the B–H bond generally nonpolar yet capable of exhibiting either protic or hydridic character depending on the molecular structure.

With hundreds of structural motifs reported to date, boranes and heteroboranes have been extensively studied for applications in many areas such as energy storage and conversion, materials science, nuclear‐waste remediation, and medicine [4, 5, 6, 7, 8, 9]. Their medicinal potential is particularly promising owing to their low toxicity and high biological stability. Thanks to their steric and electronic properties, these clusters can act as phenyl mimetics, replacing organic moieties in various drugs, anticancer agents, and pharmacophores [10, 11, 12, 13, 14, 15, 16, 17, 18, 19]. Their inherent hydrophobicity makes them ideal candidates for therapeutic and diagnostic labelling as well as radiotherapies such as BNCT [20, 21, 22, 23, 24, 25], offering a significantly higher boron‐atom content than non‐cluster compounds.

Although hydrophobicity is often a desirable property, polyhedral boranes can be readily polarized through reduction to anionic species or functionalized with polar organic substituents [26]. Conversely, oxidation to cationic species—which could dramatically alter borane properties such as lipophilic‐cell‐membrane permeability, acid stability, and aqueous solubility—has remained elusive until recently. Although many borane or heteroborane clusters occur as cationic components in molecules, the positive charge is typically localized on exoskeletal groups containing ammonium, phosphonium, or sulfonium moieties [27, 28, 29].

**SCHEME 1:**
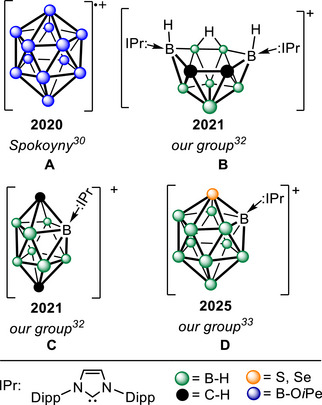
An overview of published cationic boranes and heteroboranes. The *closo*‐radical A is persubstituted with alkoxy groups, whereas the thermally robust heteroboranes are stabilized by bulky N‐heterocyclic carbenes and adopt *pseudonido‐* (B) or *closo‐* (C and D) configurations. Dipp = 2,6‐di*iso*propylphenyl.

The first cationic borane was synthesized via stepwise oxidation of *closo*‐[B_12_(O*i*Pe)_12_]^2−^ to the radical cation *closo*‐[B_12_(O*i*Pe)_12_]•^+^ (Scheme 1A) [30, 31]. The persubstitution of B–H moieties with bulky alkoxy groups proved insufficient for stability, because the radical decomposes above –30°C. The first thermally robust cationic heteroboranes were prepared from 10‐vertex carboranes using complexes with bulky N‐heterocyclic carbenes, which—despite being neutral—exhibited sufficient basicity to accept a proton of H–Cl, yielding cationic carboranes in *pseudonido‐* (Scheme 1B) or *closo‐*configurations (Scheme 1C) [32]. The presence of a positive charge within the cluster was confirmed both spectroscopically and computationally. Recently, we have demonstrated that this strategy can be extended to other heteroboranes, including chalcogenaboranes (Scheme 1D) [33]. These compounds represent unique examples not only within borane‐cluster chemistry but also in group‐13 chemistry in general, as recently reviewed [34].

Given the importance of radiotherapy in contemporary cancer treatment, we focused on boron cluster scaffolds, which intrinsically provide the highest attainable boron content. To date, BNCT agents bearing boranes and heteroboranes have relied predominantly on anionic compounds, including derivatives of [B_12_H_12_]^2–^ and [Co(C_2_B_9_H_11_)_2_]^–^, which exhibit limited nucleophilicity despite their formal negative charge, or on neutral 12‐vertex carboranes, mainly employed as biocompatible appendages or as aryl surrogates in established therapeutic scaffolds [10, 11, 12, 13, 14, 15, 16, 17, 18, 19]. The lack of accessible cationic counterparts has effectively precluded exploration of charge‐inverted architectures.

Herein, we exploit hydrolytically robust polyhedral cationic carboranes to interrogate the biological consequences of introducing a positive charge into the cluster framework. In parallel, we systematically evaluate the role of the counterion and targeted structural modifications in modulating both the chemical behaviour and biological performance of these species.

## 2 Results and Discussion

### 2.1 *o*‐2a Metathesis

**SCHEME 2:**
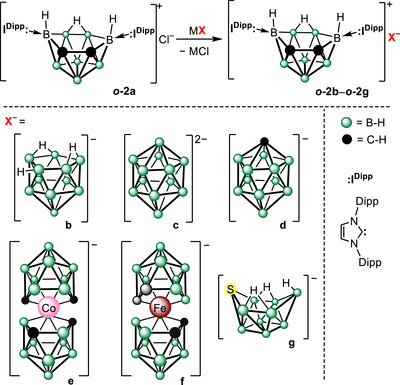
The reactivity of the carborane ***o*‐2a** with anionic polyhedral boranes and heteroboranes. The reactions proceed via simple metathesis, eliminating insoluble chlorides (MCl = NaCl, CsCl, and Et_3_NHCl). Dipp = 2,6‐di*iso*propylphenyl.

To this end, we investigated the chloride‐anion exchange of ***o*‐2a** with various polyhedral boranes and heteroboranes, assessing the stability of two oppositely polarized boranes within a single compound and seeking to increase further the boron‐atom count per compound (Scheme 2). A diverse set of anionic counterparts was selected to represent different structural types of heteroboranes (*closo*‐, *nido*‐, and *arachno*‐) as well as various heteroatoms and charges. The compounds ***o*‐2b**–***o*‐2g** were synthesized via simple metathesis, involving the elimination of insoluble salts (NaCl, CsCl, Et_3_NHCl). To the best of our knowledge, these are the first reported examples of mixed ionic pairs of polyhedral boranes. The NMR spectra of ***o*‐2b**–***o*‐2g** showed no notable differences compared to those of the individual ions. The molecular structure of ***o*‐2e** was confirmed by x‐ray diffraction analysis (Figures S122–S124 also show structures of ***o*‐2c** and ***o*‐2d**, which contained considerable amount of solvent molecules, for more information see the Supporting Information), which revealed no significant intermolecular interactions between the positively and negatively charged clusters—likely due to the delocalization of both positive and negative charges in the respective ions. Unfortunately, the compounds ***o*‐2b**–***o*‐2g** exhibited poor solubility in water (<< 1 mg/mL) and gradual decomposition in solution, leading us to focus biological‐activity testing solely on the compound ***o*‐2a**.

### 2.2 The Evaluation of In Vitro Anticancer Activity

#### 2.2.1 In Vitro Antiproliferative Activity on a Panel of Human Cancer Lines and Normal Cells

The in vitro cytotoxicity of ***o*‐2a** and Cs[Co(1,7‐C_2_B_9_H_11_)_2_] (precursor of ***o*‐2a**, labelled as ***pre‐o*‐2e**) was assessed in seven human cancer cell lines, with ***o*‐2a** further tested in three additional lines. The results (Tables 1 and S15) showed that the monocationic ***o*‐2a** displayed pronounced antiproliferative activity across all cancer cell lines, with IC_50_ values of 1.3–14.0 µM, generally surpassing reference drugs *doxorubicin* and *cisplatin* as well as other compounds tested in this work (see below). Selectivity was evaluated using normal MRC5 and HaCaT cells, with the selectivity index (SI) defined as the ratio of IC_50_ in non‐cancerous to cancer cells. The most favorable SI values (SI≈2) were obtained for A2780 and A2780R. Importantly, ***o*‐2a** exhibited a resistance factor (RF≈1), calculated as the IC_50_ ratio in A2780R/A2780 cells, which is superior to *cisplatin* (RF≈3), indicating promising activity against resistant cancer cell lines.

**TABLE 1:** The results of antiproliferative‐activity testing using the MTT method (24‐h incubation).

	Antiproliferative activity against human cancer and normal cell lines (IC_50_ ± SD)								
Compound	A2780	A2780R	PC3	A549	MCF7	HOS	HT‐29	MRC5	HaCaT
***o*‐2a**	1.4 ± 0.3	1.3 ± 0.4	2.4 ± 0.1	5.0 ± 3.7	3.2 ± 0.1	2.5 ± 0.1	3.4 ± 0.5	2.7 ± 0.4	2.8 ± 0.5
*Doxorubicin*	2.1 ± 1.1	3.6 ± 0.2	>20	>20	3.7 ± 3.4	9.1 ± 3.9	>20	>20	n.t.
*Cisplatin*	17.2 ± 0.9	>50	>50	>50	33.0 ± 4.1	33.2 ± 4.4	>50	>50	>50

*Note*: The IC_50_ values (µM) were determined for the selected compounds and reference drugs (doxorubicin and cisplatin) on human cancer and normal cell lines. The values are presented as the means ± standard deviation (SD). n.t. = not tested. The complete table of cytotoxicity data is given in Table S15.

Ovarian carcinoma, A2780; cisplatin‐resistant ovarian carcinoma, A2780R; prostate carcinoma, PC3; lung adenocarcinoma, A549; breast adenocarcinoma, MCF7; osteosarcoma, HOS; colorectal adenocarcinoma, HT‐29; lung fibroblast, MRC5; aneuploid immortal keratinocyte, HaCaT. ***o‐2a*** was also evaluated on the PANC1, CaCo2 and HeLa cancer cell lines, with IC_50_ values of 3.3 ± 0.2 µM, 13.9 ± 0.4 µM, and 2.7 ± 0.3 µM, respectively.

Based on the outcomes from antiproliferative activity tests using a 24‐h incubation, we aimed to examine how incubation time affects the antiproliferative properties of ***o*‐2a**. Consequently, the time‐dependent antiproliferative activity (with incubation periods of 24, 48, and 72 h) was assessed using the A2780 cell line (see Table S16). These experiments showed a significant decrease in IC_50_ values for ***o*‐2a**, reaching 1.6, 0.5, and 0.4 µM after 24, 48, and 72 h of incubation, respectively. The time‐dependent cytotoxicity of *doxorubicin* and *cisplatin* displayed a typical decline in IC_50_ values over time.

#### 2.2.2 Cellular Effects of Selected Complexes Studied on Ovarian A2780 Cancer Cells

**FIGURE 1:**
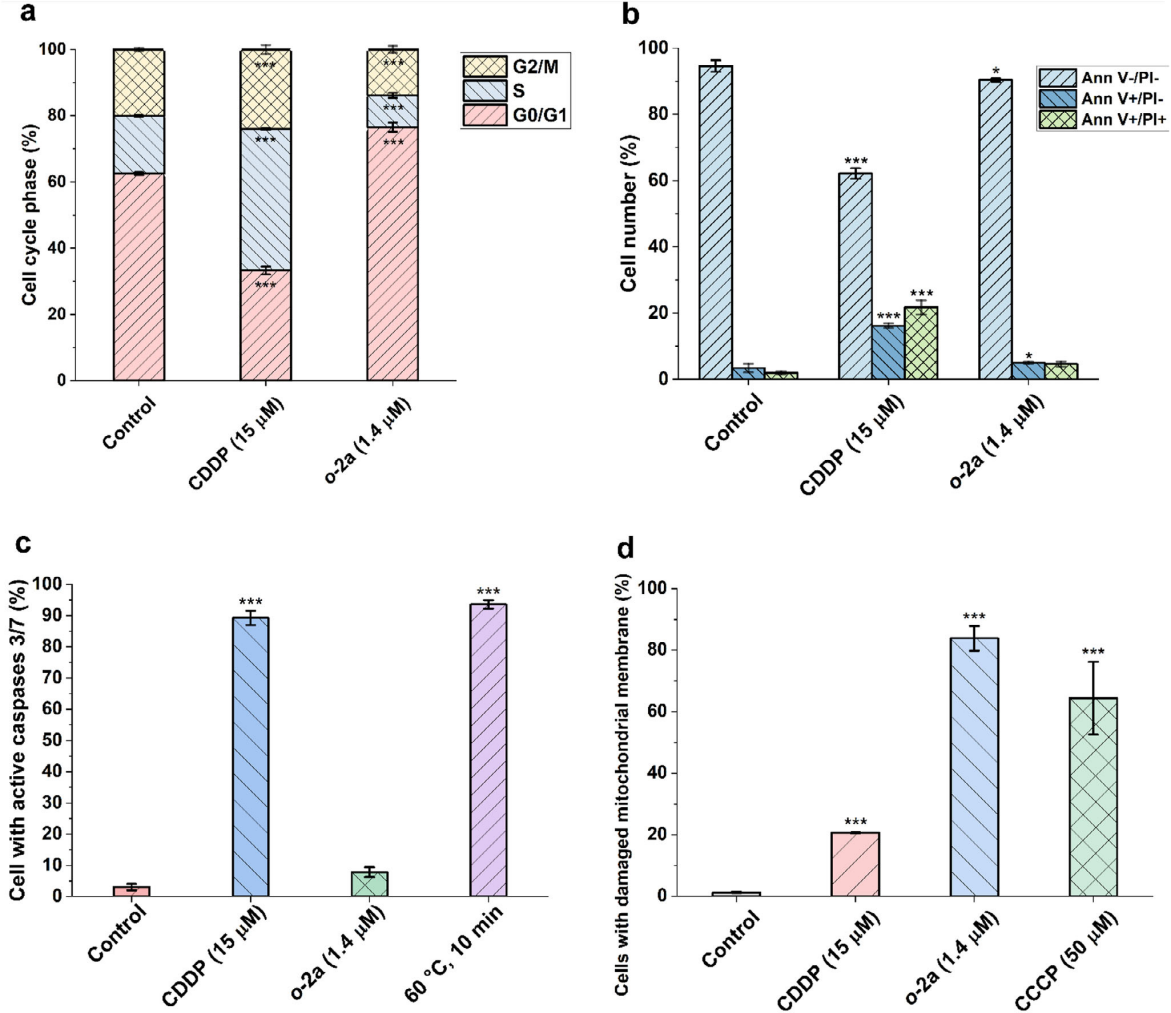
Cellular effects of the tested compounds in A2780 cells. (a) The effects of the tested compounds on the cell cycle. (b) The induction of apoptosis/necrosis in the Annexin V/PI assay. (c) The activation of executioner caspases Casp 3/7. (d) The effect on the mitochondrial‐membrane potential in A2780 cells after 24‐h incubation using half‐cytotoxic concentrations (based on MTT‐assay results). Significance levels: *p<0.05, **p<0.01, ***p<0.005. *Note*: The complete data related to cellular effects are shown in Figure S132.

The effect of the tested compounds on the cell cycle of A2780 cells after 24‐h incubation is shown in Figure 1a. The monocationic ***o*‐2a** potently and significantly arrested A2780 cells in the G0/G1 phase (resting phase) and markedly decreased the cell populations in both the synthetic (S) and G2/M phases of the cell cycle, in contrast to *cisplatin*, which arrested most of the cells in the S phase. These results indicate a completely different mechanism of action for ***o*‐2a** than for *cisplatin*. Following the cell‐cycle analysis, a series of assays describing the ability of the compounds to induce cell‐death processes was performed. Based on the results of the Annexin V/propidium iodide assay (Ann V/PI), the antiproliferative effect of ***o*‐2a** cannot be ascribed to the induction of programmed cell death in A2780 cells (see Figure 1b). C*isplatin* caused significant damage to A2780 cells and initiated apoptosis in more than 30% of all cells.

These findings were further supported by the detection of the active executioner caspases Casp 3/7 in A2780 cells (see Figure 1c). Overall, these results imply that the antiproliferative effects of the studied carboranes may be related to the modulation of the metabolic activity of the target A2780 cells. To reinforce this hypothesis, we investigated the impact of ***o*‐2a** on mitochondrial‐membrane potential (see Figure 1d) and the induction of autophagy (for more details, see Figure S131). Autophagy is typically triggered by environmental stressors that negatively impact cellular metabolism. Nevertheless, ***o*‐2a** showed no significant effects on the activation of autophagy in A2780 cells after 24 h of incubation. In contrast, as shown in Figure 1d, the most potent antiproliferative agent, ***o*‐2a**, caused notable mitochondrial membrane depolarization, exceeding the effects of the reference platinum‐based chemotherapeutic *cisplatin* and the positive control, carbonyl cyanide 3‐chlorophenylhydrazone (CCCP), used at 50 µM. This unexpected, organelle‐targeted activity of ***o*‐2a** clearly disrupts the energetic metabolism of A2780 ovarian cancer cells and might represent a key mechanism behind its antiproliferative effect.

The loss of mitochondrial‐membrane potential can be associated with the structural deterioration of the outer mitochondrial membrane, leading to uncontrolled permeation and the disruption of the proton gradient normally maintained across this membrane. These structural changes can be triggered by the direct damaging effects of reactive oxygen species (ROS), generated either as byproducts of mitochondrial metabolism and/or through depletion of endogenous intracellular antioxidant agents such as glutathione during oxidative stress. Nevertheless, ROS/superoxide‐production analysis in A2780 cells (see Figure S133) revealed either no or a slight antioxidant response from the tested compound.

### 2.3 Modification of the Tested Carborane ***o*‐2a**

**SCHEME 3:**
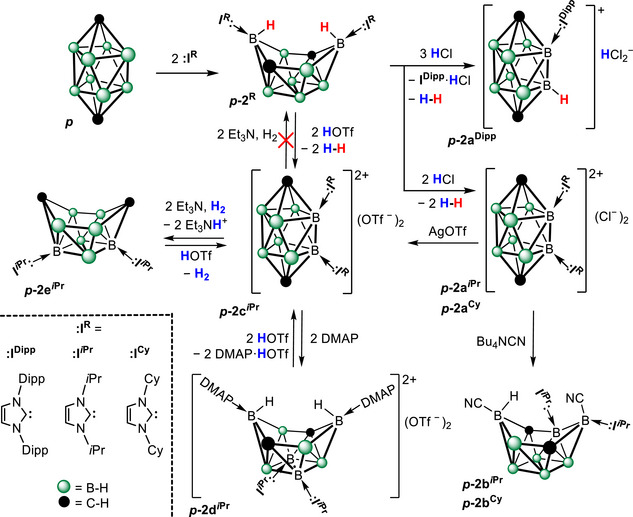
The synthesis and reactivity of dicationic carboranes. The reaction of 1,10‐C_2_B_8_H_10_ (*p*) with the N‐heterocyclic carbene **:I^Dipp^** followed by treatment with hydrogen chloride, yields the monocationic ***p*‐2a^Dipp^** (previous work) [32], whereas **:I*^i^* ^Pr^** and **:I^Cy^** afford the dicationic species ***p*‐2a*^i^* ^Pr^** and ***p*‐2a^Cy^**. The dicationic carboranes ***p*‐2a*^i^* ^Pr^** and ***p*‐2c*^i^* ^Pr^** react with Bu_4_NCN, DMAP, or triethylamine/H_2_, resulting in the opening of the carborane cage irreversibly (***p*‐2b*^i^* ^Pr^**) or reversibly (***p*‐2d*^i^* ^Pr^** and ***p*‐2e*^i^* ^Pr^**), which can be reversed by the addition of an acid. Dipp = 2,6‐di*iso*propylphenyl; Cy = cyclohexyl.

The observed cytotoxicity effectively precludes any application of ***o*‐2a** in radiotherapy and necessitates structural modification of both the cluster core and the supporting carbene ligands. The most plausible origin of this cytotoxicity is insufficient stability under intracellular conditions. According to Wade's rules, ***o*‐2a** adopts an *arachno‐*type framework, which is the least stable toward hydrolysis in the *closo‐* > *nido‐* > *arachno‐* series, despite exhibiting reasonable stability in aqueous media [1, 32]. In order to increase the stability of the positively charged carborane, we turned our attention to *closo‐*carboranes. A single representative of this group (Scheme 1C) was prepared by reacting the carbene–carborane complex ***p*‐2^Dipp^** (Scheme 3) with hydrogen chloride, eliminating dihydrogen and imidazolium in the process. Although the reaction was nearly quantitative (based on the NMR spectra of the reaction mixture), the isolated yield was low due to the challenging separation from **I^Dipp^** ·HCl. Moreover, ***p*‐2a^Dipp^** exhibited lower solubility in water than ***o*‐2a**. To reduce hydrophobicity, we thus employed N‐heterocyclic carbenes (**:I*^i^* ^Pr^** and **:I^Cy^**, Scheme 3) bearing smaller organic substituents. The reaction of *p*‐C_2_B_8_H_10_ (***p***) with these carbenes yielded the expected carboranes ***p*‐2*^i^* ^Pr^** and ***p*‐2^Cy^** (Scheme 3), with significantly shortened reaction times from days to hours. This was attributable to the lower steric hindrance of **:I^Cy^** and **:I*^i^* ^Pr^**, because the reaction proceeds exclusively in a 1:2 ratio. However, the subsequent treatment of ***p*‐2*^i^* ^Pr^** and ***p*‐2^Cy^** with hydrogen chloride did not follow the same pathway as their bulkier analogue. Surprisingly, instead of eliminating one molecule of dihydrogen and imidazolium chloride, two molecules of dihydrogen were released, yielding the positively charged species ***p*‐2a*^i^* ^Pr^** and ***p*‐2a^Cy^** compensated by two chloride anions. Atomic charges in ***p*‐2a*^i^* ^Pr^** and ***p*‐2a^Cy^** were calculated using AIM analysis (Table S9), which revealed that the positive charge is predominantly localized within the carborane cage (1.399 and 1.398 e), while the carbenes are only slightly polarized (0.119–0.166 e). This contrasts with the previously reported clusters from the cationic group 13, where the positive charge is delocalized onto the donor bases, compensating for the cationic center [34]; for example, AIM analysis of tetracationic [{M(dmpe)}_4_]^4+^ (M = Ga, In; dmpe = 1,2‐bis(dimethylphosphino)ethane) has estimated charges of only 0.28 (In) and 0.3 (Ga) e per metal atom [35]. A detailed analysis of individual skeletal atoms in ***p*‐2a*^i^* ^Pr^** and ***p*‐2a^Cy^** shows that the positive charge is evenly distributed across the entire cluster, with no boron atom more charged than the others (see ESI for details). This is further supported by the ^11^B NMR spectra. Whereas the parent carborane ***p*** displays a single signal (−13 ppm), coordination of two carbene molecules lowers the symmetry, dramatically altering the electron distribution and resulting in a broad range of chemical shifts (between −44.5 and −6.6 ppm), which is typical for the *arachno*‐shaped ten vertex clusters [36] and is nearly identical with the spectrum of ***p*‐2^Dipp^** [32]. In contrast, cage closure and formal oxidation to the dication restores the uniform electron distribution, with all boron signals appearing in a narrow range similar to the parent ***p*** (between −8 and −12 ppm). A similar situation was found for the skeletal carbon atoms, as their ^13^C NMR shifts in ***p*** (103.3 ppm), and the dications (106.9 and 107.5 ppm) are strongly downfield‐shifted compared to ***p*‐2*^i^* ^Pr^** and ***p*‐2^Cy^** (28.5 and 28.6 ppm) due to the antipodal effect [37]. These trends have also been observed previously for both monocationic carboranes and chalcogenaboranes [32, 33]. It is also consistent with the fact that these compounds retain their skeletal–electron‐pair (SEP) count as ***p***. According to the Wade–Mingos rules [38, 39, 40, 41, 42], the number of SEPs in ***p*‐2a*^i^* ^Pr^** and ***p*‐2a^Cy^** corresponds to a *closo‐*arrangement, because the formal reduction by carbenes and subsequent oxidation connected via proton‐coupled electron transfer (the protons are formally reduced to dihydrogen molecule) do not alter the SEP count within the cluster, as previously described for chalcogenaboranes [33]. The carboranes ***p*‐2a*^i^* ^Pr^** and ***p*‐2a^Cy^** represent unique examples among group‐13 dications. The delocalization of charge throughout the cluster significantly enhances their stability, allowing them to be handled without an inert atmosphere and to be readily dissolved in water and methanol without decomposition for at least 1 month. In contrast, other group‐13 dicationic compounds are typically sensitive to moisture and oxygen and often require stabilization by weakly coordinating anions [34, 43].

**FIGURE 2:**
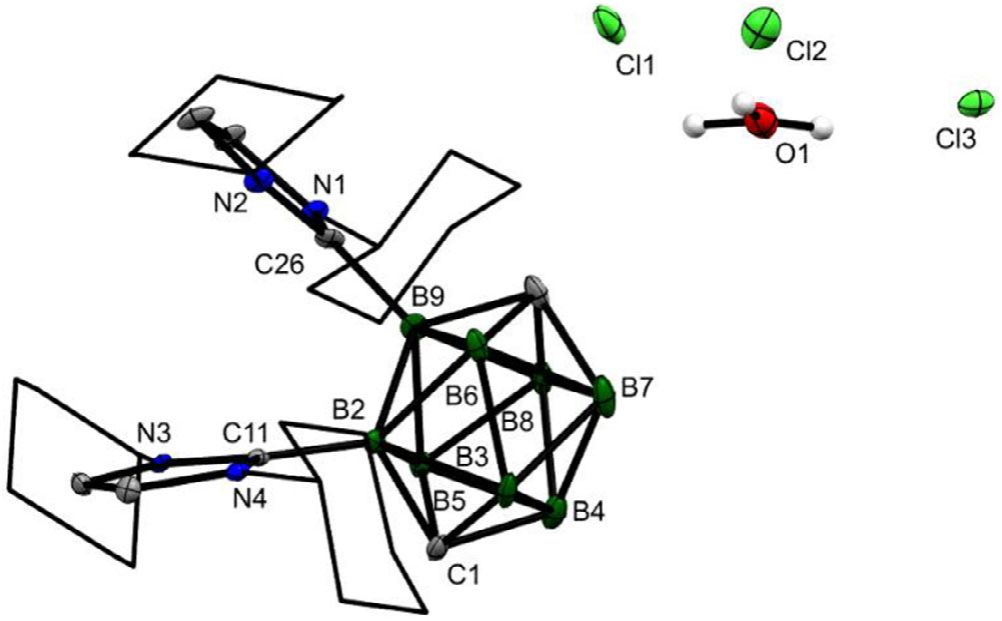
The molecular structure of ***p*‐2a^Cy^**. The ORTEP diagrams are shown at a 40% probability level; the Cy groups are displayed as wireframes for clarity. Solvent molecules have been omitted. Selected interatomic distances [Å]: B–B: 1.796(13)–1.879(12), C1–B: 1.594(13)–1.612(11), C10–B: 1.591(13)–1.612(11), B2–C11: 1.580(11), B9–C26: 1.594(11), Cl1–O1: 2.884(6), Cl2–O1: 2.891(6), Cl3–O1: 2.905(6).

The molecular structures of ***p*‐2a*^i^* ^Pr^** and ***p*‐2a^Cy^** were confirmed by x‐ray diffraction analysis, revealing a *closo*‐arrangement for both dications, with B–B and B–C bond lengths falling within narrow ranges (Figures 2 and S126). Crystallization of ***p*‐2a^Cy^** from undried dichloromethane resulted in the formation of a rare complex counteranion, [(H_3_O)Cl_3_]^2−^. Its formation is most likely a consequence of the absence of direct interactions between chloride ions and the dication, because the delocalized positive charge within the cluster is efficiently shielded by hydride atoms and carbene ligands. To the best of our knowledge, this arrangement has been published previously three times [44, 45, 46], as usually [H_3_OCl_2_]^−^ or its dimer [H_6_O_2_Cl_4_]^2−^ are produced [47]. The chloride atoms are linked with the pyramidalized oxonium cation via hydrogen bonding with separations (2.026–2.166) closer to the sum of covalent radii rather than van der Waals (*Σ*_vdW_ = 3.02; *Σ*_vdW_ = 1.31), which coincides with the earlier reports. Interestingly, it is impossible to dissolve the dications in dried aprotic solvents such as dichloromethane or acetonitrile; however, the addition of water inflicts a partial solubility, apparently due to the solvation of the chloride anions. On the contrary, the insolubility was practically used to wash ***p*‐2a*^i^* ^Pr^** and ***p*‐2a^Cy^** from impurities and enabled smooth multi‐gram scale synthesis.

To shed more light on the formation of dicationic species, the mechanisms of the reactions of ***p*‐2*^i^* ^Pr^** and ***p*‐2^Dipp^** with hydrogen chloride have been investigated computationally (Figure S129) at the B3LYP/def2‐TZVP level of theory using the PCM solvation model (tetrahydrofuran). The SMD model was also tested and yielded similar relative energies (Table S11), with no notable differences in the structures of intermediates and transition states. The first step, which is identical for both reactions, involves coordination of a hydrogen chloride molecule followed by proton transfer to a bridging position (B1–H1–B2, INT‐1). Subsequent elimination of a dihydrogen molecule (TS‐2) leads to the formation of a monocationic intermediate (INT‐2). From this point, the mechanism diverges depending on the steric properties of the carbene. In the case of bulky **:I^Dipp^** (Dipp groups, red path), the most thermodynamically favorable route involves cage closure to ***p*‐2a^Dipp^**, accompanied by the elimination of the carbene, which likely reacts with another equivalent of hydrogen chloride to form an imidazolium salt. Notably, this transition state (TS3′) corresponds to a *Z*‐rearrangement [48] previously described for ten‐vertex boranes and carboranes. In contrast, the less sterically hindered carbenes **:I*^i^* ^Pr^** allow the introduction of a second hydrogen–chloride molecule (INT‐3) and the elimination of dihydrogen (TS‐4) prior to the cage closure. The resulting open dicationic form (TS‐5) is immediately reclosed to ***p*‐2a*^i^* ^Pr^**, consistent with the Z‐rearrangement. To assess the energetic feasibility of alternative products theoretically, we modeled hypothetical mono(di)cationic compounds *p*‐2a^Dipp^* and *p*‐2a*^i^* ^Pr^* with mixed carbene substituents (see, Figure S130). The calculated Gibbs free energies (Δ*G*r) of these species (6.93 and 2.29 kcal/mol) suggest that their formation is energetically disfavored with respect to ***p*‐2a^Dipp^** and ***p*‐2a*^i^* ^Pr^** (−37.40 and −35.64 kcal/mol) and thus unlikely.

**FIGURE 3:**
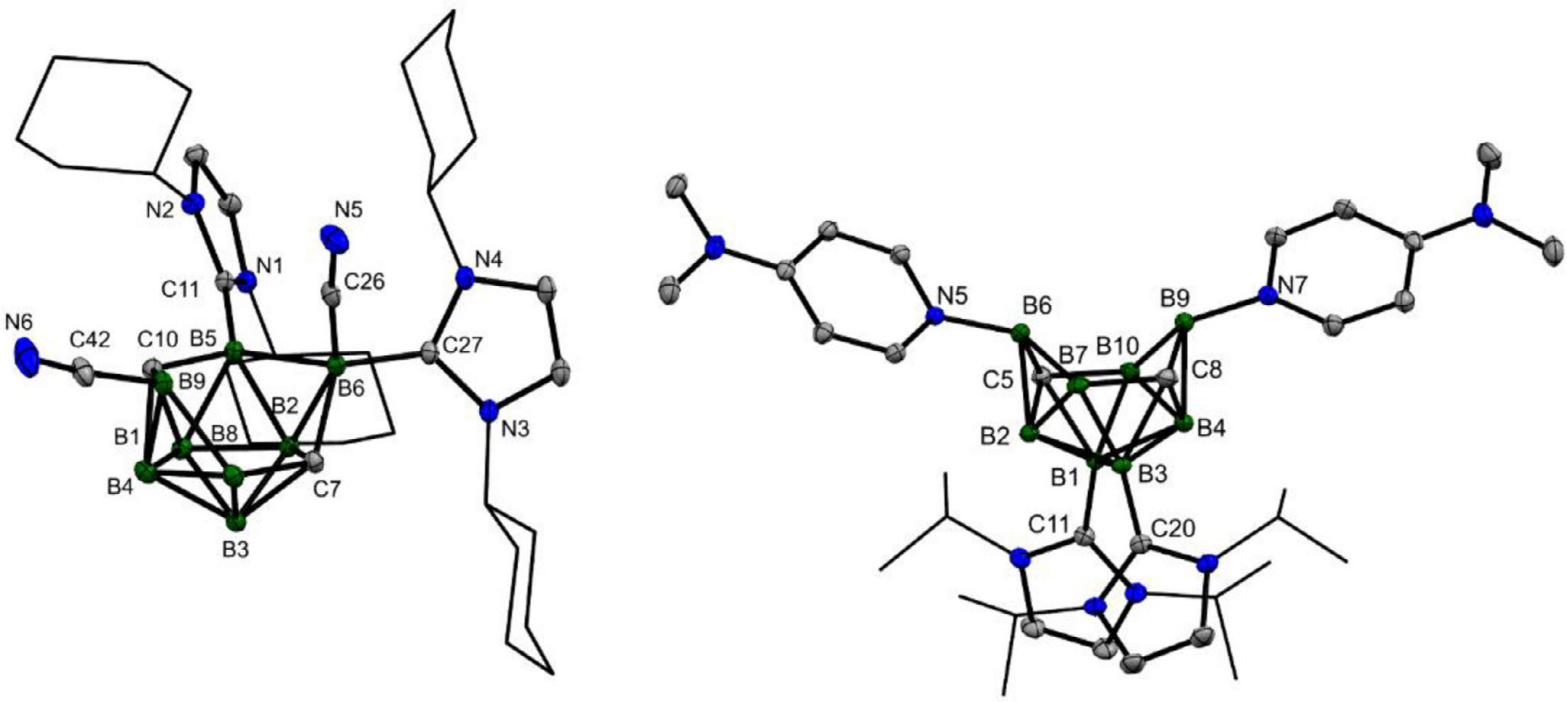
The molecular structures of ***p*‐2b^Cy^** and ***p*‐2d*^i^* ^Pr^**. The ORTEP diagrams are shown at a 40% probability level; the *i*Pr and Cy groups are displayed as wireframes for clarity. Solvent molecules and anions have been omitted. Selected interatomic distances [Å]: ***p*‐2b^Cy^** B9–C42: 1.569(5), B6–C26: 1.579(5), C42–N6: 1.143(6), C26–N5: 1.151(5), B5–C11: 1.569(5), B6–C27: 1.655(5), B8–B9: 1.853(6), B9–C10: 1.635(5), C10–B5: 1.618(5), B5–B6: 1.924(6), B6–C7: 1.690(5), C7–B8: 1.609(5); ***p*‐2d*^i^* ^Pr^** N5–B6: 1.572(2), N7–B9: 1.573(2), C5–B6: 1.758(2), B6–B7: 1.742(2), B7–C8: 1.619(2), C8–B9: 1.711(2), B9–B10: 1.791(2), B10–C5: 1.600(2).

We have evaluated the in vitro antiproliferative effects of the dicationic compounds ***p*‐2a*^i^* ^Pr^** and ***p*‐2a^Cy^**, which, in contrast to the monocationic ***o*‐2a**, were inactive up to their solubility limits, except for ***p*‐2a^Cy^**, which showed moderate activity (IC_50_ = 11.6 µM) in A2780 and A2780R cells. This might be attributed to their higher stability provided by the *closo*‐arrangement, as we have expected. The low cytotoxicity makes them more suitable for potential radiotherapy studies compared to ***o*‐2a**. The stabilizing effect of the *closo‐*arrangement outweighs the increased polarity of the doubly charged carboranes, resulting in higher stability both in aqueous solution and in cellular environments. Notably, the steric bulk of the carbene substituents appears to have a lower impact on the overall stability of the cationic clusters. Although carboranes ***p*‐2a*^i^* ^Pr^** and ***p*‐2a^Cy^** have the same number of skeletal electron pairs (SEPs) as compound ***p***, their electrophilicity should be enhanced, as demonstrated by the reactivity of selenaboranes with weak bases such as sodium iodide and 2,6‐di*iso*propylaniline [33]. It was thus a logical step to test the similar reactivity of dicationic species. Even in protic solvents (water and methanol), no reaction with halogens (Bu_4_NX, X = Br, I) was observed, prompting us to explore more reactive pseudohalogens (Bu_4_NX, X = NCS, CN, N_3_). While thiocyanate left ***p*‐2a*^i^* ^Pr^** and ***p*‐2a^Cy^** intact, two cyanide anions opened the cluster to form neutral ***p*‐2b*^i^* ^Pr^** and ***p*‐2b^Cy^** (Scheme 3). This reaction proceeded even at low temperatures, in contrast to conventional cyanation of borane clusters, which typically requires elevated temperatures and/or transition‐metal catalysts [49, 50, 51, 52]. A plausible mechanism involves initial coordination of a cyanide to atom B5, followed by cage opening (Figure S127). A second cyanide then coordinates to one of the carbene‐bearing boron atoms, yielding ***p*‐2b*^i^* ^Pr^** or ***p*‐2b^Cy^**. Unfortunately, ***p*‐2b^Cy^** could not be isolated from the reaction mixture due to a similar solubility with the reaction by‐products; however, its molecular structure has been confirmed by x‐ray diffraction analysis (Figure 3).

Similarly, we tested the reaction with azide. The ^11^B NMR spectrum of the reaction mixture revealed multiple species with patterns resembling ***p*‐2b*^i^* ^Pr^**, suggesting the formation of several isomers. This is not surprising considering the number of reactive boron atoms exposed upon cage opening.

In the early 2000s, main‐group compounds in unusual oxidation states gained attention as transition‐metal mimetics due to their ability to activate small molecules such as hydrogen and carbon dioxide [53]. Comparable chemistry has not been described for polyhedral boranes, except in cases involving reactivity at exoskeletal functional groups [54]. Since the reaction of ***p*‐2*^i^* ^Pr^** with hydrogen chloride to ***p*‐2a*^i^* ^Pr^** released elemental hydrogen, we hypothesized that base‐induced rehydrogenation could establish a cycle of dihydrogen activation.

To test this, dication ***p*‐2a*^i^* ^Pr^** was treated with triethylamine and DMAP, but both reactions led to decomposition of the compound in both water and methanol, with visible gas evolution. To avoid protic solvents, we replaced chloride with triflate via metathesis using AgOTf, yielding ***p*‐2c*^i^* ^Pr^**, which is soluble in acetonitrile and can also be prepared directly by treating ***p*‐2*^i^* ^Pr^** with triflic acid. These findings are consistent with the results of the ***o*‐2a** metathesis study, which demonstrated that pairing a sterically demanding cation with a small anion is optimal for stability in protic environments, whereas incorporation of a larger anion reduces lattice energy and thereby enhances solubility in less polar solvents. The subsequent reaction with DMAP produced ***p*‐2d*^i^* ^Pr^**. It strongly resembled the reaction of ***p*** with **:I*^i^* ^Pr^** and resulted in cage opening to a *pseudonido*‐arrangement via the *Z*‐rearrangement (Figure S128), described above. Unlike the reaction with cyanide, this transformation began at atom B4, opposite the carbene‐bearing vertex B2, most likely due to steric factors.

The carborane ***p*‐2d*^i^* ^Pr^** shares structural features with ***p*‐2*^i^* ^Pr^** but remains positively charged due to the loss of two hydrogen atoms. To compare their chemical behavior, we treated ***p*‐2d*^i^* ^Pr^** with hydrogen chloride (triflic acid), but the more polarized B–N bond proved weaker than the B―C(carbene) bond and was readily quenched, generating ***p*‐2a*^i^* ^Pr^** (***p*‐2c*^i^* ^Pr^**). The reaction with triethylamine, a considerably weaker base, did not proceed under strict anhydrous conditions. However, triethylamine must be capable of activating the dication, as decomposition has been observed in protic solvents.

Therefore, we repeated the reaction with triethylamine under a hydrogen atmosphere (1.5 bar), successfully obtaining the hydrogenation product ***p*‐2e*^i^* ^Pr^**, formed via hydrogenation to a *nido‐*shaped cluster, followed by deprotonation. Notably, the same reaction did not proceed without triethylamine, whose presence is essential for proton removal, as demonstrated by the reverse reaction of ***p*‐2e*^i^* ^Pr^** with triflic acid, which smoothly regenerated the dicationic ***p*‐2c*^i^* ^Pr^**. Unfortunately, ***p*‐2e*^i^* ^Pr^** was obtained in low yield due to the over‐hydrogenation of the dicationic ***p*‐2c*^i^* ^Pr^**, as evidenced by the ^11^B NMR spectrum of the reaction mixture, which showed various hydrogenation products, including the Et_3_N–BH_3_ complex.

To the best of our knowledge, the only comparable hydrogenation of a *closo‐*borane is that of B_10_H_10_^2^ **^−^**, which reacts with strong protic acids to form the *nido‐*type complexes 6,9‐L_2_B_10_H_12_ (L = Et_2_S, CH_3_CN) [55]. This reaction is reversible upon base addition, regenerating B_10_H_10_^2−^. The opening and closing patterns of both clusters are similar, as they retain the same SEP count, but the polarity is inverted—from 2+ (***p*‐2a*^i^* ^Pr^**) to 2− (B_10_H_10_^2^ **^−^**) in the *closo‐*compounds‒which is, to the best of our knowledge, the largest polarity difference between two isostructural boranes reported to date. Notably, this process involves a redox couple (H_2_/2H^+^), whereas the decaborate system undergoes simple proton exchange.

We did not observe similar hydrogenation of the monocationic ***p*‐2a^Dipp^** (Scheme 3), likely due to its lower electrophilicity. This is reflected in the HOMO–LUMO‐gap values, which decrease in the order ***p*** > ***p*‐1a^Dipp^** > ***p*‐2a*^i^* ^Pr^** (7.29, 3.31, and 2.22 eV, respectively; see Table S10 for details).

**FIGURE 4:**
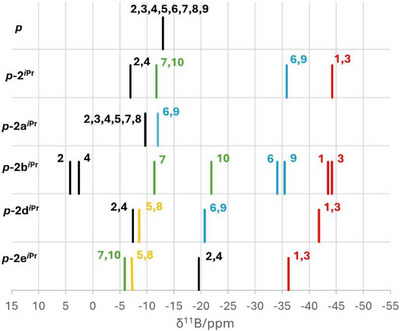
Correlation diagram of ^11^B NMR spectra of the carborane *p*‐C_2_B_8_H_10_ (***p***) and prepared compounds. The *closo‐*carboranes ***p*** and ***p*‐2a*^i^* ^Pr^** exhibit signals around −11 ppm, whereas the *nido*‐ and *arachno*‐ are distributed in a broad range.

The ^11^B NMR spectra of the opened carboranes revealed expected patterns in a broad range of chemical shifts (−4.2–(−44.2) ppm) corresponding to *arachno‐* (***p*‐2b*^i^* ^Pr^** and ***p*‐2d*^i^* ^Pr^**) and *nido‐* (***p*‐2e*^i^* ^Pr^**) type clusters. Differences between the respective cluster arrangements are clearly shown in the correlation diagram (Figure 4, for more information see Supporting Information, Table S1). The shifts of individual positions in the ^11^B spectra vary only with the change of substituents (typically hydrogen–carbene substitution).

## 3 Conclusion

A series of high‐boron‐content ionic carboranes has been synthesized via the ion exchange of various borane and heteroborane clusters. Among them, the cationic carborane ***o*‐2a** exhibited notable antiproliferative activity, outperforming classic anticancer agents such as *doxorubicin* and *cisplatin*. Targeting the applications of the positively charged clusters in BNCT, we started to explore structural modifications of ***o*‐2a**, moving toward a more stable *closo‐*arrangement. The elusive dicationic carboranes ***p*‐2a*^i^* ^Pr^** and ***p*‐2a^Cy^** were prepared through formal reduction of the 10‐vertex carborane ***p*** to *nido‐*type complexes, followed by cage closure via formal oxidation with hydrogen chloride—in the end, without altering the SEP count in the *closo*‐carboranes. Unlike other group‐13 dications, ***p*‐2a*^i^* ^Pr^** and ***p*‐2a^Cy^** retain most of their positive charge within the cluster, as confirmed by theoretical calculations and spectroscopic data. Despite their high charge, both dications are air‐stable and water‐soluble, showing no decomposition after 1 month. Their stability was also reflected in their low antiproliferative activity, which makes them suitable candidates for BNCT studies. The enhanced electrophilicity is demonstrated by their uncatalyzed reaction with cyanide, which proceeds rapidly even at ambient temperature. The dicationic triflate ***p*‐2c*^i^* ^Pr^** undergoes reversible opening with DMAP and recloses upon acid treatment. Interestingly, reaction with triethylamine and molecular hydrogen does not yield the expected precursor ***p*‐2*^i^* ^Pr^** but its isomer ***p*‐2e*^i^* ^Pr^**, which can also be reclosed by acid. This reversible transformation involves a redox couple (H_2_/2H^+^) and suggests potential for dihydrogen activation‒an uncommon feature among polyhedral boranes (excluding exoskeletal reactivity). Such proton‐coupled electron transfer transformations are involved in many challenging reductions ranging from organic substrates to elemental nitrogen [56, 57].

## Conflicts of Interest

The authors declare no conflicts of interest.

## Supporting information




**Supporting File 1**: The Supporting Information contains details on the synthesis, characterization, and crystal data [58] of the compounds prepared and computational details. The authors have cited additional references within the Supporting Information [59, 60, 61, 62, 63, 64, 65, 66, 67, 68, 69, 70, 71, 72].

**Supporting File 2**: anie71935‐sup‐0002‐Data.zip.

## Data Availability

The raw NMR data of this study are openly available in Figshare.com at https://doi.org/10.6084/m9.figshare.30084781.
