# Supplementary material for: The Far Side of Carboranes: Anticancer Active Monocations and Ambiently Stable Dications
Source: Angew Chem Int Ed Engl. 2026 Apr 2;65(20):e21595. doi: 10.1002/anie.202521595 (PMC13159407; doi:10.1002/anie.202521595)
Supplement: Supplementary file 1 — Supporting File 1: The Supporting Information contains details on the synthesis, characterization, and crystal data [58] of the compounds prepared and computational details. The authors have cited additional references within the Supporting Information [59, 60, 61, 62, 63, 64, 65, 66, 67, 68, 69, 70, 71, 72]. [file ANIE-65-e21595-s001.DOCX]

Supporting Information for:

The Far Side of Carboranes: Anticancer Active Monocations and Ambiently Stable Dications

Vlastimil Němec,^a^ Josef Holub,^b^ Maksim A. Samsonov,^a^ Zdeňka Růžičková,^a^ Josef Cvačka,^c^ Ján Vančo,^d^ Zdeněk Trávníček,^d^ Jan Belza,^d^ Zdeněk Dvořák,^e^ Jan Vrána*,^a^ Aleš Růžička*^a^

1. Department of General and Inorganic Chemistry, Faculty of Chemical Technology, University of Pardubice, Studentská 573, 532 10 Pardubice, Czech Republic, email: jan.vrana@upce.cz; ales.ruzicka@upce.cz
2. Institute of Inorganic Chemistry, Czech Academy of Sciences, 250 68 Řež, Czech Republic
3. Institute of Organic Chemistry and Biochemistry, Czech Academy of Sciences, Flemingovo náměstí 542/2, 166 10 Praha 6, Czech Republic
4. Regional Centre of Advanced Technologies and Materials, Czech Advanced Technology and Research Institute, Palacký University, Šlechtitelů 27, CZ-779 00, Olomouc, Czech Republic
5. Department of Cell Biology and Genetics, Faculty of Science, Palacký University, Šlechtitelů 27, CZ-779 00, Olomouc, Czech Republic

**Table of Contents:**

1. Synthesis and Characterization S3

2. Crystallographic Data S76

3. Reactions of ***p*-2a*^i^*^Pr^** with bases S89

4. Theoretical Investigations S90

5. Biological Evaluations *in vitro* S94

6. References S100

**Synthesis and characterization**

**Materials and Methods**

All air and moisture sensitive manipulations were carried out under an argon atmosphere using standard Schlenk tube technique. Solvents were dried using a PureSolv–Innovative Technology equipment under argon gas atmosphere. Deuterated solvents were purchased from Euriso-Top GmbH. Polyhedral borates [Et_3_NH][B_11_H_14_], Na_2_[B_12_H_12_], Ag[CB_11_H_12_] were purchased from Katchem s.r.o.. The starting compounds 1,2-C_2_B_8_H_10_,^[59]^ 1,10-C_2_B_8_H_10_,^[60]^ ***o*-2a**,^[32]^ Cs[2,2´-Co(1,7-C_2_B_9_H_11_)_2_],^[61]^ Cs[3,3´-Fe(1,2-C_2_B_9_H_11_)_2_],^[61]^ Cs[6-SB_9_H_12_],^[62]^ **:I*^i^*^Pr^**,^[63]^ **:I^Cy^**,^[64]^ were prepared according to the published procedures.

Abbreviations used in the manuscript and the supporting information.

## **NMR spectroscopy**

^1^H, ^11^B, and ^13^C{^1^H} NMR spectra were recorded on Bruker Avance I 500 MHz spectrometer or Bruker Ultrashield 400 MHz, using 5 mm tunable broad-band probe. Appropriate chemical shifts in ^1^H and ^13^C{^1^H} NMR spectra were related to the residual signals of the solvent (CD_3_CN: δ(^1^H) = 1.94 ppm, δ(^13^C) = 1.32 ppm; CD_2_Cl_2_ = δ(^1^H) = 5.32 ppm, δ(^13^C) = 54.00 ppm; THF-D_8_: δ(^1^H) = 3.58 ppm, δ(^13^C) = 67.21 ppm; CD_3_OD = δ(^1^H) = 3.31 ppm, δ(^13^C) = 49.00 ppm).

## **sc-XRD**

Full-sets of diffraction data for ***o*-2c** (2487180), ***o*-2d** (2487178), ***o*-2e** (2487177), ***p*-2^Cy^** (2487175)**, *p*-2a*^i^*^Pr^** (2487173), ***p*-2a^Cy^** (2487174), ***p*-2b^Cy^** (2487179) and ***p*-2d^Cy^** (2487176) were collected at 150(2)K with a Bruker D8-Venture diffractometer equipped with Mo (Mo/K_α_ radiation; λ = 0.71073 Å) microfocus X-ray (IµS) sources, Photon CMOS detector and Oxford Cryosystems cooling device was used for data collection. The structures of ***o*‑2d** and ***o*‑2c** seem to be highly problematic because its spherical molecules crystallize to plate-like crystals together with small molecules of solvents, which are highly disordered. Eight disordered molecules of hexane and eight molecules of dichloromethane are present within the unit cell of ***o*-2d**, while 168 water molecules could be localized within the unit cell of ***o*-2c.** These disordered molecules have been masked by PLATON /SQUEZZE program (A. L. Spek, *Acta Cryst. C Struct. Chem.* **2015**, *71*, 9–18.), which resulted in 725 and 132- electrons per unit cells (voids of 736 and 1344 electrons were found theoretically in solvent accessible volumes of 2936 and 4306 Å^3^, see Figure S122). mo_jvr230301N2_p2b(Cy) is highly twinned. Therefore, no conclusions about the structures of compounds ***o*‑2d** and ***o*‑2c** have been drawn from their solid-state structures, and they are included only for illustration.

The frames were integrated with the Bruker SAINT software package using a narrow-frame algorithm. Data were corrected for absorption effects using the Multi-Scan method (SADABS). Obtained data were treated by XT-version 2014/5 and SHELXL-2014/7 software implemented in APEX3 v2016.5-0 or APEX4 v2021.10-0 (Bruker AXS) system.^[65]^

Hydrogen atoms were mostly localized on a difference Fourier map, however, to ensure uniformity of treatment of crystal, all hydrogen were recalculated into idealized positions (riding model) and assigned temperature factors H_iso_(sept) = 1.2 U_eq_ (pivot atom) or of 1.5U_eq_ (methyl). Hydrogen atoms in methyl, benzhydryl C-H moieties and aromatic rings were placed with C-H distances of 0.96, 1.00 and 0.93 Å, while hydrogens in N-H or B-H groups were placed according to the maxima on the Fourier maps and refined freely.

*R*_int_ = ∑⏐*F*_o_^2^ - *F*_o,mean_^2^⏐/∑*F*_o_^2^, S = [∑(*w*(*F*_o_^2^ - *F*_c_^2^)^2^)/(*N*_diffrs_ - *N*_params_)]^½^ for all data, *R*(*F*) = ∑⏐⏐*F*_o_⏐ - ⏐*F*_c_⏐⏐/∑⏐*F*_o_⏐for observed data, *wR*(*F*^2^) = [∑(*w*(*F*_o_^2^ - *F*_c_^2^)^2^)/(∑*w*(*F*_o_^2^)^2^)]^½^ for all data.

Crystallographic data for structural analyses have been deposited with the Cambridge Crystallographic Data Centre, CCDC nos. 2487173-2487180. Copies of this information may be obtained free of charge from The Director, CCDC, 12 Union Road, Cambridge CB2 1EY, UK (fax: +44-1223-336033; e-mail: deposit@ccdc.cam.ac.uk or www: http://www.ccdc.cam.ac.uk).

**Mass Spectrometry**

Mass spectra were acquired on an Orbitrap IQ-X Tribrid mass spectrometer (Thermo Fisher Scientific, Waltham, MA, USA) operated at a resolving power of 500,000. The instrument was equipped with a heated electrospray ionization (HESI) source, set to 275 °C. The spray voltage was maintained at 3.5 kV, and the ion transfer tube temperature was set to 300 °C for both positive and negative ionization modes. Samples were prepared in acetonitrile and introduced into the system via a 10 μL injection loop. The mobile phase consisted of a methanol/water mixture (4:1, v/v) delivered at a flow rate of 100 μL/min. Mass calibration was performed automatically using the instrument's Auto-Ready ion source feature.

**Synthesis of [6,9-I^Dipp^_2_-5,10-C_2_B_8_H_11_][B_11_H_14_] (*o*-2b)**

A solution of [Et_3_NH][B_11_H_14_] (27 mg, 0.12 mmol) in dichloromethane (2 mL) was added to the stirred solution of ***o*-2a** (103 mg, 0.12 mmol) in dichloromethane (2 mL) at room temperature, and the suspension was stirred for one hour. The volatiles were removed in *vacuo,* and the solid was extracted with tetrahydrofuran (5 mL). The volatiles were removed in *vacuo* giving ***o*-2b** as a white powder. Yield 107 mg, 91 %. **Mp. > 320 °C. ^1^H NMR** (25 °C, CD_2_Cl_2_, 500 MHz): *δ* = −4.17 (s broad, 1H, B7*H*B8 bridge), −3.64 (s broad, 2H, B*H*B bridge), 0.60 (s, 2H, BC*H*), 1.09 (d, *^3^J*(^1^H, ^1^H) = 6.83 Hz, 12H, CH(C*H*_3_)_2_), 1.12 (d, *^3^J*(^1^H, ^1^H) = 6.92 Hz, 12H, CH(C*H*_3_)_2_), 1.15 (d, *^3^J*(^1^H, ^1^H) = 6.92 Hz, 12H, CH(C*H*_3_)_2_), 1.19 (d, *^3^J*(^1^H, ^1^H) = 6.92 Hz, 12H, CH(C*H*_3_)_2_), 2.19 (sept, *^3^J*(^1^H, ^1^H) = 6.86 Hz, 4H, C*H*(CH_3_)_2_), 2.39 (sept, *^3^J*(^1^H, ^1^H) = 6.84 Hz, 4H, C*H*(CH_3_)_2_), 7.14 (s, 4H, C*H*=C*H*), 7.27 (d, *^3^J*(^1^H, ^1^H) = 7.64 Hz, 8H, *m*-C_6_*H*_3_), 7.55 (t, *^3^J*(^1^H, ^1^H) = 7.83 Hz, 4H, *p*-C_6_*H*_3_) ppm. **^13^C{^1^H} NMR** (25 °C, CD_2_Cl_2_, 125.76 MHz): *δ* = 22.5, 23.3, 25.8, 26.0 (s, CH(*C*H_3_)_2_), 26.4 (s, B*C*H), 29.4 (s, *C*H(CH_3_)_2_), 124.8 (s, *C*H=*C*H), 124.9, 125.2 (s, *m*-*C*_6_H_3_), 131.9 (s, *p*-*C*_6_H_3_), 132.7 (s, *ipso*-*C*_6_H_3_), 145.6, 146.1 (s, *o*-*C*_6_H_3_), 160.3 (s very broad, N*C*N) ppm. **^11^B NMR** (25 °C, CD_2_Cl_2_, 160.46 MHz) *δ* = −54.4 (d, ^1^*J*(^1^H,^11^B) = 149.96 Hz, 1B, B3), −31.3 (d, ^1^*J*(^1^H,^11^B) = 119.96 Hz, 2B, B6,9), −22.2 (s broad, 1B, B1), −16.8 (d, ^1^*J*(^1^H,^11^B) = 137.62 Hz, 5B, B3´−11´), −15.8 (d, ^1^*J*(^1^H,^11^B) = 136.18 Hz, 5B, B2´−6´), −14.2 (d, ^1^*J*(^1^H,^11^B) = 139.86 Hz, 1B, B1´), −7.0 (s broad, 2B, B2,4), −4.3 (s broad, 2B, B7,8) ppm.

**Spectroscopic characterization of *o-*2b**


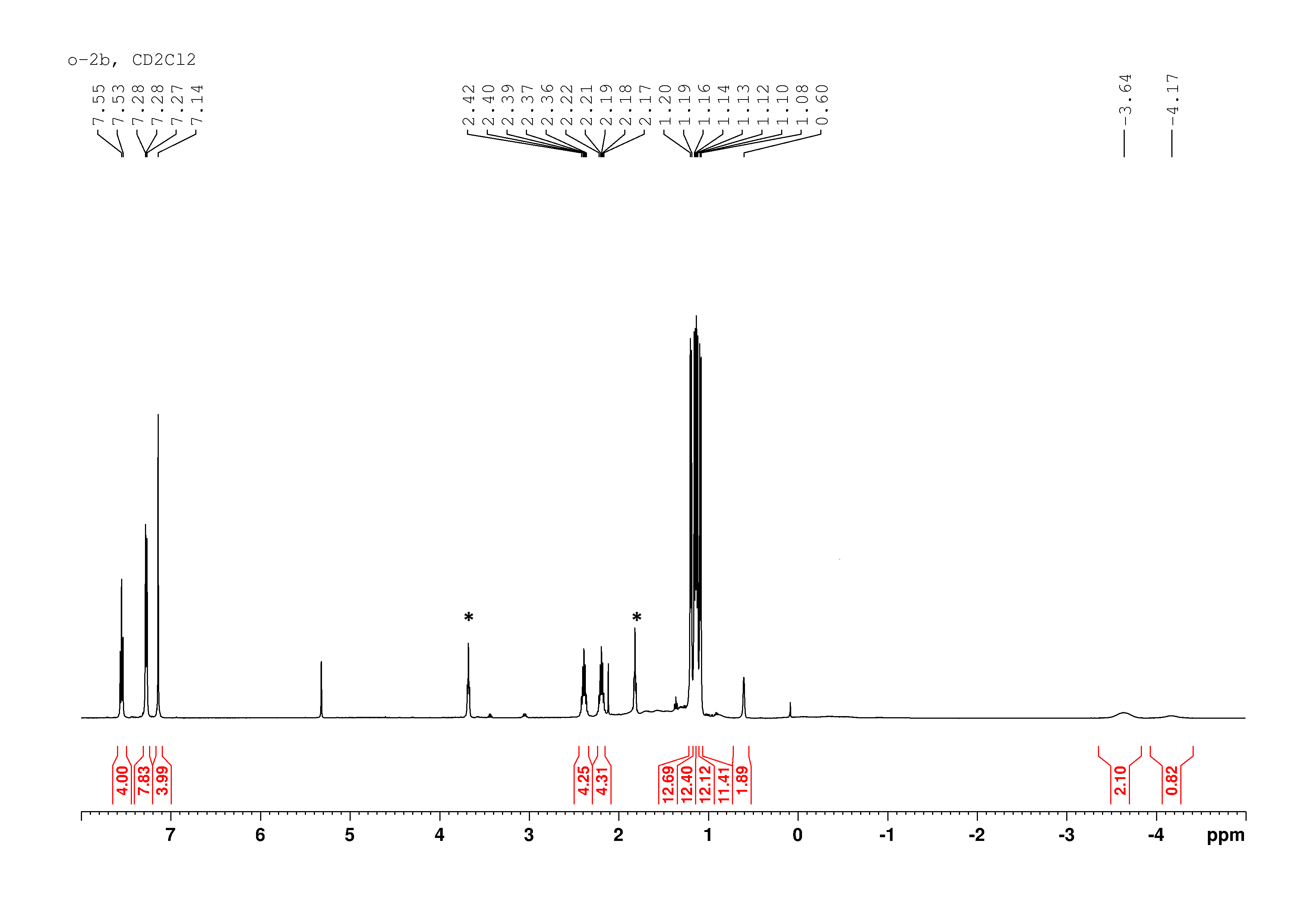


**Figure S1**. ^1^H NMR spectrum of **o-2b**. Residual tetrahydrofuran is marked by *.


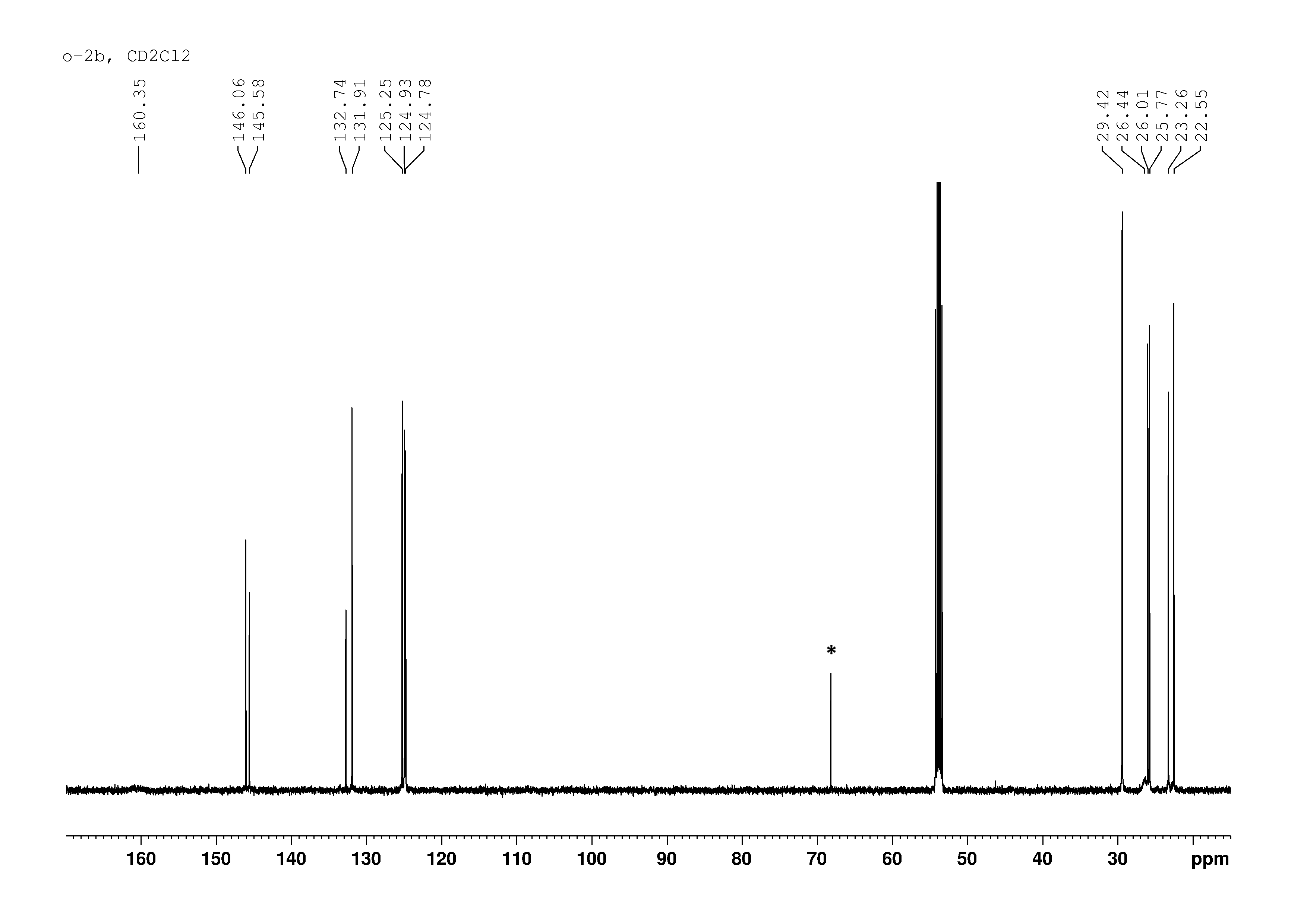


**Figure S2**. ^13^C{^1^H} NMR spectrum of **o-2b**. Residual tetrahydrofuran is marked by *.


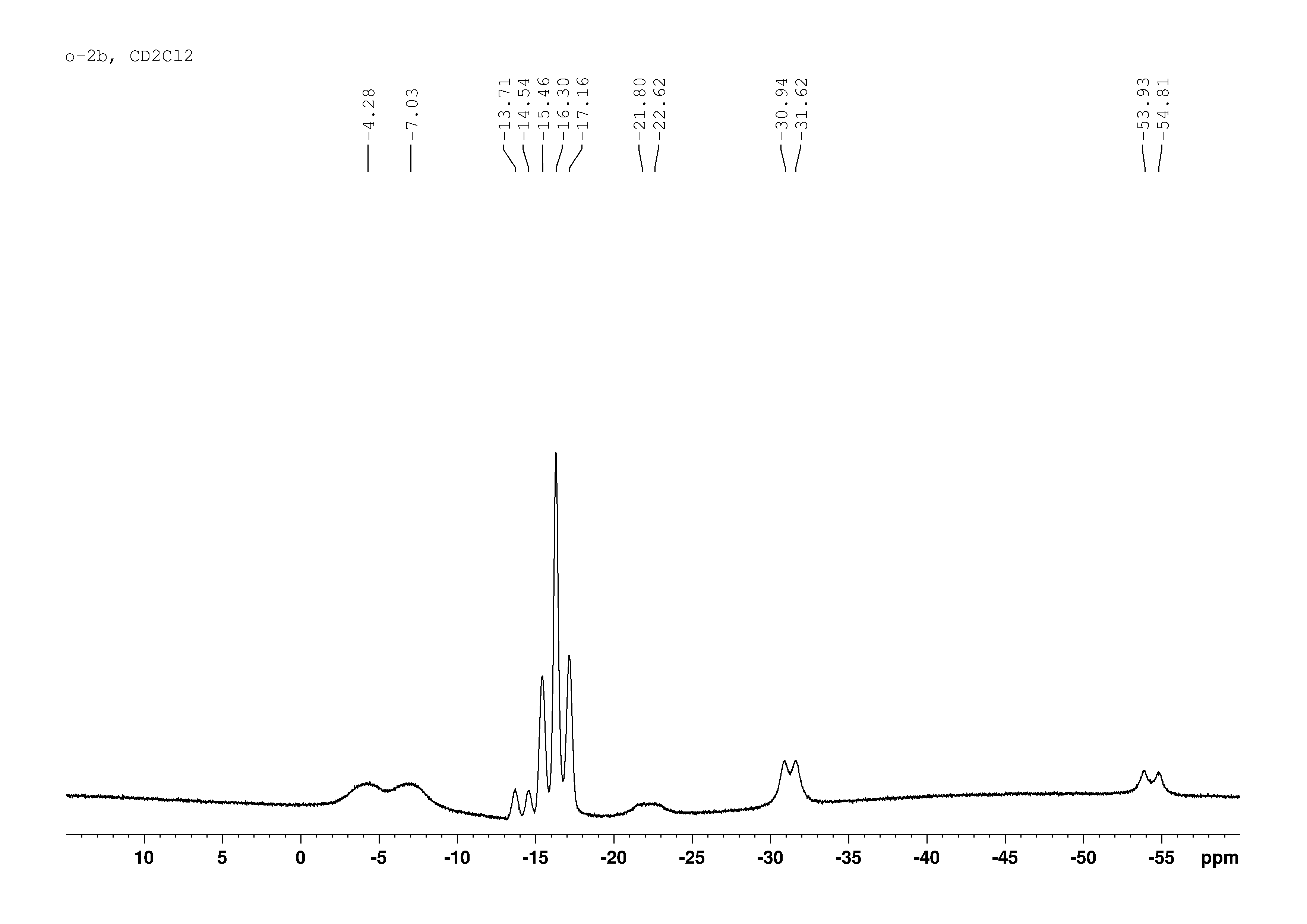


**Figure S3**. ^11^B NMR spectrum of **o-2b**.


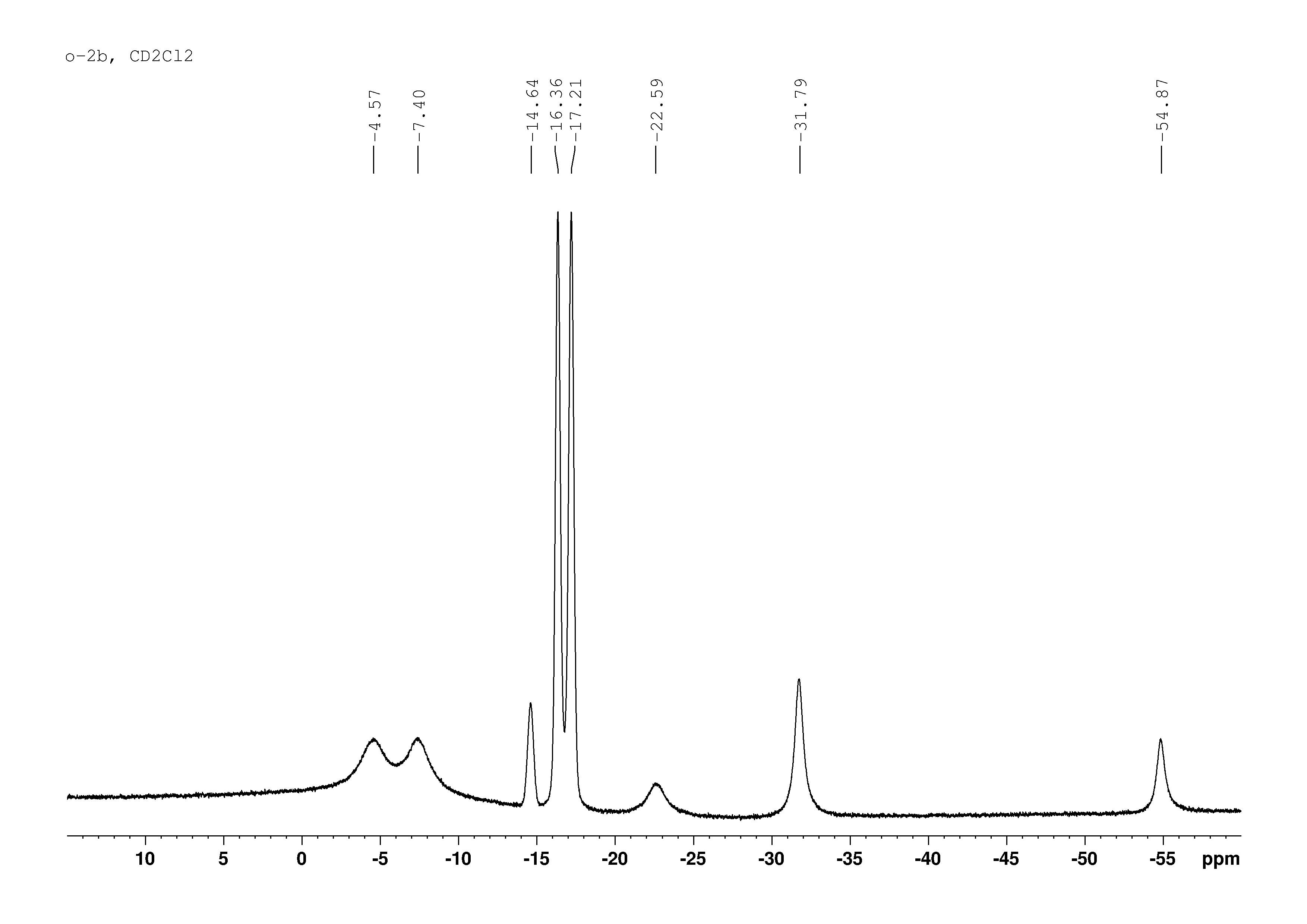


**Figure S4**. ^11^B{^1^H} NMR spectrum of **o-2b**.

**Figure S5**. Mass spectrum of negatively charged ions (ESI−, Orbitrap) for **o-2b**.

**Figure S6**. Spectrum of negatively charged ions (ESI−, Orbitrap @ R=500,000) for **o-2b** enlarged in the isotope cluster region (top) and simulated spectrum (bottom).

**Figure S7**. Spectrum of negatively charged ions (ESI−, Orbitrap @ R=500,000) for **o-2b** enlarged in the monoisotopic peak region (top) and simulated spectrum (bottom). Theoretical mass for B_11_H_14_^−^: *m/z* 135.21246; experimental mass: *m/z* 135.21236; mass error −0.10 ppm.

**Figure S8**. Mass spectrum of positively charged ions (ESI+, Orbitrap) for **o-2b**.

**Figure S9**. Spectrum of positively charged ions (ESI+, Orbitrap @ R=500,000) for **o-2b** enlarged in the isotope cluster region (top) and simulated spectrum (bottom).

**Figure S10**. Spectrum of positively charged ions (ESI+, Orbitrap @ R=500,000) for **o-2b** enlarged in the monoisotopic peak region (top) and simulated spectrum (bottom). Theoretical mass for C_56_H_83_B_8_N_4_^+^: *m/z* 899.73567; experimental mass: *m/z* 899.73705; mass error 1.53 ppm.

**Synthesis of [6,9-I^Dipp^_2_-5,10-C_2_B_8_H_11_]_2_[B_12_H_12_] (*o-*2c)**

A solution of Na_2_[B_12_H_12_] (22 mg, 0.12 mmol) in acetonitrile (2 mL) was added to the stirred solution of ***o-*2a** (101 mg, 0.12 mmol) in acetonitrile (2 mL) at room temperature, and the suspension was stirred for one day. The volatiles were removed in *vacuo,* and the solid was extracted with dichloromethane (2 mL). The volatiles were removed in *vacuo* giving ***o-*2c** as a white powder. Yield 224 mg, 93 %. **Mp.** 255 °C. **^1^H NMR** (25 °C, CD_3_CN, 500 MHz): *δ* = 0.59 (s, 2H, BC*H*), 1.07 (d, *^3^J*(^1^H, ^1^H) = 6.88 Hz, 12H, CH(C*H*_3_)_2_), 1.10 (d, *^3^J*(^1^H, ^1^H) = 6.88 Hz, 12H, CH(C*H*_3_)_2_), 1.14 (d, *^3^J*(^1^H, ^1^H) = 6.85 Hz, 12H, CH(C*H*_3_)_2_), 1.18 (d, *^3^J*(^1^H, ^1^H) = 6.85 Hz, 12H, CH(C*H*_3_)_2_), 2.19 (sept, *^3^J*(^1^H, ^1^H) = 6.86 Hz, 4H, C*H*(CH_3_)_2_), 2.40 (sept, *^3^J*(^1^H, ^1^H) = 6.86 Hz, 4H, C*H*(CH_3_)_2_), 7.31 (d, *^3^J*(^1^H, ^1^H) = 7.76 Hz, 4H, *m*-C_6_*H*_3_), 7.32 (d, *^3^J*(^1^H, ^1^H) = 7.76 Hz, 4H, *m*-C_6_*H*_3_), 7.41 (s, 4H, C*H*=C*H*), 7.57 (t, *^3^J*(^1^H, ^1^H) = 7.79 Hz, 4H, *p*-C_6_*H*_3_) ppm. **^13^C{^1^H} NMR** (25 °C, CD_3_CN, 125.76 MHz): *δ* = 22.6, 23.4, 25.6, 25.8 (s, CH(*C*H_3_)_2_), 27.0 (s, B*C*H), 29.8, 29.9 (s, *C*H(CH_3_)_2_), 125.1 (s, *C*H=*C*H), 125.8, 126.0 (s, *m*-*C*_6_H_3_), 132.4 (s, *p*-*C*_6_H_3_), 133.4 (s, *ipso*-*C*_6_H_3_), 146.3, 146.8 (s, *o*-*C*_6_H_3_), 160.5 (s very broad, N*C*N) ppm. **^11^B NMR** (25 °C, CD_3_CN, 160.46 MHz) *δ* = −54.4 (d, ^1^*J*(^1^H, ^11^B) = 153.38 Hz, 1B, B3), −31.0 (d, ^1^*J*(^1^H, ^11^B) = 119.68 Hz, 2B, B6,9), −22.3 (s broad, 1B, B1), −15.4 (d, ^1^*J*(^1^H,^11^B) = 124.43 Hz, 12B, B1´), −6.6 (s broad, 2B, B2,4), −4.3 (s broad, 2B, B7,8) ppm.

**Spectroscopic characterization of *o-*2c.**


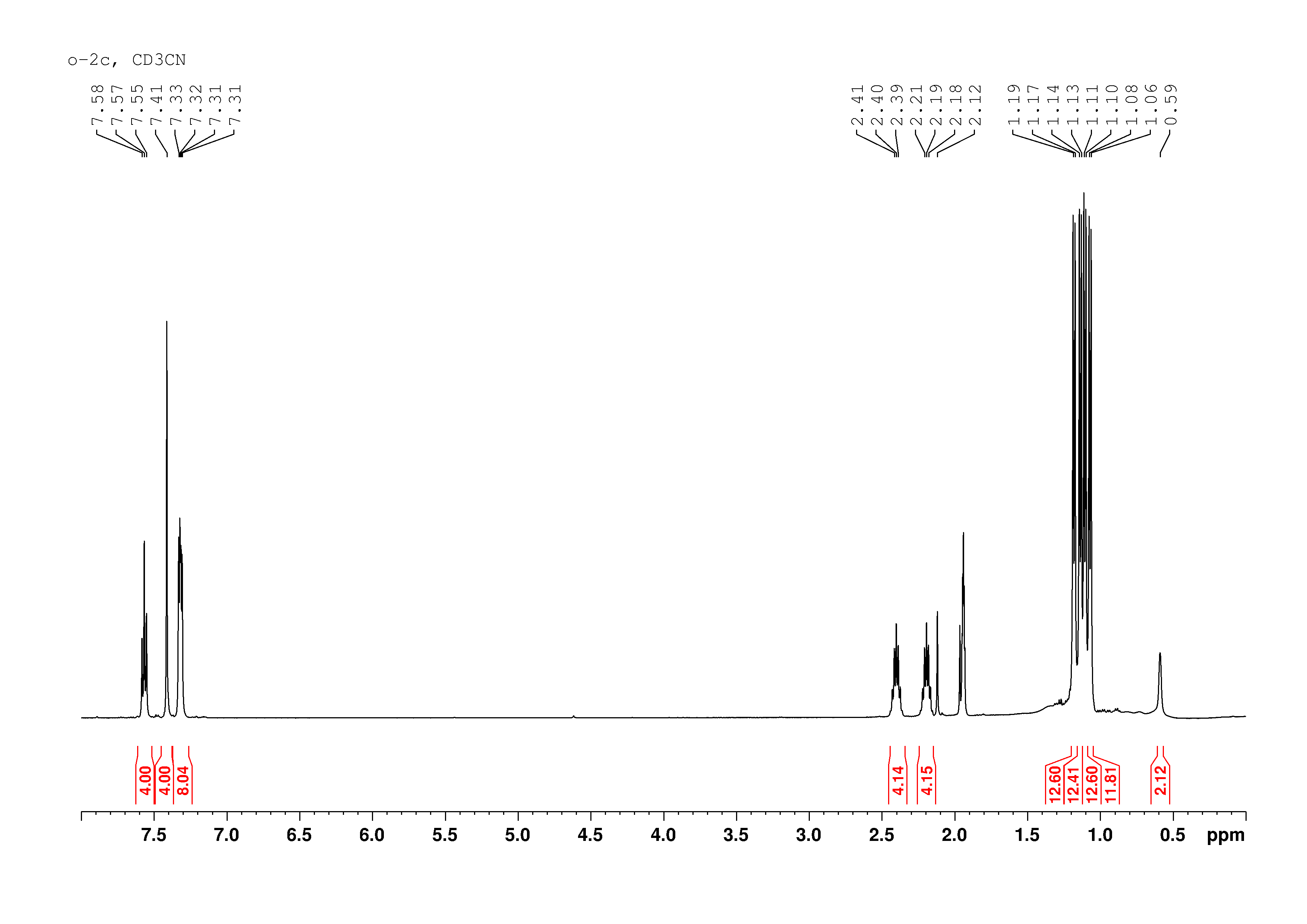


**Figure S11**. ^1^H NMR spectrum of **o-2c**.


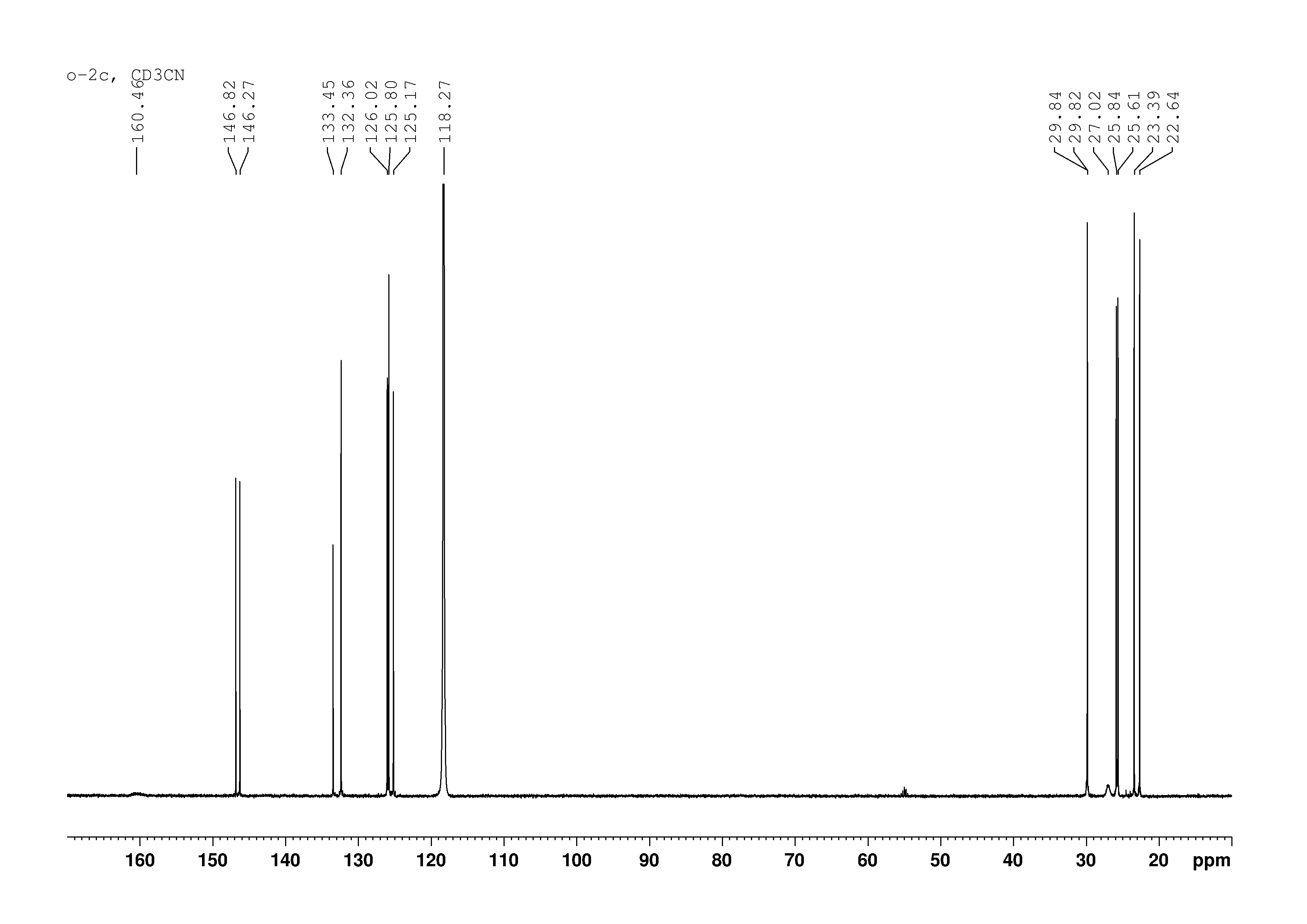


**Figure S12**. ^13^C{^1^H} NMR spectrum of **o-2c**.


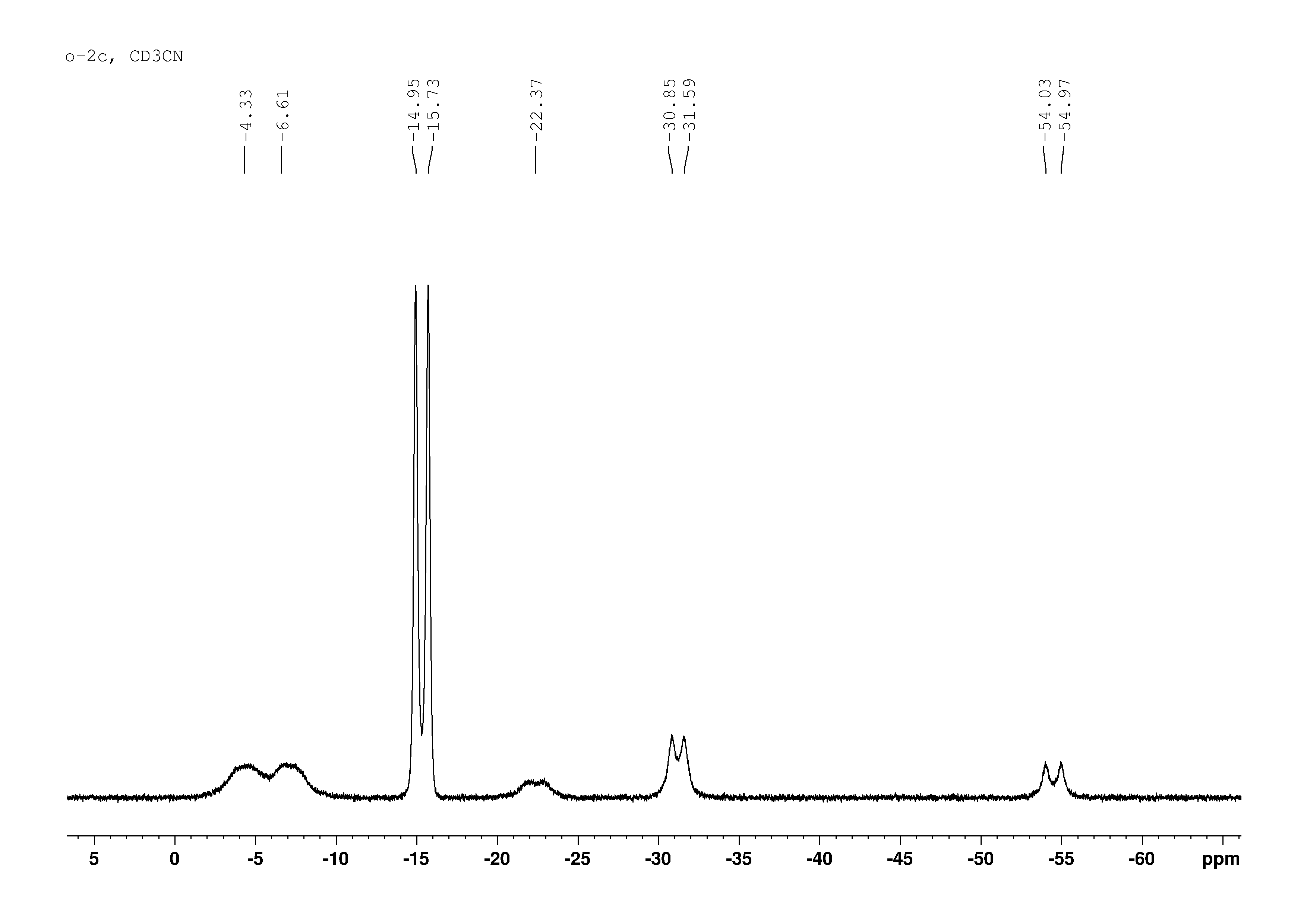


**Figure S13**. ^11^B NMR spectrum of **o-2c**.


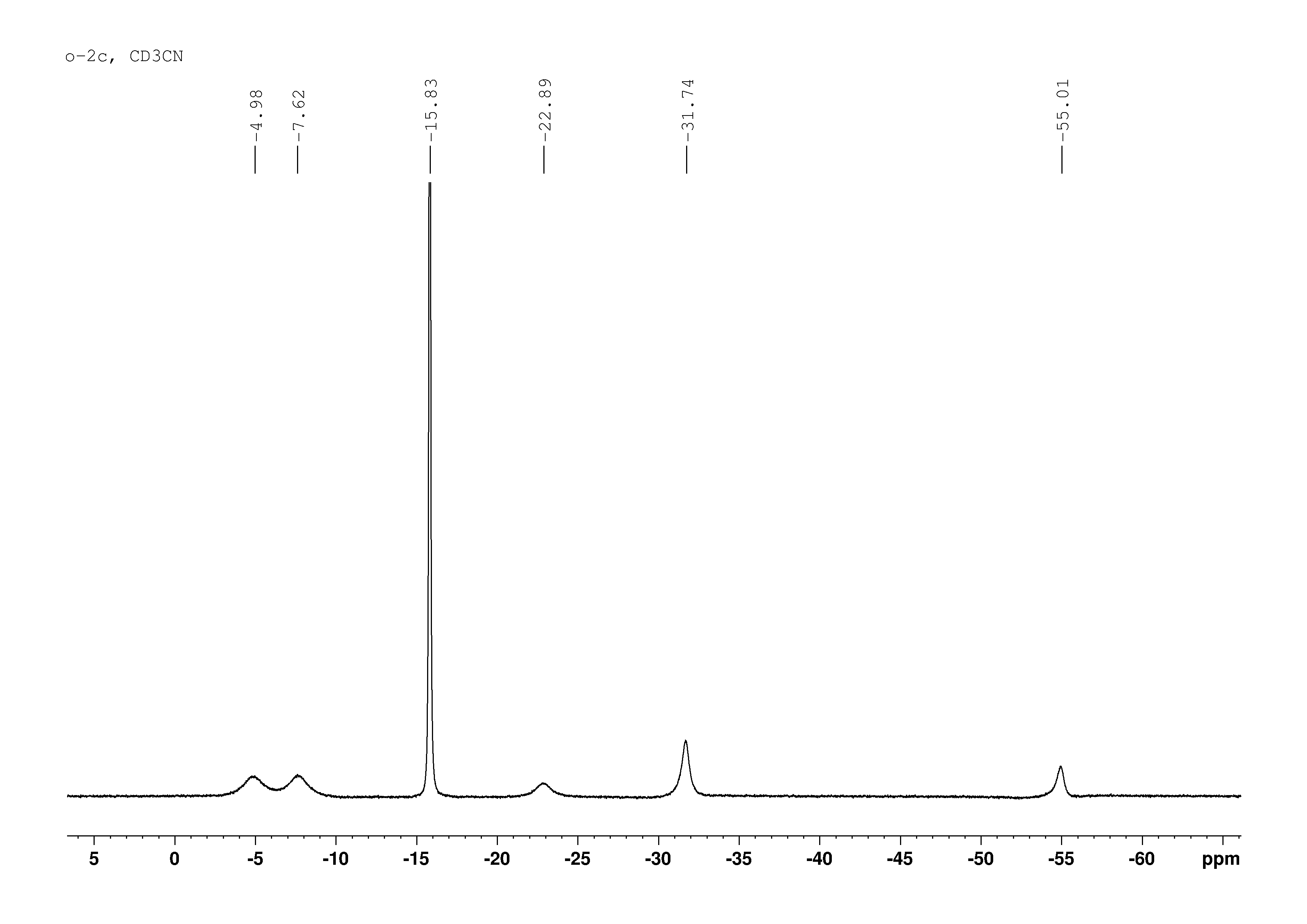


**Figure S14**. ^11^B{^1^H} NMR spectrum of **o-2c**.

**Figure S15**. Mass spectrum of negatively charged ions (ESI−, Orbitrap) for ***o*-2c**.

**Figure S16**. Spectrum of negatively charged ions (ESI−, Orbitrap @ R=500,000) for ***o*-2c** enlarged in the isotope cluster region (top) and simulated spectrum (bottom). The likely cause of the isotope cluster distortion is the progressive attenuation of lower-mass isotopologues due to the low-mass cutoff imposed by the RF settings of the transfer ion optics.

**Figure S17**. Spectrum of negatively charged ions (ESI−, Orbitrap @ R=500,000) for ***o*-2c** enlarged in the monoisotopic peak region (top) and simulated spectrum (bottom). Theoretical mass for B_12_H_12_^2−^: *m/z* 72.10333; experimental mass: *m/z* 72.10323; mass error −1.35 ppm.

**Figure S18**. Mass spectrum of positively charged ions (ESI+, Orbitrap) for ***o*-2c**.

**Figure S19**. Spectrum of positively charged ions (ESI+, Orbitrap @ R=500,000) for ***o*-2c** enlarged in the isotope cluster region (top) and simulated spectrum (bottom).

**Figure S20**. Spectrum of positively charged ions (ESI+, Orbitrap @ R=500,000) for ***o*-2c** enlarged in the monoisotopic peak region (top) and simulated spectrum (bottom). Theoretical mass for C_56_H_83_B_8_N_4_^+^: *m/z* 899.73567; experimental mass: *m/z* 899.73655; mass error 0.98 ppm.

**Synthesis of [6,9-I^Dipp^_2_-5,10-C_2_B_8_H_11_][1-CB_11_H_12_] (*o-*2d)**

A solution of Ag[CB_11_H_12_] (30 mg, 0.12 mmol) in acetonitrile (2 mL) was added to the stirred solution of ***o-*2a** (102 mg, 0.12 mmol) in acetonitrile (2 mL) at room temperature, and the suspension was stirred for one day. The volatiles were removed in *vacuo* and extracted with dichloromethane (2 mL) giving ***o-*2d** as a white powder. Yield 114 mg, 96 %. **Mp.** 257 °C. **^1^H NMR** (25 °C, CD_2_Cl_2_, 500 MHz): *δ* = −4.18 (s broad, 1H, B*H*B), 0.60 (s, 2H, BC*H*), 1.08 (d, *^3^J*(^1^H, ^1^H) = 6.70 Hz, 12H, CH(C*H*_3_)_2_), 1.13 (d, *^3^J*(^1^H, ^1^H) = 6.85 Hz, 12H, CH(C*H*_3_)_2_), 1.14 (d, *^3^J*(^1^H, ^1^H) = 6.86 Hz, 12H, CH(C*H*_3_)_2_), 1.19 (d, *^3^J*(^1^H, ^1^H) = 6.70 Hz, 12H, CH(C*H*_3_)_2_), 2.18 (sept, *^3^J*(^1^H, ^1^H) = 6.71 Hz, 4H, C*H*(CH_3_)_2_), 2.39 (sept, *^3^J*(^1^H, ^1^H) = 6.71 Hz, 4H, C*H*(CH_3_)_2_), 7.13 (s, 4H, C*H*=C*H*), 7.27 (d, *^3^J*(^1^H, ^1^H) = 7.32 Hz, 4H, *m*-C_6_*H*_3_), 7.32 (d, *^3^J*(^1^H, ^1^H) = 7.76 Hz, 4H, *m*-C_6_*H*_3_), 7.55 (t, *^3^J*(^1^H, ^1^H) = 7.72 Hz, 4H, *p*-C_6_*H*_3_) ppm. **^13^C{^1^H} NMR** (25 °C, CD_2_Cl_2_, 125.76 MHz): *δ* = 22.6, 23.3, 25.8, 26.1 (s, CH(*C*H_3_)_2_), 26.4 (s, B*C*H), 29.4 (s, *C*H(CH_3_)_2_), 51.9 (s, *C*B11) 124.8 (s, *C*H=*C*H), 125.0, 125.2 (s, *m*-*C*_6_H_3_), 131.9 (s, *p*-*C*_6_H_3_), 132.7 (s, *ipso-C*_6_H_3_), 145.5, 146.0 (s, *o-C*_6_H_3_), 160.4 (s very broad, N*C*N) ppm. **^11^B NMR** (25 °C, CD_2_Cl_2_, 160.46 MHz) *δ* = −54.6 (d, ^1^*J*(^1^H, ^11^B) = 147.57 Hz, 1B, B3), −31.5 (d, ^1^*J*(^1^H, ^11^B) = 105.46 Hz, 2B, B6,9), −22.7 (s broad, 1B, B1), −15.5 (d, ^1^*J*(^1^H, ^11^B) = 168.59 Hz, 5B, B2´−B6´), −14.6 (d, ^1^*J*(^1^H, ^11^B) = 134.95 Hz, 5B, B7´−B11´), −6.4 (d, ^1^*J*(^1^H, ^11^B) = 132.26 Hz, 2B, B12´), −4.6 (s broad, 2B, B7,8) ppm.

**Spectroscopic characterization of *o-*2d.**


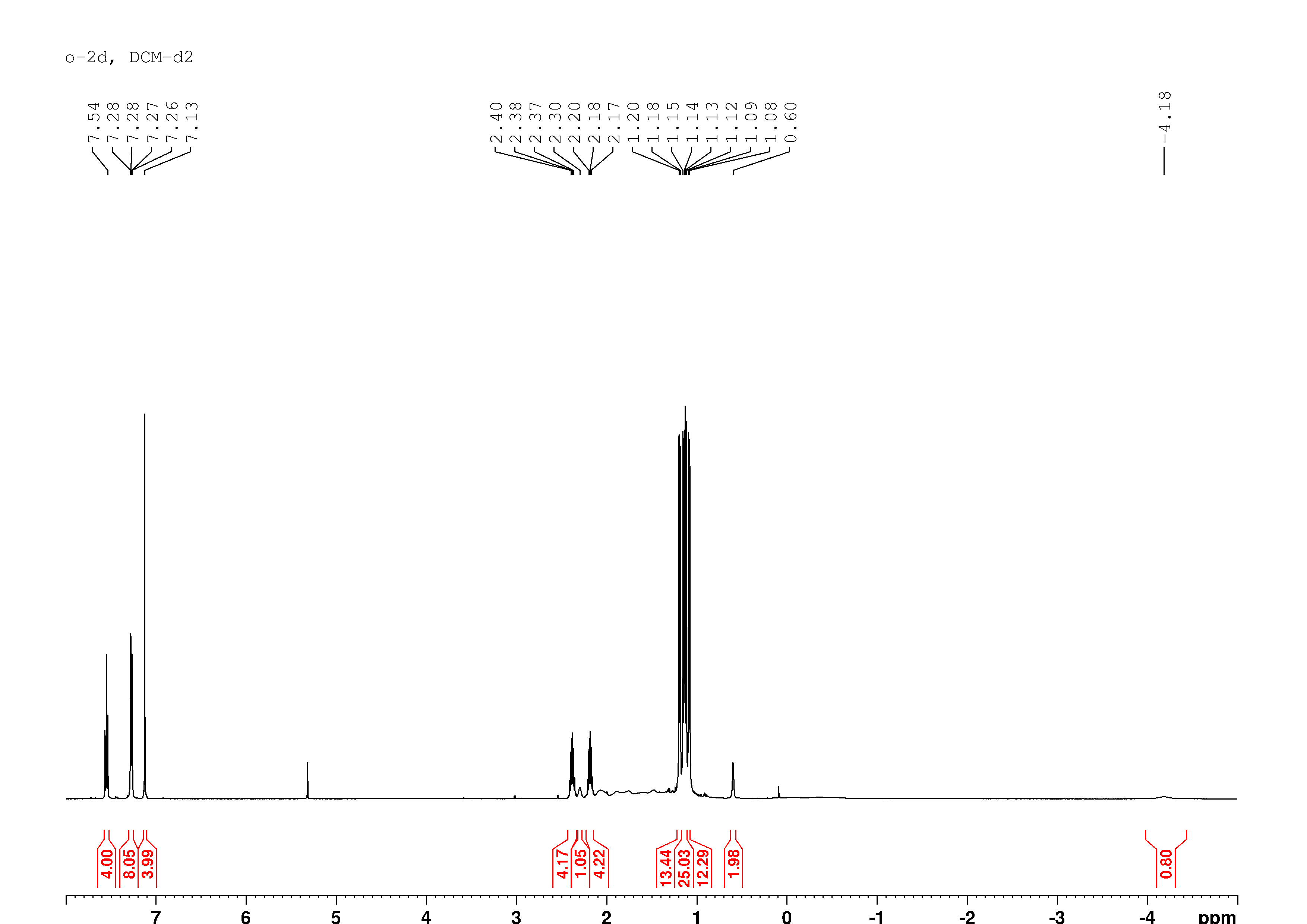


**Figure S21**. ^1^H NMR spectrum of **o-2d**.


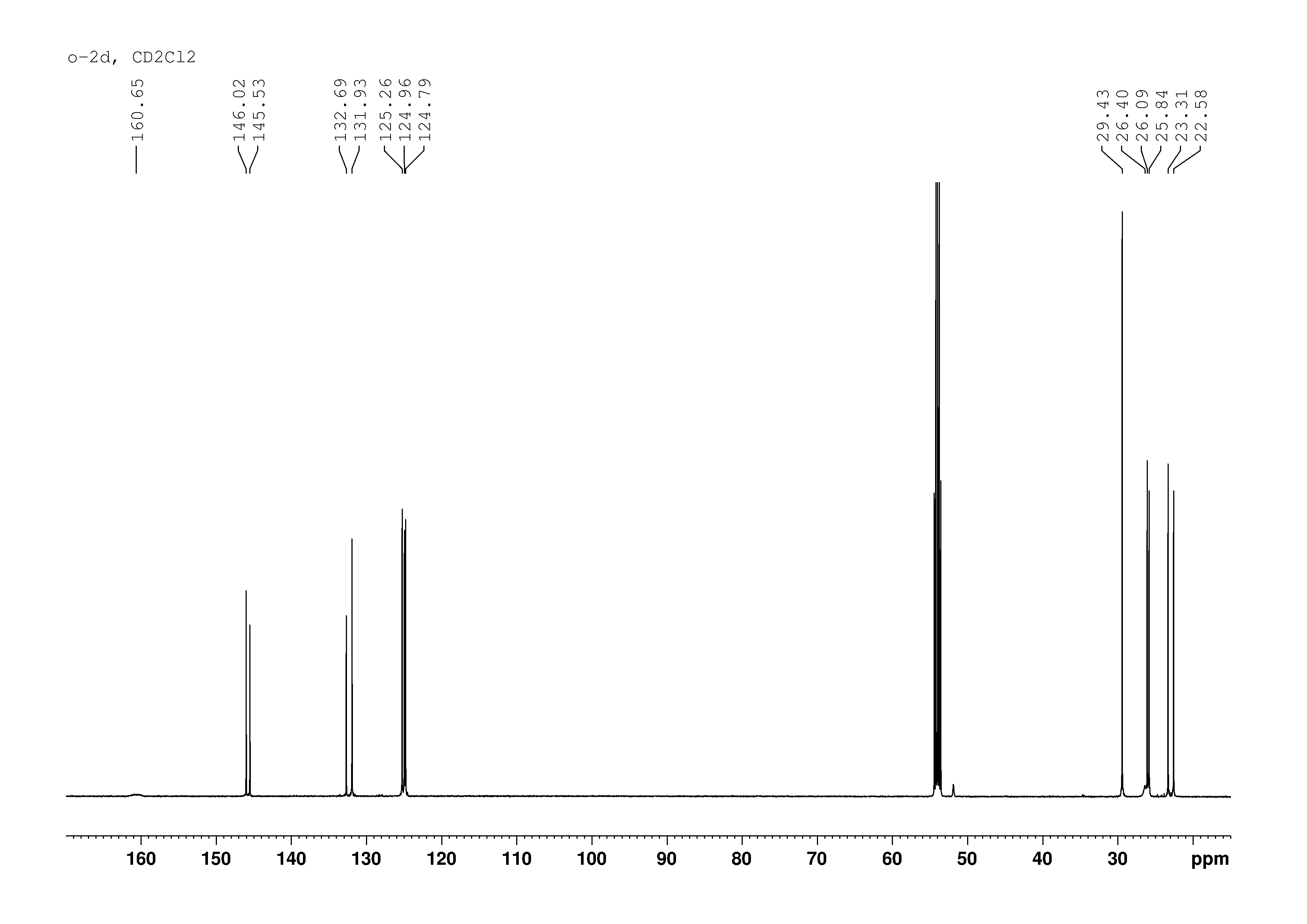


**Figure S22**. ^13^C{^1^H} NMR spectrum of **o-2d**.


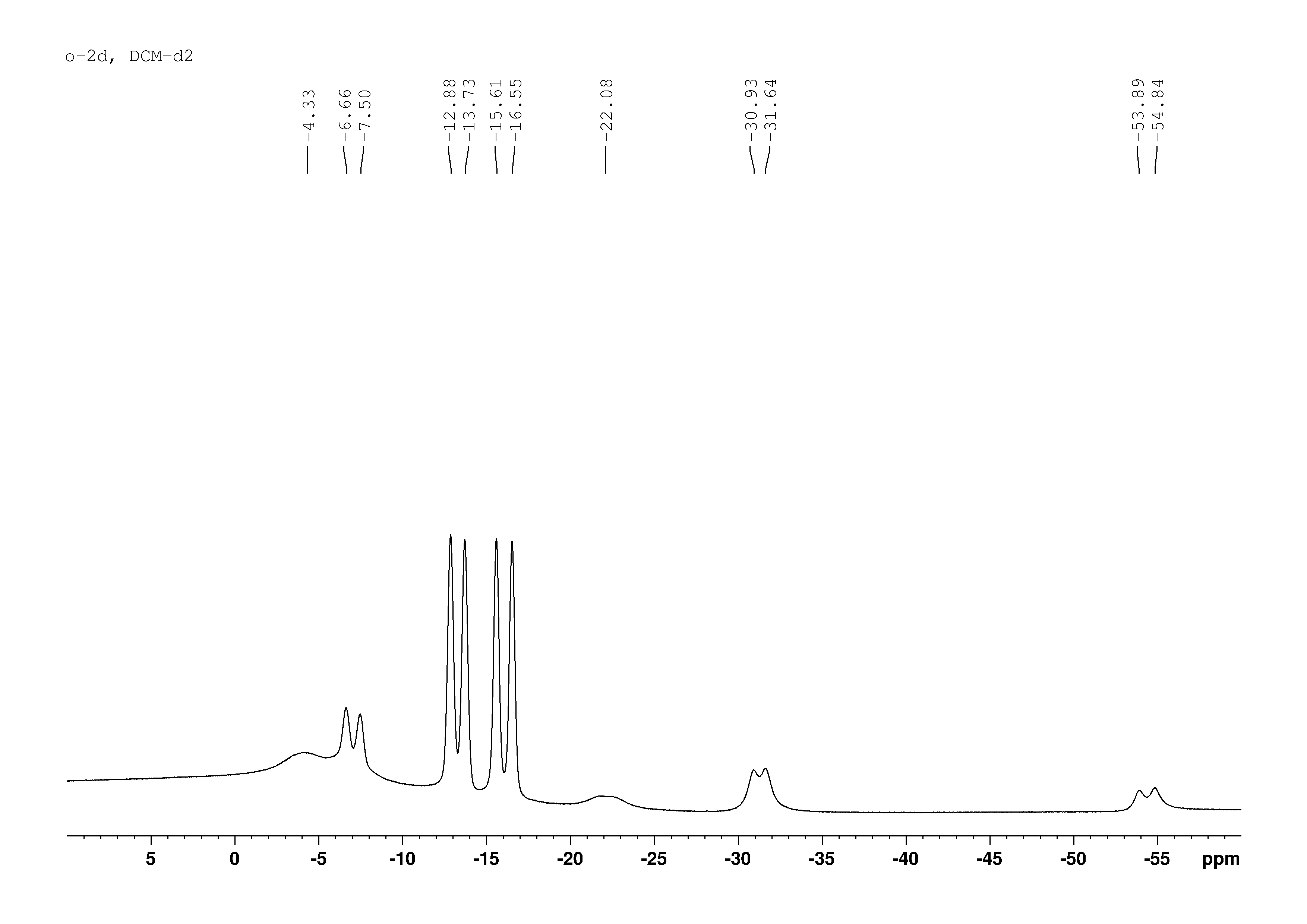


**Figure S23**. ^11^B NMR spectrum of **o-2d**.


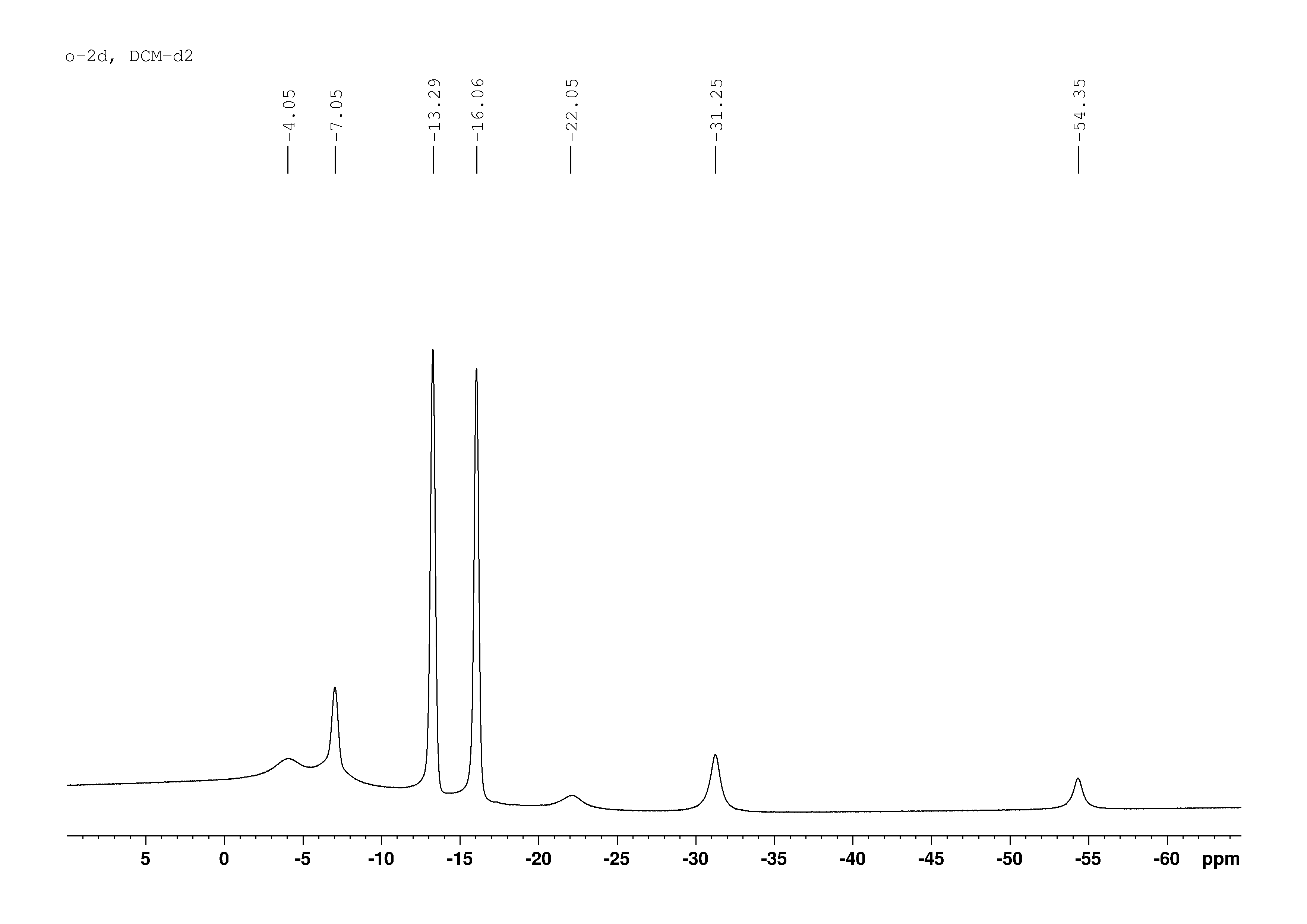


**Figure S24**. ^11^B{^1^H} NMR spectrum of **o-2d**.

**Figure S25**. Mass spectrum of negatively charged ions (ESI−, Orbitrap) for **o-2d**.

**Figure S26**. Spectrum of negatively charged ions (ESI−, Orbitrap @ R=500,000) for **o-2d** enlarged in the isotope cluster region (top) and simulated spectrum (bottom).

**Figure S27**. Spectrum of negatively charged ions (ESI−, Orbitrap @ R=500,000) for **o-2d** enlarged in the monoisotopic peak region (top) and simulated spectrum (bottom). Theoretical mass for CB_11_H_12_^−^: *m/z* 145.19681; experimental mass: *m/z* 145.19671; mass error −0.66 ppm.

**Figure S28**. Mass spectrum of positively charged ions (ESI+, Orbitrap) for **o-2d**.

**Figure S29**. Spectrum of positively charged ions (ESI+, Orbitrap @ R=500,000) for **o-2d** enlarged in the isotope cluster region (top) and simulated spectrum (bottom).

**Figure S30**. Spectrum of positively charged ions (ESI+, Orbitrap @ R=500,000) for **o-2d** enlarged in the monoisotopic peak region (top) and simulated spectrum (bottom). Theoretical mass for C_56_H_83_B_8_N_4_^+^: *m/z* 899.73567; experimental mass: *m/z* 899.73699; mass error 1.47 ppm.

**Synthesis of [6,9-I^Dipp^_2_-5,10-C_2_B_8_H_10_][2,2´-Co(1,7-C_2_B_9_H_11_)_2_] (*o-*2e)**

A solution of Cs[2,2´-Co(1,7-C_2_B_9_H_11_)_2_] (53 mg, 0.12 mmol) in acetonitrile (2 mL) was added to the stirred solution of ***o-*2a** (100 mg, 0.12 mmol) in acetonitrile (2 mL) at room temperature, and the suspension was stirred for one day. The volatiles were removed in *vacuo,* and the solid was extracted with dichloromethane (2 mL) giving ***o-*2e** as orange powder. Yield 131 mg, 95 %. **Mp.** 252 °C. **^1^H NMR** (25 °C, CD_2_Cl_2_, 500 MHz): *δ* = −4.18 (s broad, 1H, B*H*B), 0.59 (s, 2H, BC*H*), 1.08 (d, *^3^J*(^1^H, ^1^H) = 6.85 Hz, 12H, CH(C*H*_3_)_2_), 1.11 (d, *^3^J*(^1^H, ^1^H) = 6.85 Hz, 12H, CH(C*H*_3_)_2_), 1.14 (d, *^3^J*(^1^H, ^1^H) = 6.80 Hz, 12H, CH(C*H*_3_)_2_), 1.19 (d, *^3^J*(^1^H, ^1^H) = 6.80 Hz, 12H, CH(C*H*_3_)_2_), 2.18 (sept, *^3^J*(^1^H, ^1^H) = 6.85 Hz, 4H, C*H*(CH_3_)_2_), 2.38 (sept, *^3^J*(^1^H, ^1^H) = 6.86 Hz, 4H, C*H*(CH_3_)_2_), 7.11 (s, 4H, C*H*=C*H*), 7.27 (d, *^3^J*(^1^H, ^1^H) = 7.76 Hz, 4H, *m*-C_6_*H*_3_), 7.28 (d, *^3^J*(^1^H, ^1^H) = 7.93 Hz, 4H, *m*-C_6_*H*_3_), 7.57 (t, *^3^J*(^1^H, ^1^H) = 7.78 Hz, 4H, *p*-C_6_*H*_3_) ppm. **^13^C{^1^H} NMR** (25 °C, CD_2_Cl_2_, 125.76 MHz): *δ* = 22.6, 23.3, 25.9, 26.1 (s, CH(*C*H_3_)_2_), 26.5 (s, B*C*H), 29.5 (s, *C*H(CH_3_)_2_), 56.4 (s broad, *C*_2_B_9_), 124.8 (s, *C*H=*C*H), 124.9, 125.3 (s, *m*-*C*_6_H_3_), 132.0 (s, *p*-*C*_6_H_3_), 132.7 (s, *ipso-C*_6_H_3_), 145.6, 146.0 (s, *o-C*_6_H_3_), 160.8 (s very broad, N*C*N) ppm. **^11^B NMR** (25 °C, CD_2_Cl_2_, 160.46 MHz) *δ* = −54.7 (d, ^1^*J*(^1^H, ^11^B) = 147.70 Hz, 1B, B3), −31.7 (d, ^1^*J*(^1^H, ^11^B) = 123.67 Hz, 2B, B6,9), −22.9 (s broad, 1B, B1), −18.0 (d, ^1^*J*(^1^H, ^11^B) = 155.0 Hz, 2B, B6´,11´), −12.5 (d, ^1^*J*(^1^H, ^11^B) = 149.2 Hz, 3B, 5´,9´,12´), −9.5 (d, ^1^*J*(^1^H, ^11^B) = 162.97 Hz, 1B, B3´), −7.2 (s broad, 2B, B2,4), −4.6 (s broad, 2B, B7,8), −2.8 (d, ^1^*J*(^1^H, ^11^B) = 153.5 Hz, 2B, B4´,8´), 0.7 (d, ^1^*J*(^1^H, ^11^B) = 142.8 Hz, 1B, B10´) ppm.

**Spectroscopic characterization of *o-*2e.**


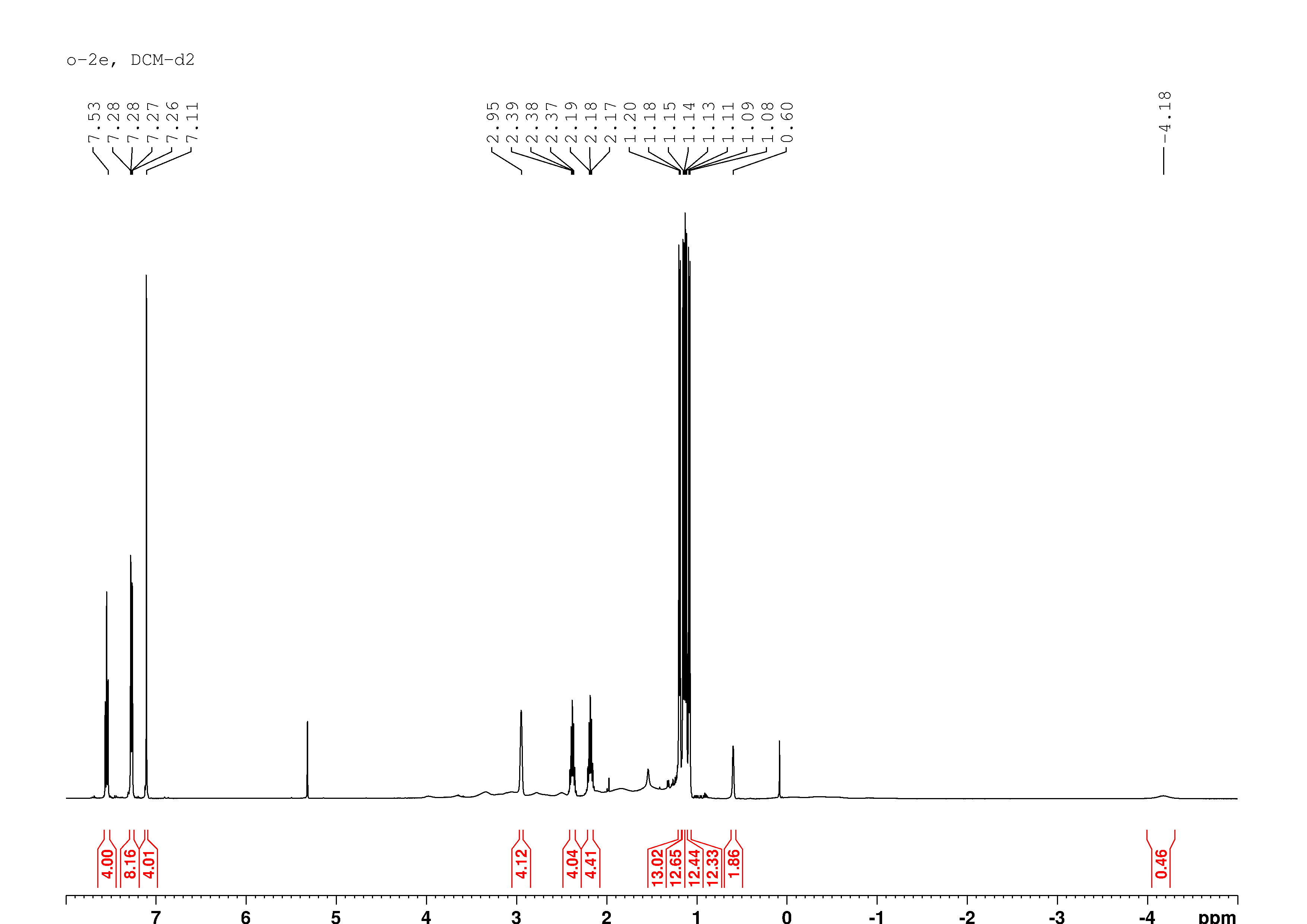


**Figure S31**. ^1^H NMR spectrum of **o-2e**.


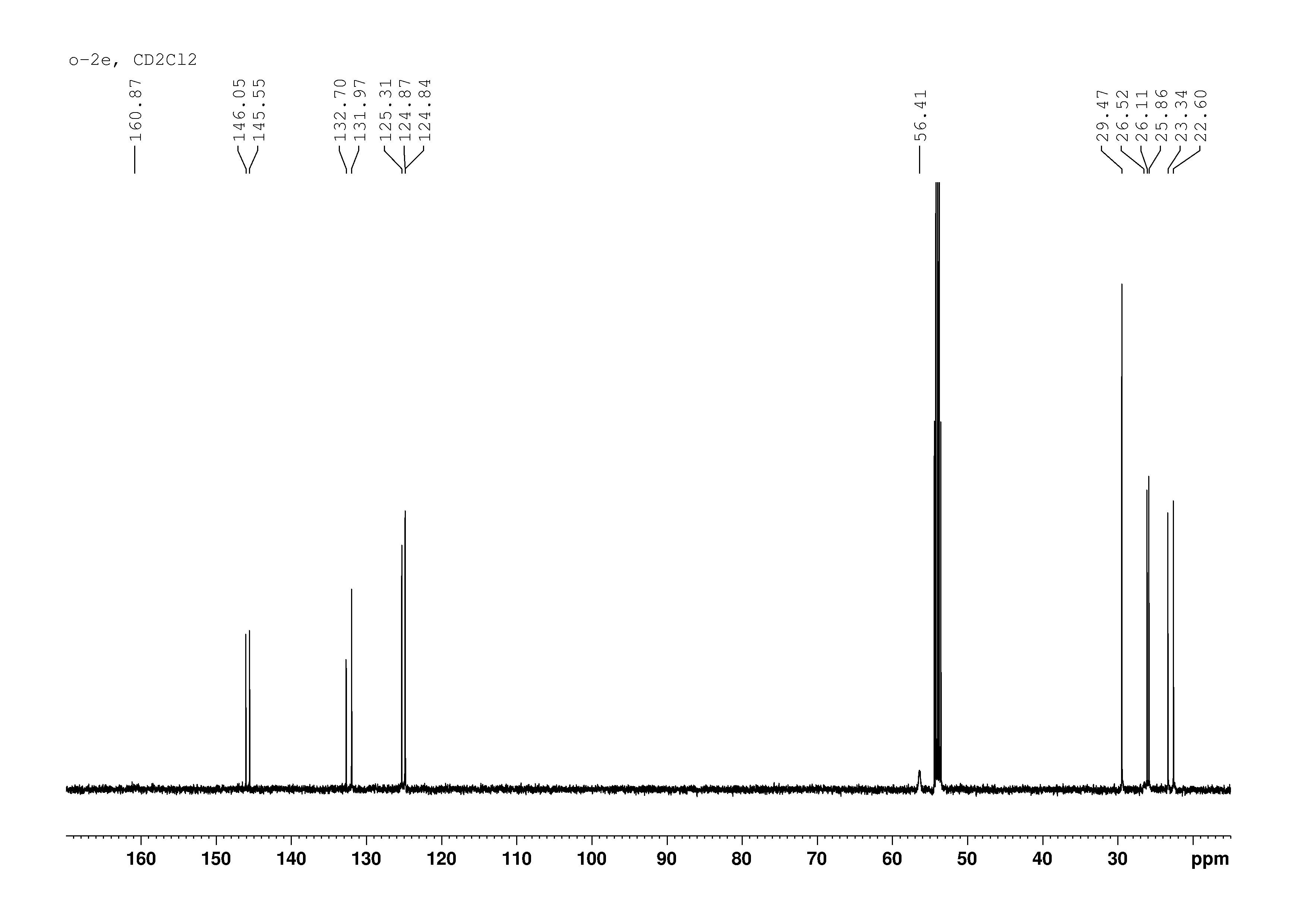


**Figure S32**. ^13^C{^1^H} NMR spectrum of **o-2e**.


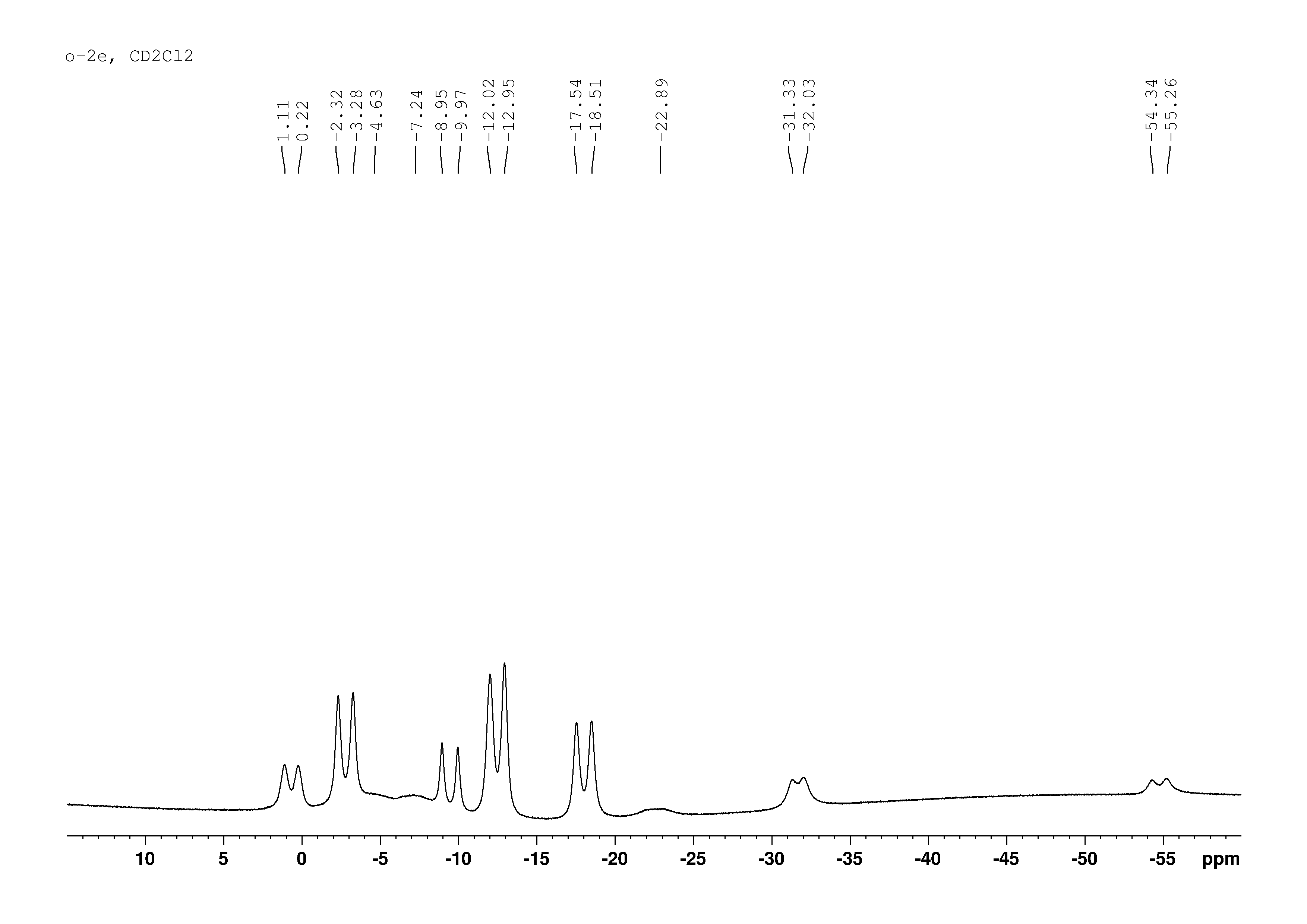


**Figure S33**. ^11^B NMR spectrum of **o-2e**.


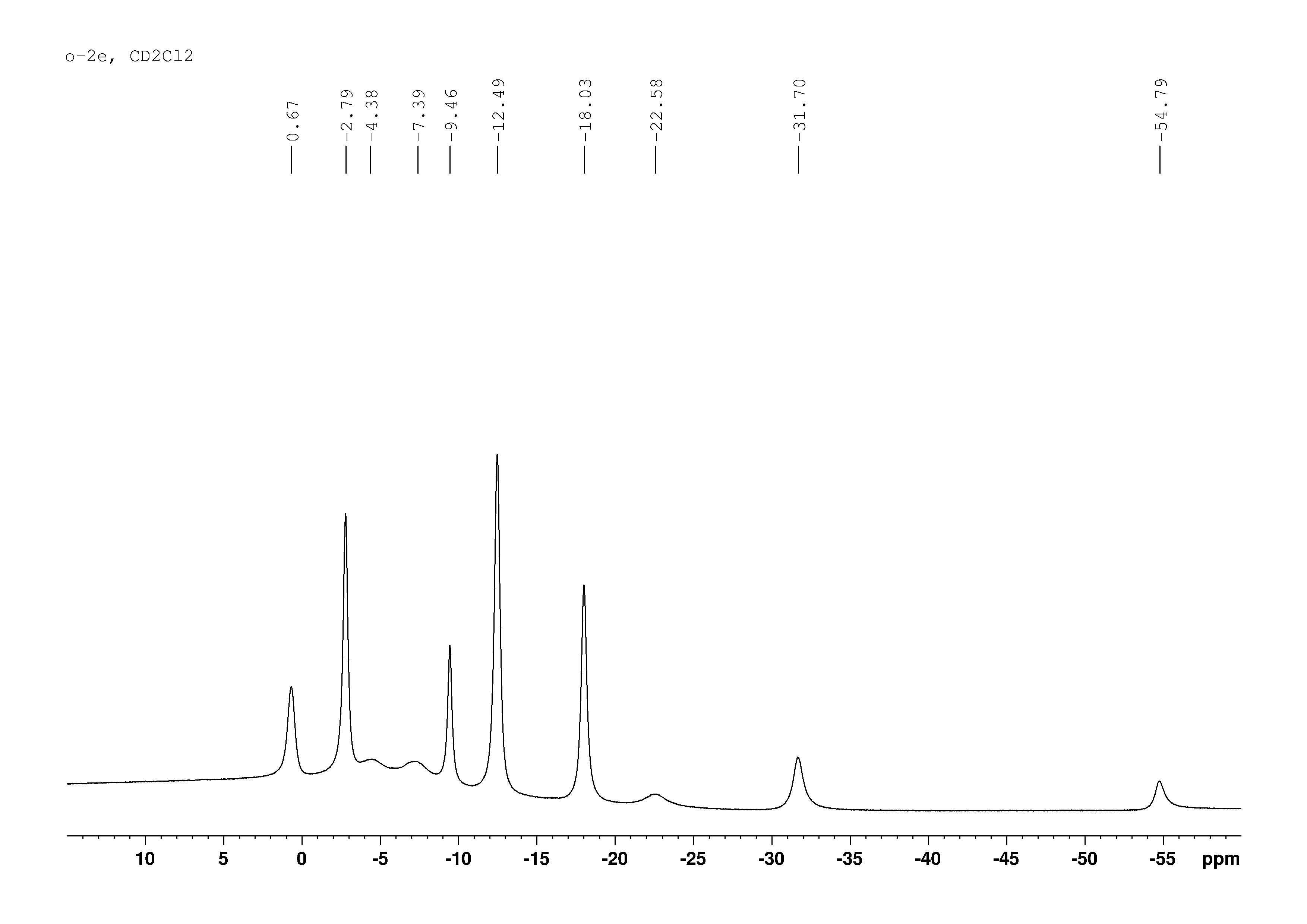


**Figure S34**. ^11^B{^1^H} NMR spectrum of **o-2e**.

**Figure S35**. Mass spectrum of negatively charged ions (ESI−, Orbitrap) for **o-2e**.

**Figure S36**. Spectrum of negatively charged ions (ESI−, Orbitrap @ R=500,000) for **o-2e** enlarged in the isotope cluster region (top) and simulated spectrum (bottom).

**Figure S37**. Spectrum of negatively charged ions (ESI−, Orbitrap @ R=500,000) for **o-2e** enlarged in the monoisotopic peak region (top) and simulated spectrum (bottom). Theoretical mass for C_4_B_18_H_22_Co^−^: *m/z* 327.27340; experimental mass: *m/z* 327.27297; mass error −1.31 ppm.

**Figure S38**. Mass spectrum of positively charged ions (ESI+, Orbitrap) for **o-2e**.

**Figure S39**. Spectrum of positively charged ions (ESI+, Orbitrap @ R=500,000) for **o-2e** enlarged in the isotope cluster region (top) and simulated spectrum (bottom).

**Figure S40**. Spectrum of positively charged ions (ESI+, Orbitrap @ R=500,000) for **o-2e** enlarged in the monoisotopic peak region (top) and simulated spectrum (bottom). Theoretical mass for C_56_H_83_B_8_N_4_^+^: *m/z* 899.73567; experimental mass: *m/z* 899.73734; mass error 1.85 ppm.

**Synthesis of [6,9-I^Dipp^_2_-5,10-C_2_B_8_H_11_][3,3´-Fe(1,2-C_2_B_9_H_11_)_2_] (*o-*2f)**

A solution of Cs[3,3´-Fe(1,2-C_2_B_9_H_11_)_2_] (58 mg, 0.13 mmol) in acetonitrile (2 mL) was added to the stirred solution of ***o-*2a** (110 mg, 0.13 mmol) in acetonitrile (2 mL) at room temperature, and the suspension was stirred for one day. The volatiles were removed in *vacuo* and the solid was extracted with dichloromethane (2 mL) giving ***o-*2f** as dark red powder. Yield 139 mg, 92 %. **Mp.** 256 °C. **^1^H NMR** (25 °C, CD_2_Cl_2_, 500 MHz): *δ* = −4.31, 0.60 (s, 2H, BC*H*), 1.08 (d, *^3^J*(^1^H, ^1^H) = 6.70 Hz, 12H, CH(C*H*_3_)_2_), 1.13 (d, *^3^J*(^1^H, ^1^H) = 6.85 Hz, 12H, CH(C*H*_3_)_2_), 1.15 (d, *^3^J*(^1^H, ^1^H) = 6.86 Hz, 12H, CH(C*H*_3_)_2_), 1.19 (d, *^3^J*(^1^H, ^1^H) = 6.70 Hz, 12H, CH(C*H*_3_)_2_), 2.19 (sept, *^3^J*(^1^H, ^1^H) = 6.71 Hz, 4H, C*H*(CH_3_)_2_), 2.39 (sept, *^3^J*(^1^H, ^1^H) = 6.86 Hz, 4H, C*H*(CH_3_)_2_), 7.13 (s, 4H, C*H*=C*H*), 7.27 (d, *^3^J*(^1^H, ^1^H) = 7.32 Hz, 4H, *m*-C_6_*H*_3_), 7.55 (t, *^3^J*(^1^H, ^1^H) = 7.72 Hz, 4H, *p*-C_6_*H*_3_) ppm. **^13^C{^1^H} NMR** (25 °C, CD_2_Cl_2_, 125.76 MHz): *δ* = 22.3, 23.0, 25.5, 25.8 (s, CH(*C*H_3_)_2_), 26.1 (s, B*C*H), 29.1 (s, *C*H(CH_3_)_2_), 124.5 (s, *C*H=*C*H), 124.7, 124.9 (s, *m*-*C*_6_H_3_), 13164 (s, *p*-*C*_6_H_3_), 132.3 (s, *ipso-C*_6_H_3_), 145.2, 145.7 (s, *o-C*_6_H_3_), 160.5 (s very broad, N*C*N) ppm. **^11^B NMR** (25 °C, CD_2_Cl_2_, 160.46 MHz) *δ* = −417.1 (s broad, 4B, B8´), −393.1 (s broad, 4B, B4´,7´), −54.5 (d, ^1^*J*(^1^H, ^11^B) = 151.08 Hz, 1B, B3), −34.4 (s broad, 4B, B5´,11´), −31.8 (d, ^1^*J*(^1^H, ^11^B) = 99.32 Hz, 2B, B6,9), −22.6 (s broad, 1B, B1), −7.1 (s broad, 2B, B2,4), −4.3 (s broad, 2B, B7,8), 1.3 (s broad, 4B, B9´,12´), 29.4 (s broad, 2B, B10´), 107.9 (s broad, 2B, B6´) ppm.

**Spectroscopic characterization of *o-*2f.**


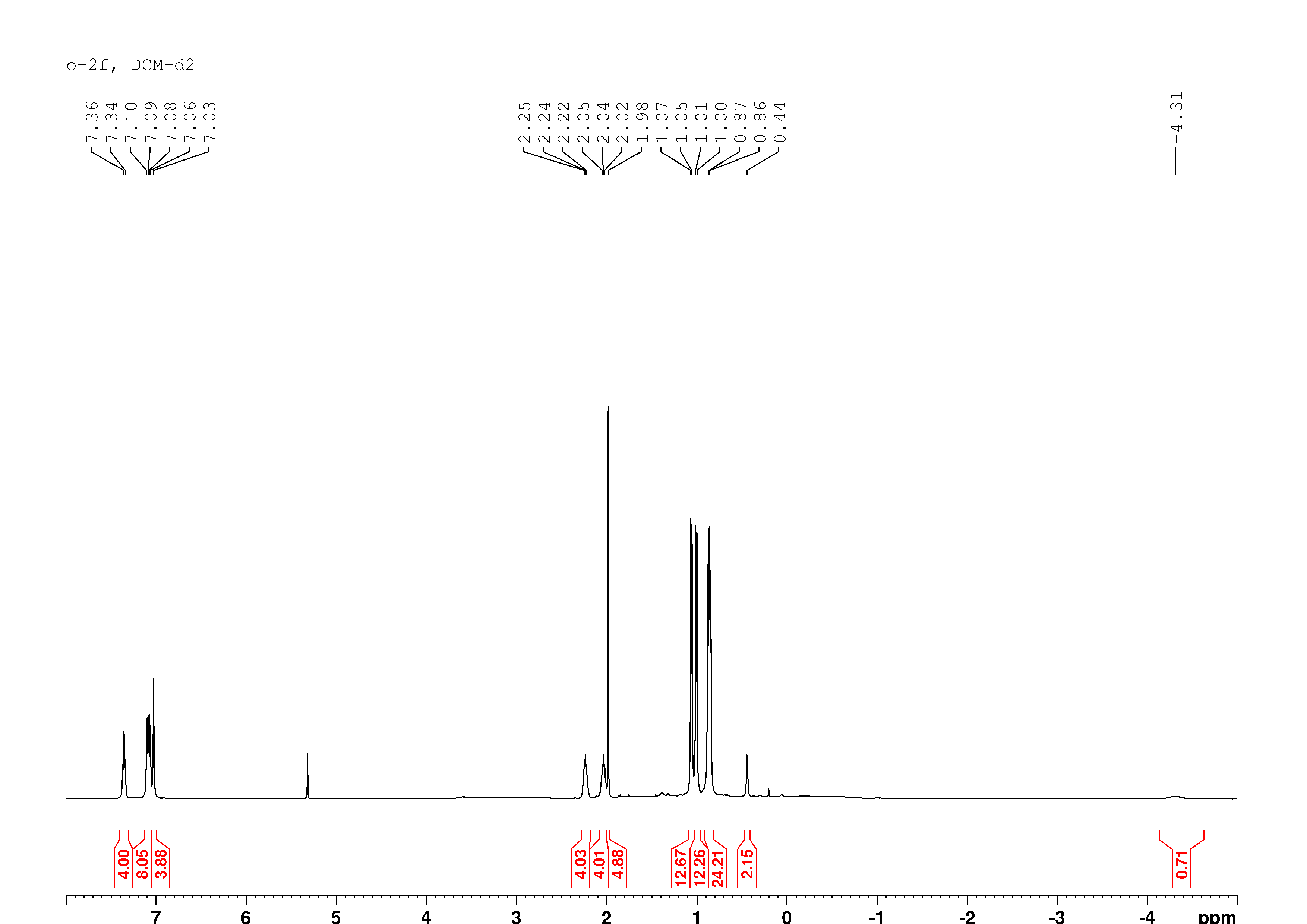


**Figure S41**. ^1^H NMR spectrum of **o-2f**.


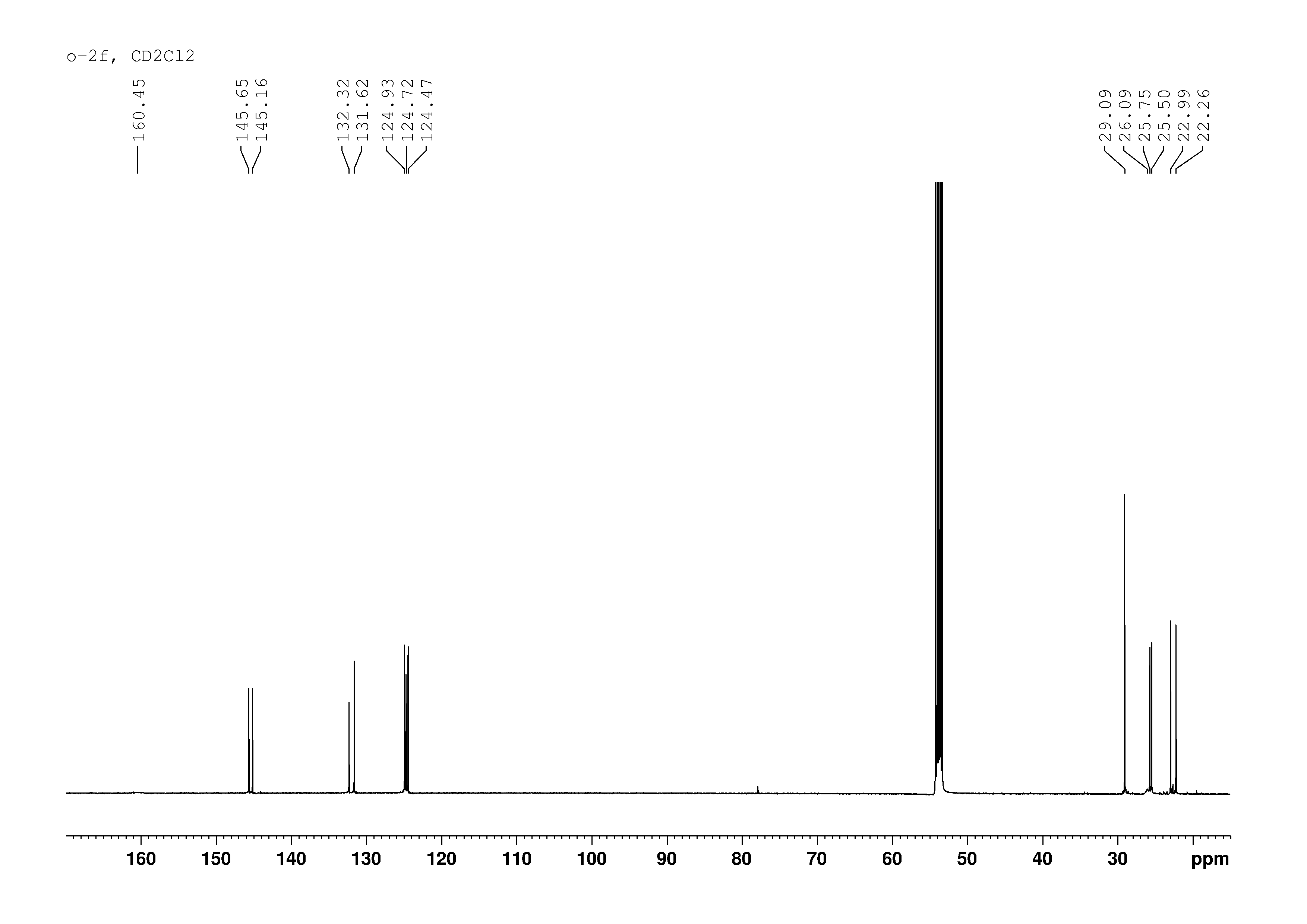


**Figure S42**. ^13^C{^1^H} NMR spectrum of **o-2f**.


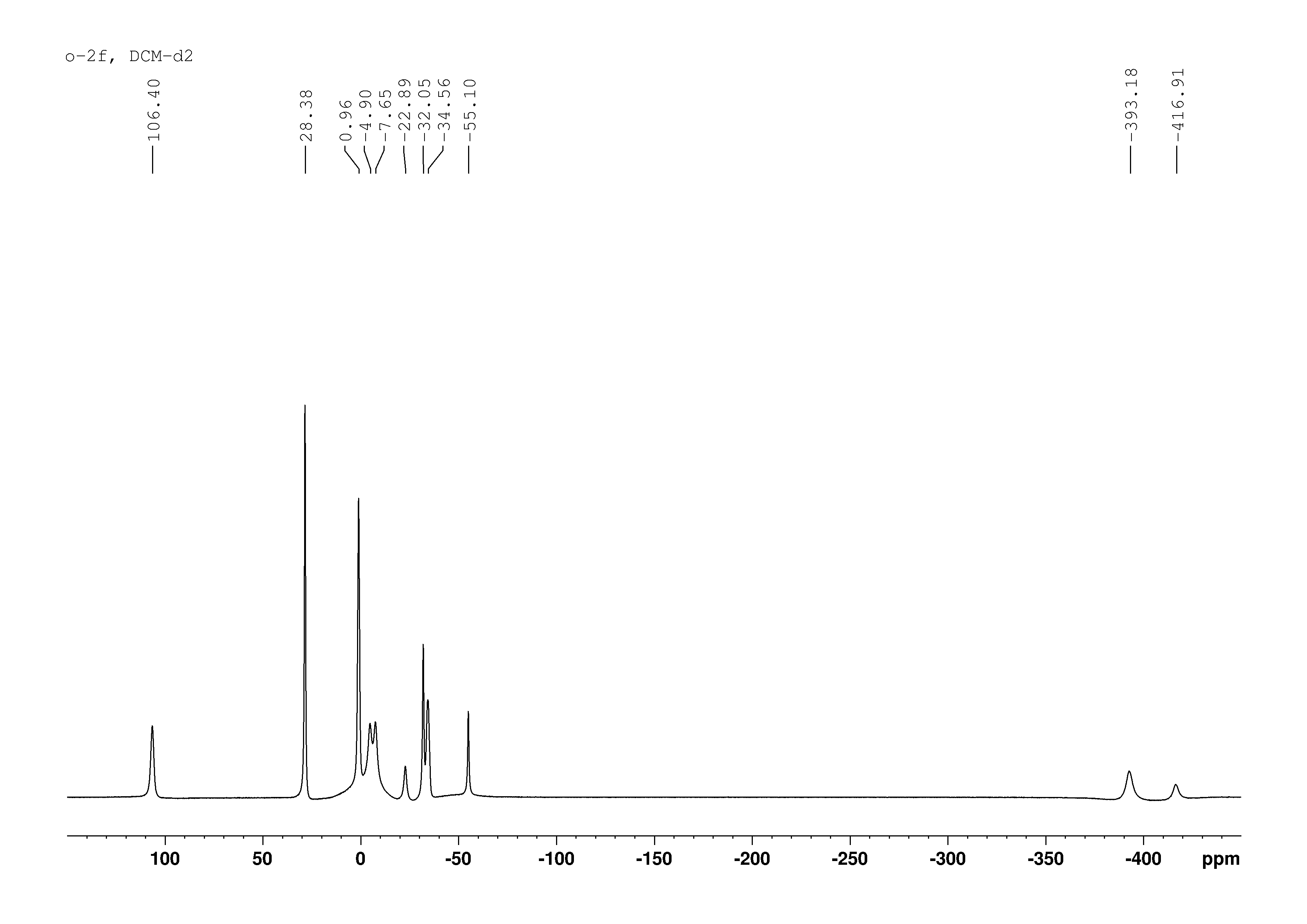


**Figure S43**. ^11^B NMR spectrum of **o-2f**.


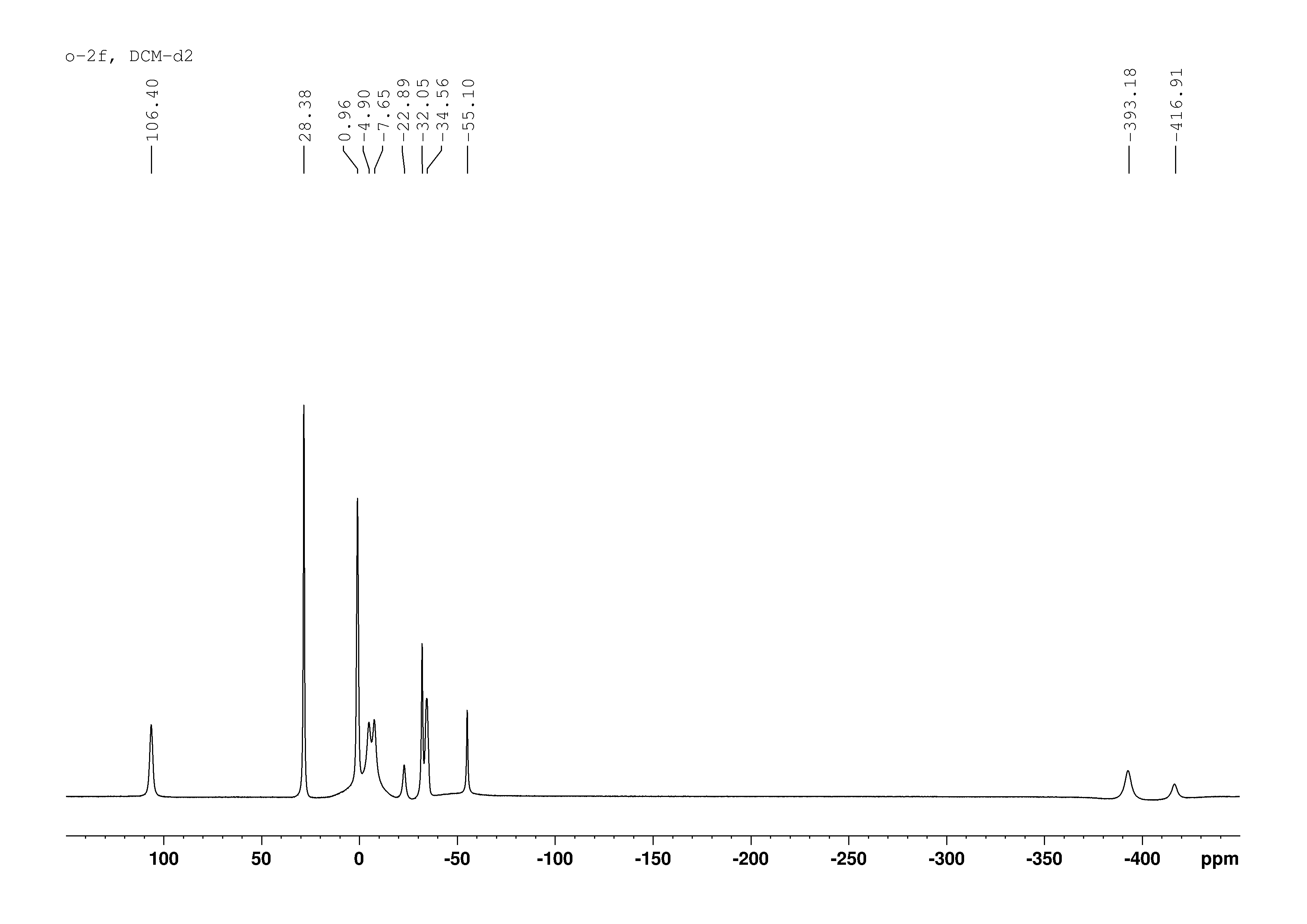


**Figure S44**. ^11^B{^1^H} NMR spectrum of **o-2f**.

**Figure S45**. Mass spectrum of negatively charged ions (ESI−, Orbitrap) for **o-2f**.

**Figure S46**. Spectrum of negatively charged ions (ESI−, Orbitrap @ R=500,000) for **o-2f** enlarged in the isotope cluster region (top) and simulated spectrum (bottom).

**Figure S47**. Spectrum of negatively charged ions (ESI−, Orbitrap @ R=500,000) for **o-2f** enlarged in the monoisotopic peak region (top) and simulated spectrum (bottom). Theoretical mass for C_4_B_18_H_22_Fe^−^: *m/z* 324.27514; experimental mass: *m/z* 324.27480; mass error −1.06 ppm.

**Figure S48**. Mass spectrum of positively charged ions (ESI+, Orbitrap) for **o-2f**.

**Figure S49**. Spectrum of positively charged ions (ESI+, Orbitrap @ R=500,000) for **o-2f** enlarged in the isotope cluster region (top) and simulated spectrum (bottom).

**Figure S50**. Spectrum of positively charged ions (ESI+, Orbitrap @ R=500,000) for **o-2f** enlarged in the monoisotopic peak region (top) and simulated spectrum (bottom). Theoretical mass for C_56_H_83_B_8_N_4_^+^: *m/z* 899.73567; experimental mass: *m/z* 899.73657; mass error 1.20 ppm.

**Synthesis of [6,9-I^Dipp^_2_-5,10-C_2_B_8_H_11_][6-SB_9_H_12_] (*o-*2g)**

A solution of Cs[6-SB_9_H_12_] (32 mg, 0.12 mmol) in acetonitrile (2 mL) was added to the stirred solution of ***o-*2a** (100 mg, 0.12 mmol) in acetonitrile at room temperature, and the suspension was stirred for one day. The volatiles were removed in *vacuo,* and the solid was extracted with dichloromethane (2 mL) giving ***o-*2g** as a white powder. Yield 111 mg, 95 %. **Mp.** > 320 °C.**^1^H NMR** (25 °C, CD_2_Cl_2_, 500 MHz): *δ* = −4.18 (s broad, 1H, B*H*B), −1.79 (s broad, 2H, B*H*B), 0.6 (s, 2H, BC*H*), 1.09 (d, *^3^J*(^1^H, ^1^H) = 6.82 Hz, 12H, CH(C*H*_3_)_2_), 1.12 (d, *^3^J*(^1^H, ^1^H) = 6.82 Hz, 12H, CH(C*H*_3_)_2_), 1.15 (d, *^3^J*(^1^H, ^1^H) = 6.83 Hz, 12H, CH(C*H*_3_)_2_), 1.19 (d, *^3^J*(^1^H, ^1^H) = 6.80 Hz, 12H, CH(C*H*_3_)_2_), 2.19 (sept, *^3^J*(^1^H, ^1^H) = 6.87 Hz, 4H, C*H*(CH_3_)_2_), 2.39 (sept, *^3^J*(^1^H, ^1^H) = 6.80 Hz, 4H, C*H*(CH_3_)_2_), 7.13 (s, 4H, C*H*=C*H*), 7.27 (d, *^3^J*(^1^H, ^1^H) = 7.93 Hz, 4H, *m*-C_6_*H*_3_), 7.55 (t, *^3^J*(^1^H, ^1^H) = 7.78 Hz, 4H, *p*-C_6_*H*_3_) ppm. **^13^C{^1^H} NMR** (25 °C, CD_2_Cl_2_, 125.76 MHz): *δ* = 22.4, 23.1, 25.7, 25.9 (s, CH(*C*H_3_)_2_), 26.2 (s, B*C*H), 29.2 (s, *C*H(CH_3_)_2_), 124.6 (s, *C*H=*C*H), 124.8, 125.1 (s, *m*-*C*_6_H_3_), 131.7 (s, *p*-*C*_6_H_3_), 132.5 (s, *ipso-C*_6_H_3_), 145.3, 145.8 (s, *o-C*_6_H_3_), 160.4 (s very broad, N*C*N) ppm. **^11^B NMR** (25 °C, CD_2_Cl_2_, 160.46 MHz) *δ* = −54.4 (d, ^1^*J*(^1^H, ^11^B) = 147.65 Hz, 1B, B3), −37.2 (d, ^1^*J*(^1^H, ^11^B) = 136.57 Hz, 2B, B1´,3´), −33.9 (d, ^1^*J*(^1^H, ^11^B) = 143.87 Hz, 2B, B8´,10´), −30.3 (d, ^1^*J*(^1^H, ^11^B) = 106.52 Hz, 2B, B6,9), −22.0 (s broad, 1B, B1), −15.9 (t, ^1^*J*(^1^H, ^11^B) = 113.6 Hz, 1B, B9´), −11.8 (d, ^1^*J*(^1^H, ^11^B) = 166.08 Hz, 1B, B2´), −8.5 (d, ^1^*J*(^1^H, ^11^B) = 148.65 Hz, 2B, B5´7´), −6.9 (s broad, 2B, B2,4), −4.3 (s broad, 2B, B7,8), 3.4 (d, ^1^*J*(^1^H, ^11^B) = 132.98 Hz, 1B, B4´) ppm.

**Spectroscopic characterization of *o-*2g.**


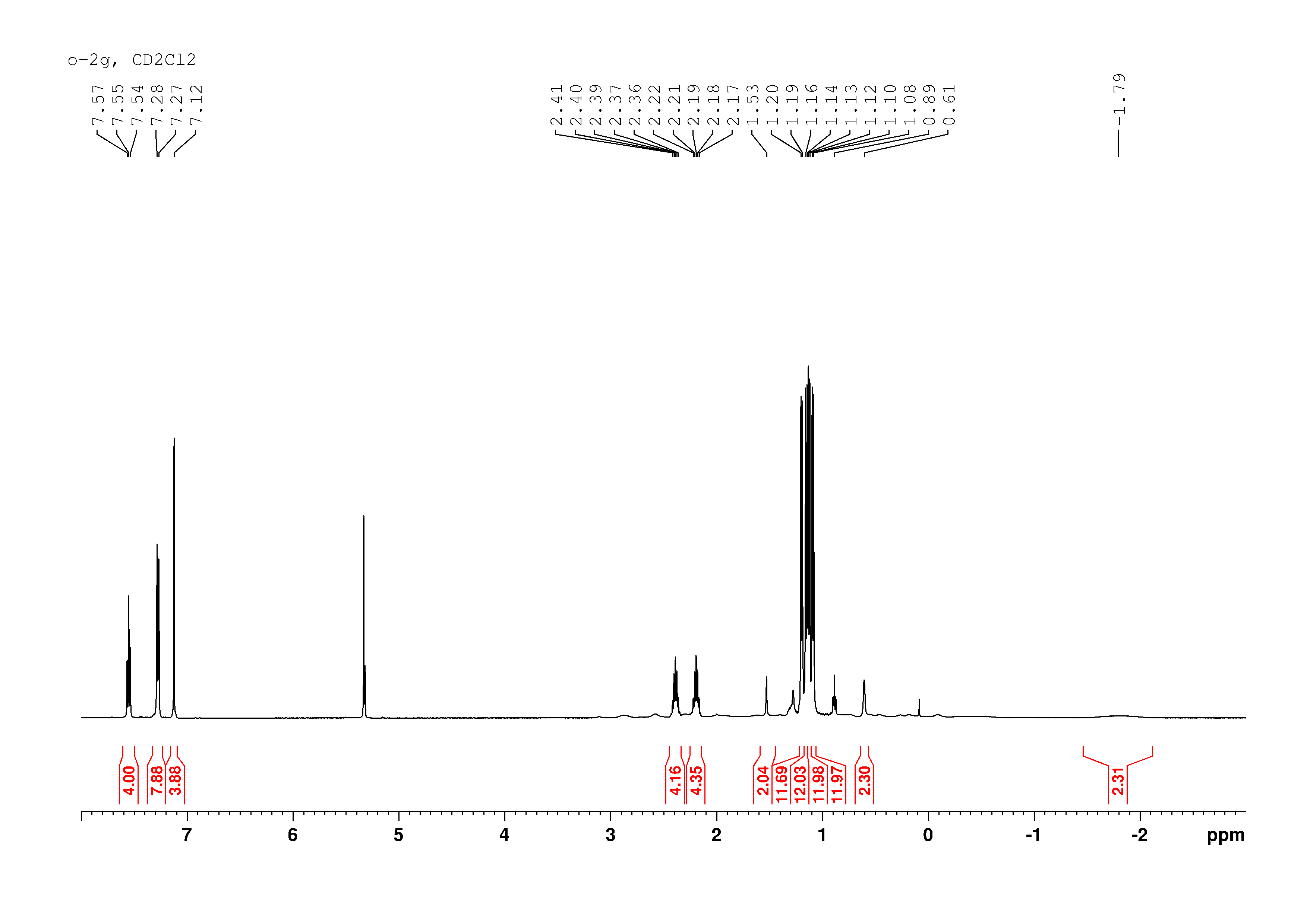


**Figure S51**. ^1^H NMR spectrum of **o-2g**.


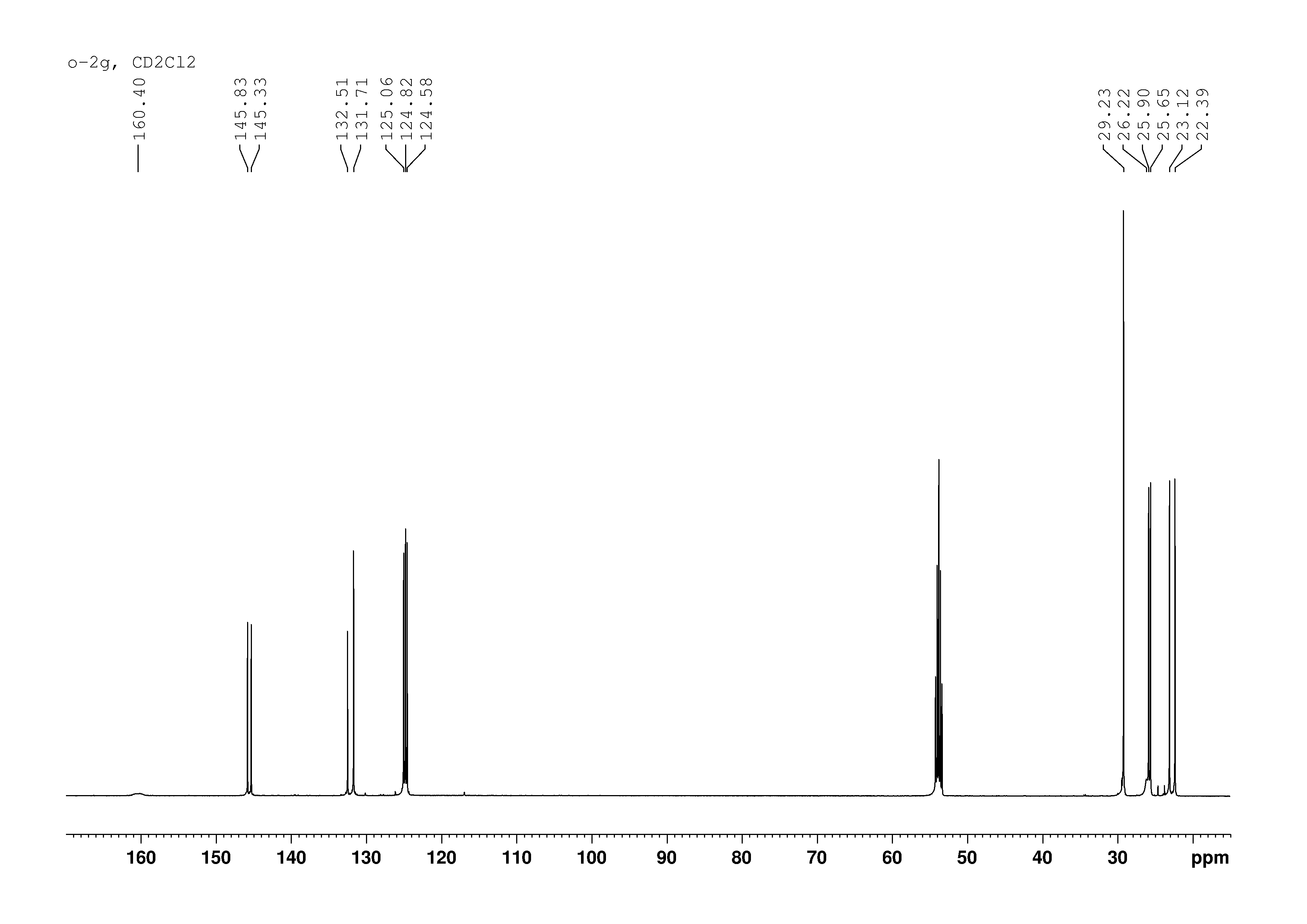


**Figure S52**. ^13^C{^1^H} NMR spectrum of **o-2g**.


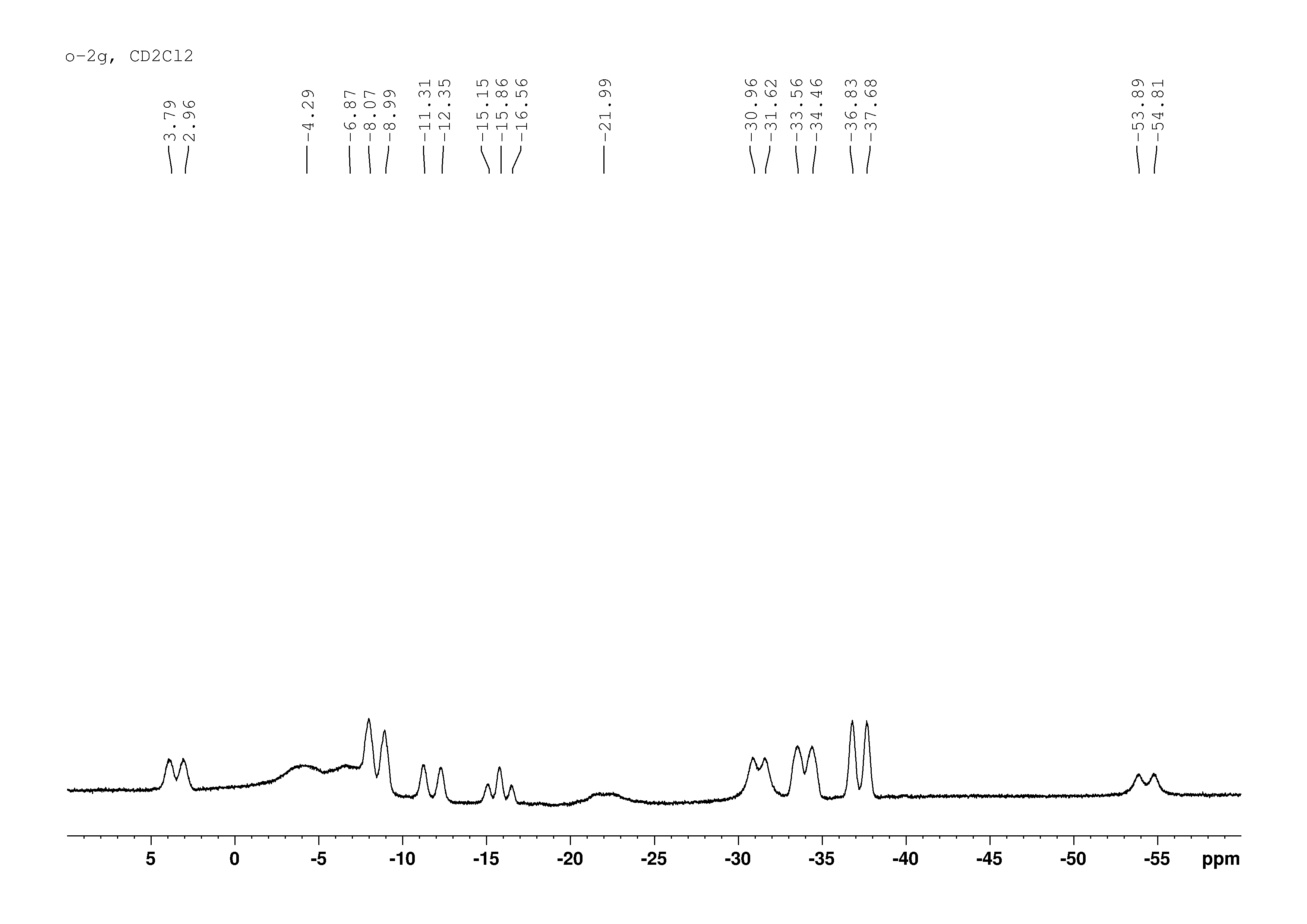


**Figure S53**. ^11^B NMR spectrum of **o-2g**.


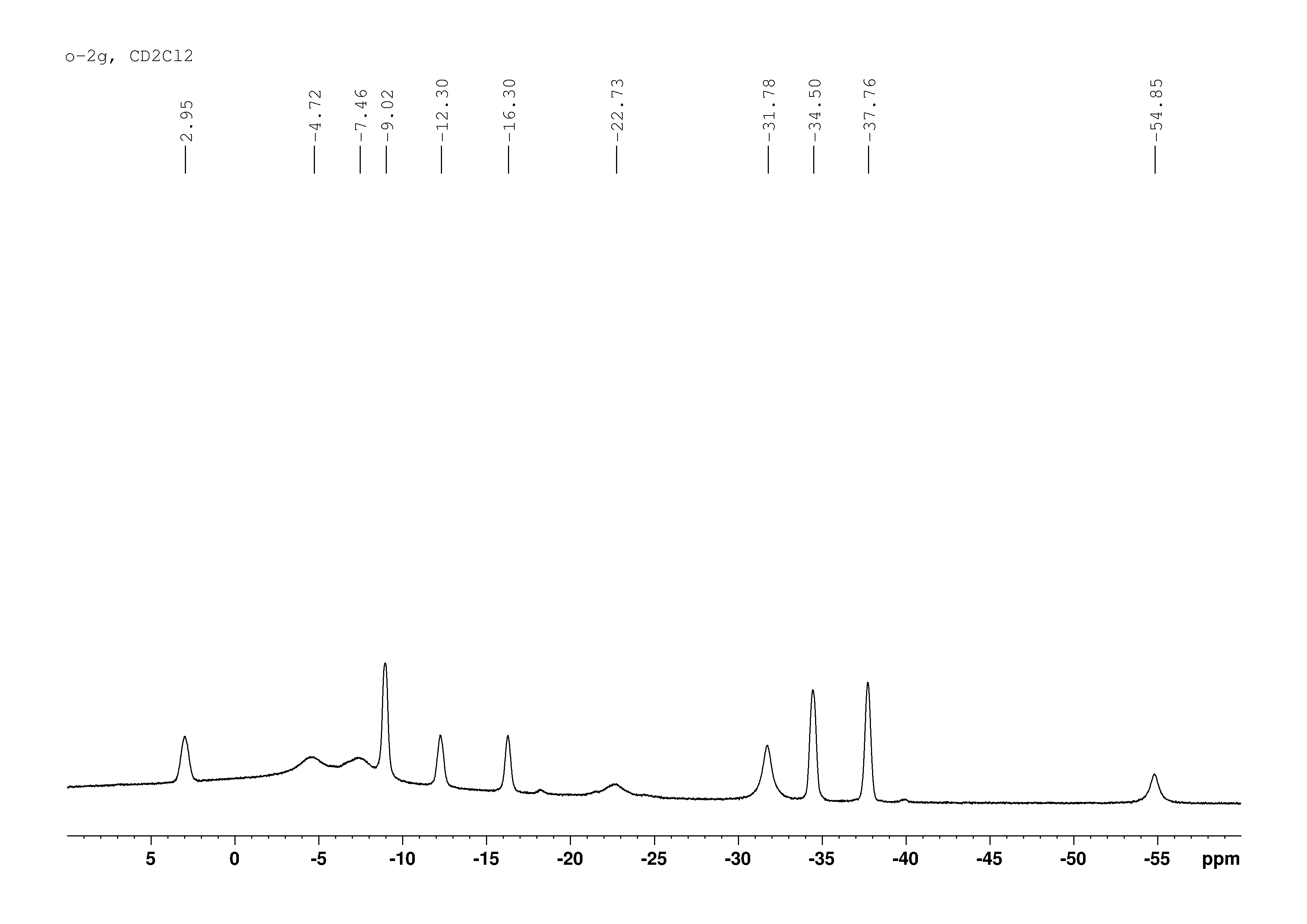


**Figure S54**. ^11^B{^1^H} NMR spectrum of **o-2g**.

**Figure S55**. Mass spectrum of negatively charged ions (ESI−, Orbitrap) for **o-2g**.

**Figure S56**. Spectrum of negatively charged ions (ESI−, Orbitrap @ R=500,000) for **o-2g** enlarged in the isotope cluster region (top) and simulated spectrum (bottom).

**Figure S57**. Spectrum of negatively charged ions (ESI−, Orbitrap @ R=500,000) for **o-2g** enlarged in the monoisotopic peak region (top) and simulated spectrum (bottom). Theoretical mass for B_9_H_12_S^−^: *m/z* 143.15027; experimental mass: *m/z* 143.15020; mass error −0.46 ppm.

**Figure S58**. Mass spectrum of positively charged ions (ESI+, Orbitrap) for **o-2g**.

**Figure S59**. Spectrum of positively charged ions (ESI+, Orbitrap @ R=500,000) for **o-2g** enlarged in the isotope cluster region (top) and simulated spectrum (bottom).

**Figure S60**. Spectrum of positively charged ions (ESI+, Orbitrap @ R=500,000) for **o-2g** enlarged in the monoisotopic peak region (top) and simulated spectrum (bottom). Theoretical mass for C_56_H_83_B_8_N_4_^+^: *m/z* 899.73567; experimental mass: *m/z* 899.73656; mass error 0.99 ppm.

**Synthesis of [6,9-I*^i^*^Pr^_2_-5,8-C_2_B_8_H_10_] (*p*-2*^i^*^Pr^)**

A solution of 1,10-C_2_B_8_H_10_ (0.35 g, 2.9 mmol) in diethyl ether (5 mL) was added dropwise to a stirred solution of **:I*^i^*^Pr^** (1.022 g, 6.7 mmol) in diethyl ether (30 mL) at room temperature. The yellowish suspension was stirred for 16 hours, filtered, and washed with diethyl ether (50 mL), yielding ***p*-2*^i-^*^Pr^** as a white powder*.* Yield 1.02 g, 83 %. **Mp.** 154 °C. **^1^H NMR** (25 °C, CD_3_CN, 500 MHz): *δ* = 0.49 (s broad, 2H, BC*H*) 1.38 (d, *^3^J*(^1^H, ^1^H) = 6.8 Hz, 24H, CH(C*H*_3_)_2_), 5.47 (sept, *^3^J*(^1^H, ^1^H) = 6.7 Hz, 4H, C*H*(CH_3_)_2_), 7.17 (s, 4H, C*H*=C*H*) ppm. **^13^C{^1^H} NMR** (25 °C, CD_3_CN, 125.76 MHz): *δ* = 22.0 (s, CH(*C*H_3_)_3_), 22.3 (s, CH(*C*H_3_)_3_), 28.6 (s, B*C*H), 48.9 (s, *C*H(CH_3_)_3_), 116.2, 117.3 (s, *C*H=*C*H), 163.4 (s very broad, N*C*N) ppm. **^11^B NMR** (25 °C, CD_3_CN, 160.46 MHz) *δ* = −44.50 (d broad, *^1^J*(^1^H,^11^B) = 145 Hz, 2B, B1,3), −36.1 (d broad, *^1^J*(^1^H,^11^B) = 115.3 Hz, 2B, B6,9), −11.89 (d broad, *^1^J*(^1^H,^11^B) = 130.4 Hz, 2B, B7,10), −7.12 (d broad, *^1^J*(^1^H,^11^B) = 130.2 Hz, 2B, B2,4) ppm.

**Spectroscopic characterization of *p*-2*^i^*^Pr^**


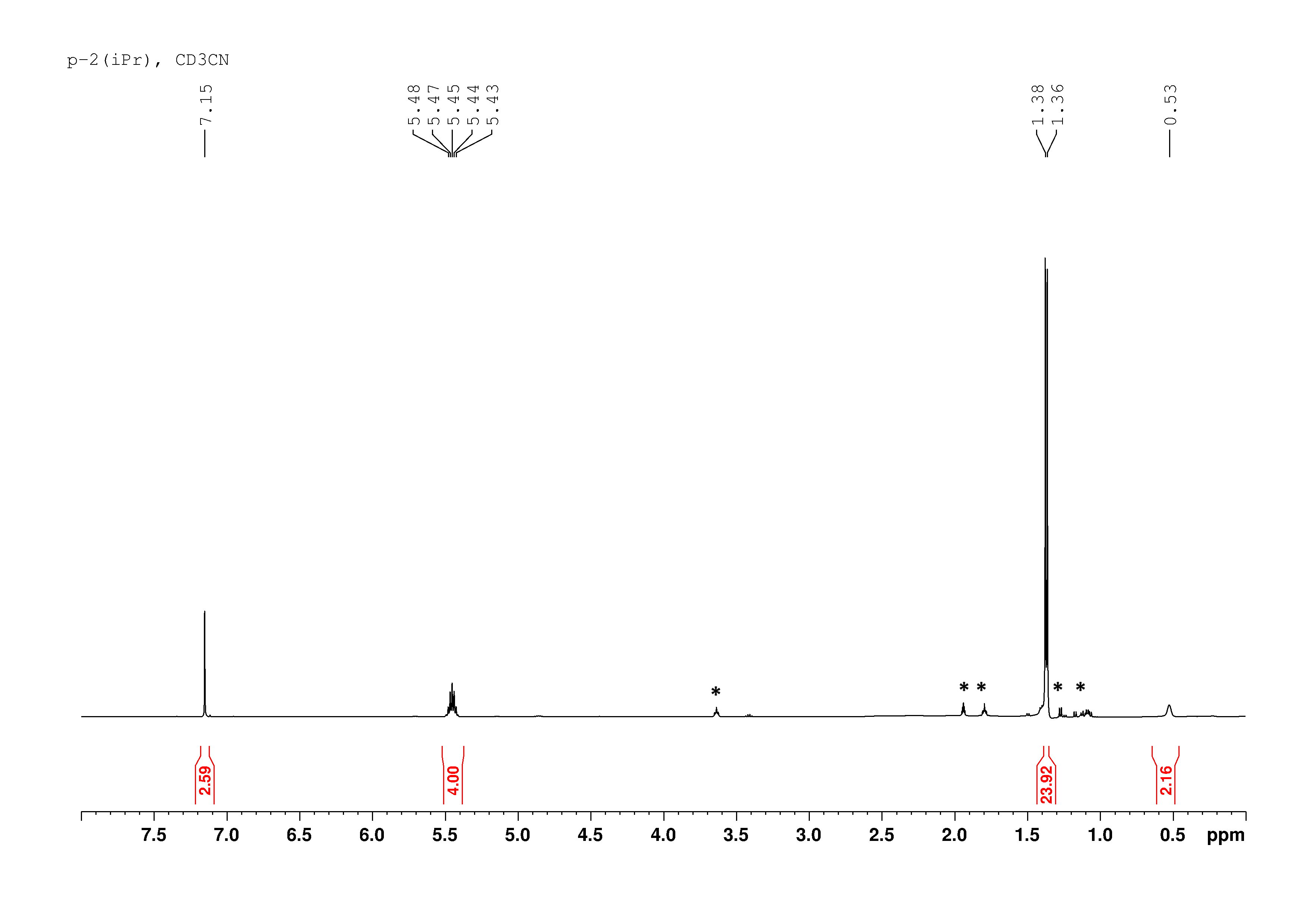


**Figure S61**. ^1^H NMR spectrum of **p-2^iPr^**. Residual THF and n-hexane are marked by *.


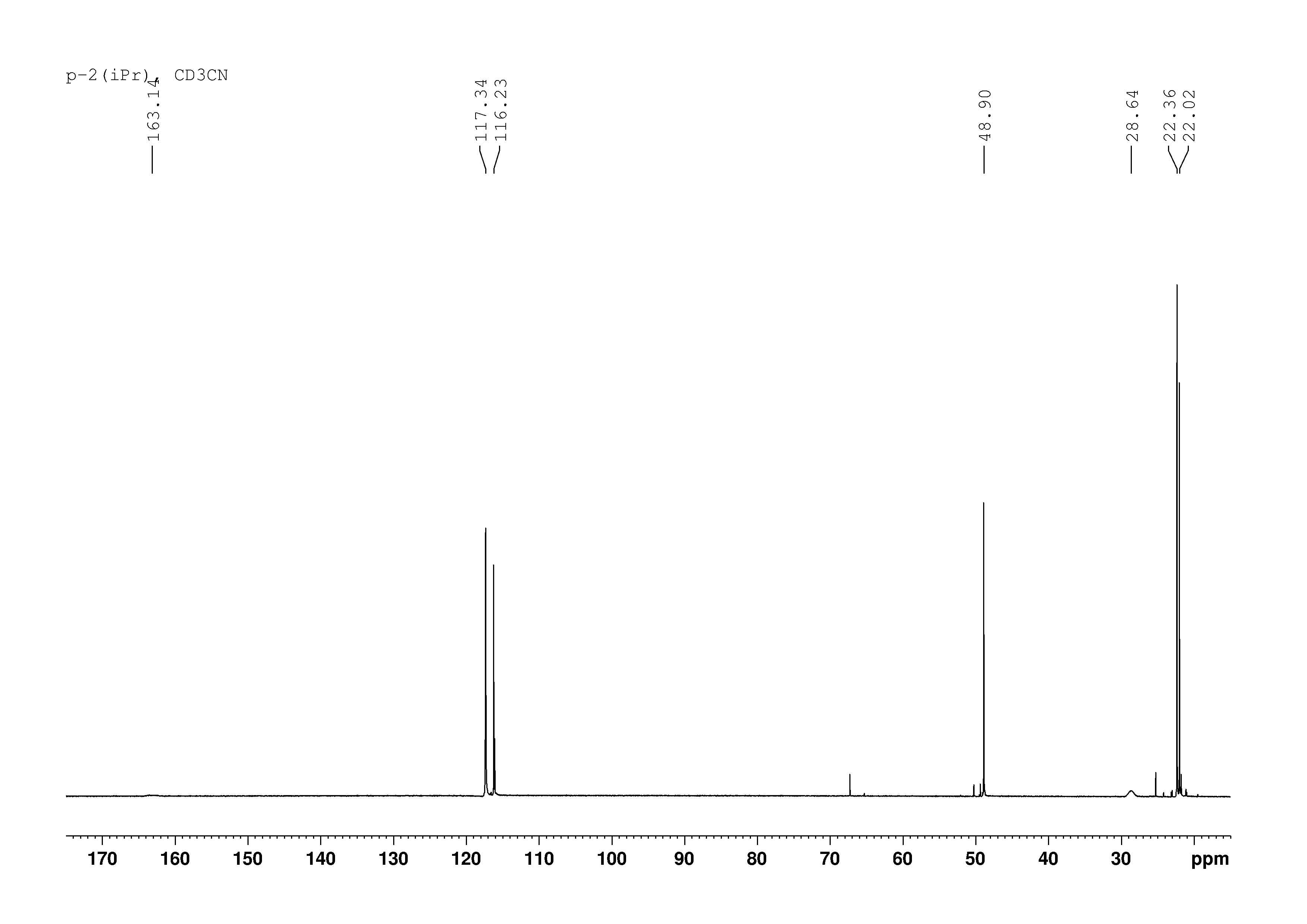


**Figure S62**. ^13^C{^1^H} NMR spectrum of **p-2^iPr^**.


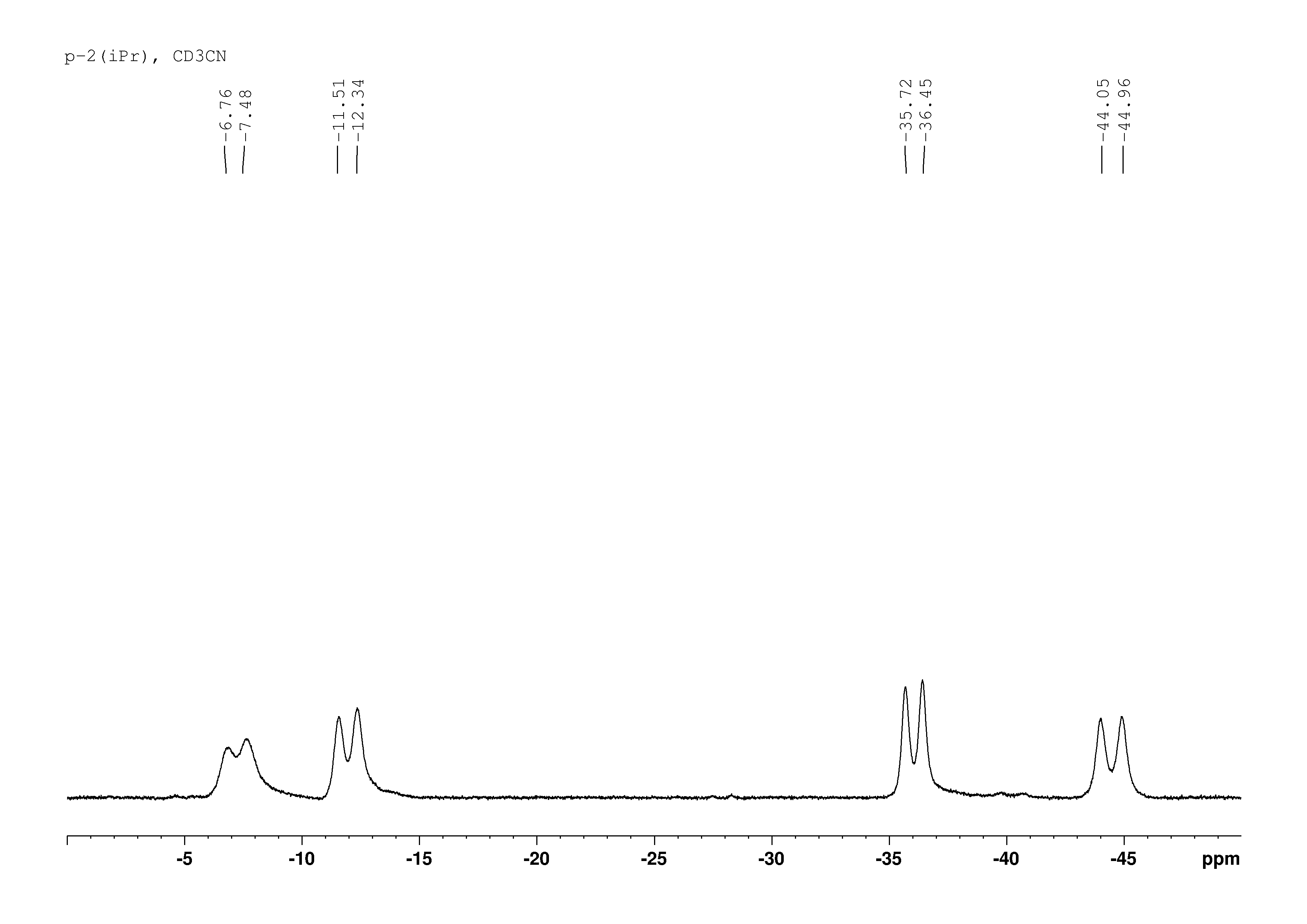


**Figure S63**. ^11^B NMR spectrum of **p-2^iPr^**.


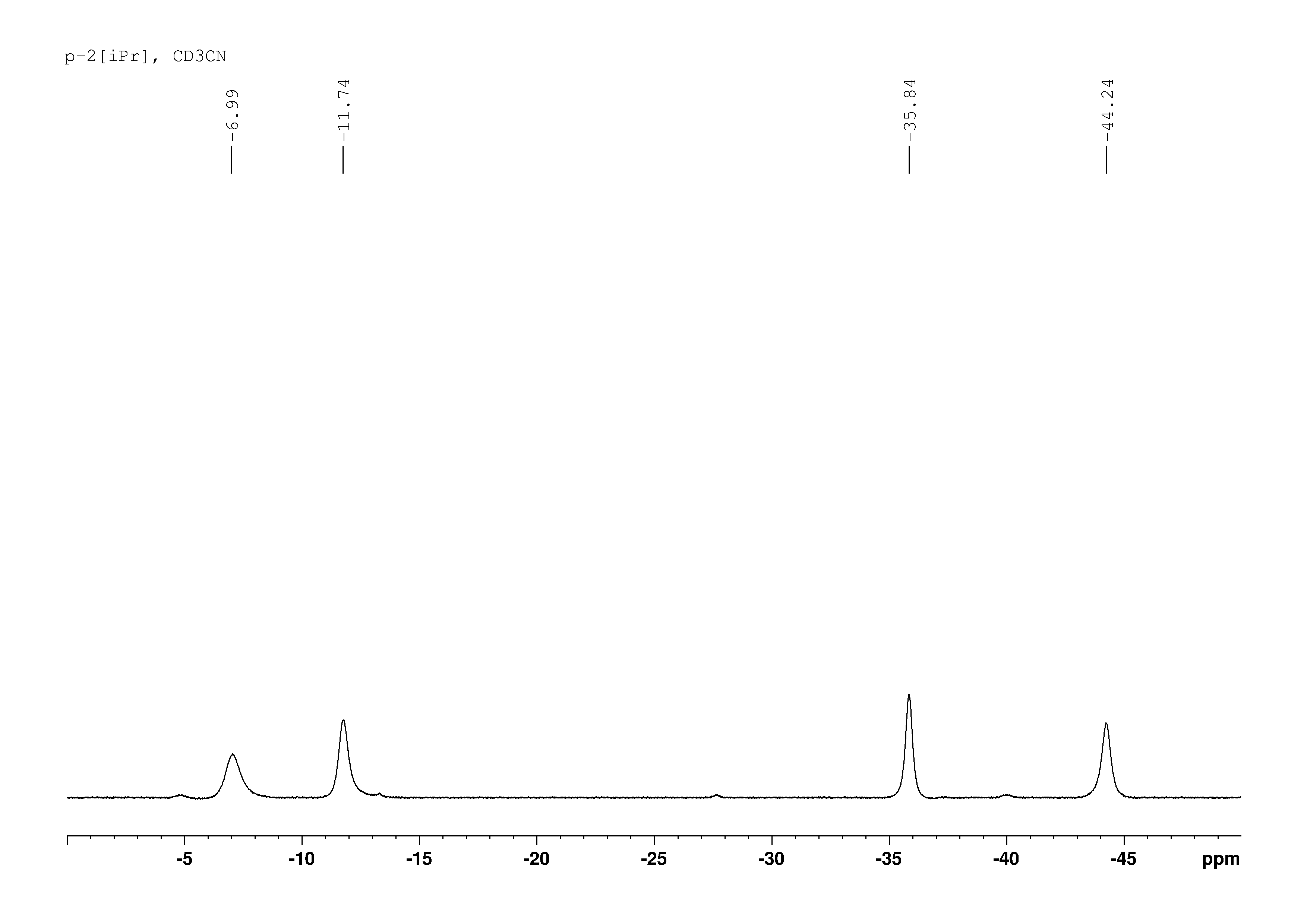


**Figure S64**. ^11^B{^1^H} NMR spectrum of **p-2^iPr^**.

**Figure S65**. Mass spectrum of negatively charged ions (ESI−, Orbitrap) for ***p*-2*^i^*^Pr^**. The spectrum shows the deprotonated molecule of ***p*-2*^iPr^*** (m/z 425.4) and ***p*-2*^iPr^*** formate adduct (*m/z* 471.4). The ion *m/z* 273.3 (C_11_H_25_N_2_B_8_^−^) is consistent with a by-products with a single **:I*^i^*^Pr^** carbene.

**Figure S66**. Spectrum of negatively charged ions (ESI−, Orbitrap @ R=500,000) for ***p*-2*^i^*^Pr^** enlarged in the deprotonated molecule region (top) and simulated spectrum (bottom).

**Figure S67**. Spectrum of negatively charged ions (ESI−, Orbitrap @ R=500,000) for ***p*-2*^i^*^Pr^** enlarged in the monoisotopic peak region (top) and simulated spectrum (bottom). Theoretical mass for C_20_B_8_H_41_N_4_^−^: *m/z* 425.40811; experimental mass: *m/z* 425.40862; mass error 1.19 ppm.

**Figure S68**. Mass spectrum of positively charged ions (ESI+, Orbitrap) for ***p*-2*^i^*^Pr^**. The spectrum shows the sodium adduct of ***p*-2*^iPr^*** (*m/z* 449.4). The ions *m/z* 273.3 (C_11_H_25_N_2_B_8_^+^) and *m/z* 579.6 (C_29_H_59_N_6_B_8_^+^) are consistent with by-products with one and three **:I*^i^*^Pr^** carbenes.

**Figure S69**. Spectrum of positively charged ions (ESI+, Orbitrap @ R=500,000) for ***p*-2*^i^*^Pr^** enlarged in the sodium adduct region (top) and simulated spectrum (bottom).

**Figure S70**. Spectrum of positively charged ions (ESI+, Orbitrap @ R=500,000) for ***p*-2*^i^*^Pr^** enlarged in the monoisotopic peak region (top) and simulated spectrum (bottom). Theoretical mass for C_20_H_42_B_8_N_4_Na^+^: *m/z* 449.40461; experimental mass: *m/z* 449.40479; mass error 0.40 ppm.

**Synthesis of [6,9-I^Cy^_2_-5,8-C_2_B_8_H_10_] (*p*-2^Cy^)**

A solution of 1,10-C_2_B_8_H_10_ (0.162 g, 1.3 mmol) in tetrahydrofuran (5 mL) was added to the stirred solution of **:I^Cy^** in tetrahydrofuran (5 mL). The resulting yellowish solution was stirred for 16 hours. The volatiles were removed in *vacuo* and washed with acetonitrile (50 mL) yielding ***p*-2^Cy^** as a white powder. Yield 0.724 g, 60 %. **Mp**. 178 °C. **^1^H NMR** (25 °C, THF-D_8_, 500 MHz): *δ* = 0.60 (s broad, 2H, BC*H*), 1.24−1.31 (m, 4H, C_6_*H*_12_), 1.51−1.63 (m, 15H, C_6_*H*_12_), 1.74−1.77 (m, 6H, C_6_*H*_12_), 1.87 (d broad, 8H, C_6_*H*_12_), 2.00−2.07 (m, 8H, C_6_*H*_12_), 5.30 (broad s, 4H, C_6_*H*_12_), 7.20 (s, 4H, CH=CH) ppm. **^13^C{^1^H} NMR** (25 °C, THF-D_8_, 125.76 MHz): *δ* = 25.4, 25.7, 25.8 (s, *C_6_*H_12_), 28.5 (s, B*C*H), 33.4, 33.5, 55.9 (s, *C_6_*H_12_), 116.0 (s, *C*H=*C*H), 165.7, 166.4 (s very broad, N*C*N) ppm. **^11^B NMR** (25 °C, THF-D_8_, 160.46 MHz) *δ* = −44.4 (d broad, *^1^J*(^1^H, ^11^B) = 138.50 Hz, 2B, B1,3), −36.5 (d broad, *^1^J*(^1^H,^11^B) = 115.1 Hz, 2B, B6,9), −11.4 (d broad, *^1^J*(^1^H, ^11^B) = 110.3 Hz, 2B, B7,10), −6.6 (s broad, 2B, B2,4) ppm.

**Spectroscopic characterization of *p*-2^Cy^**


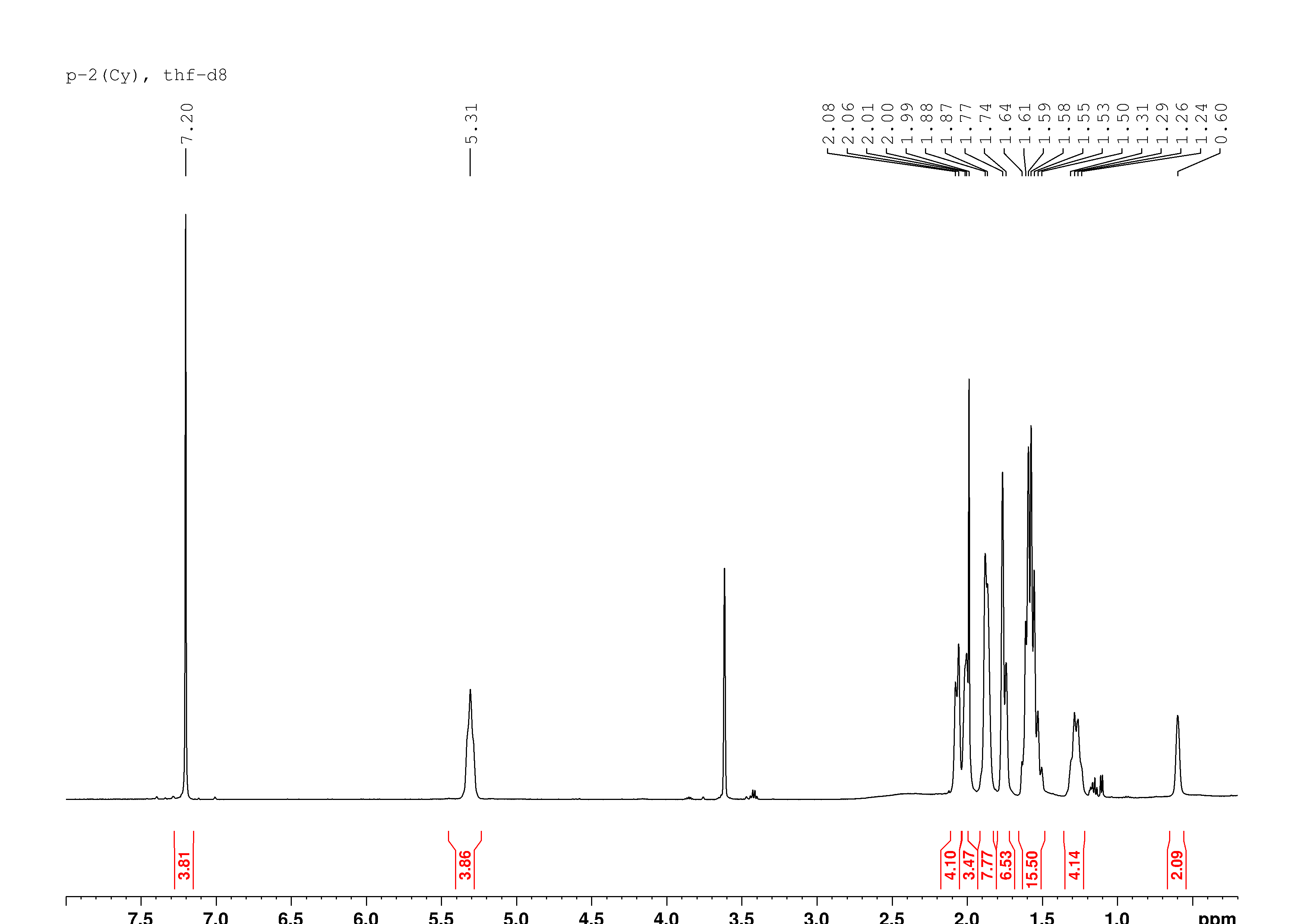


**Figure S71**. ^1^H NMR spectrum of **p-2^Cy^**.


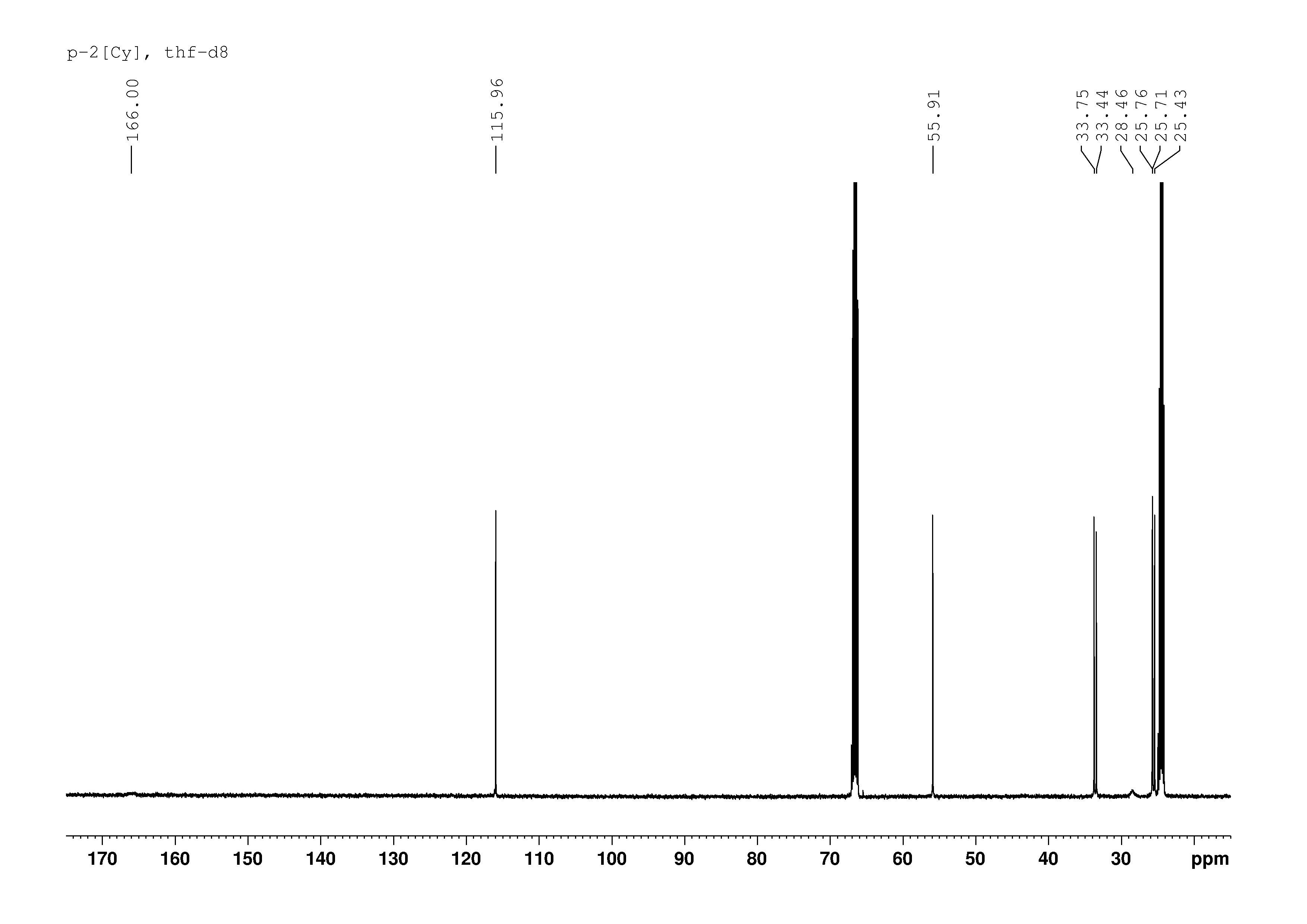


**Figure S72**. ^13^C{^1^H} NMR spectrum of **p-2^Cy^**.


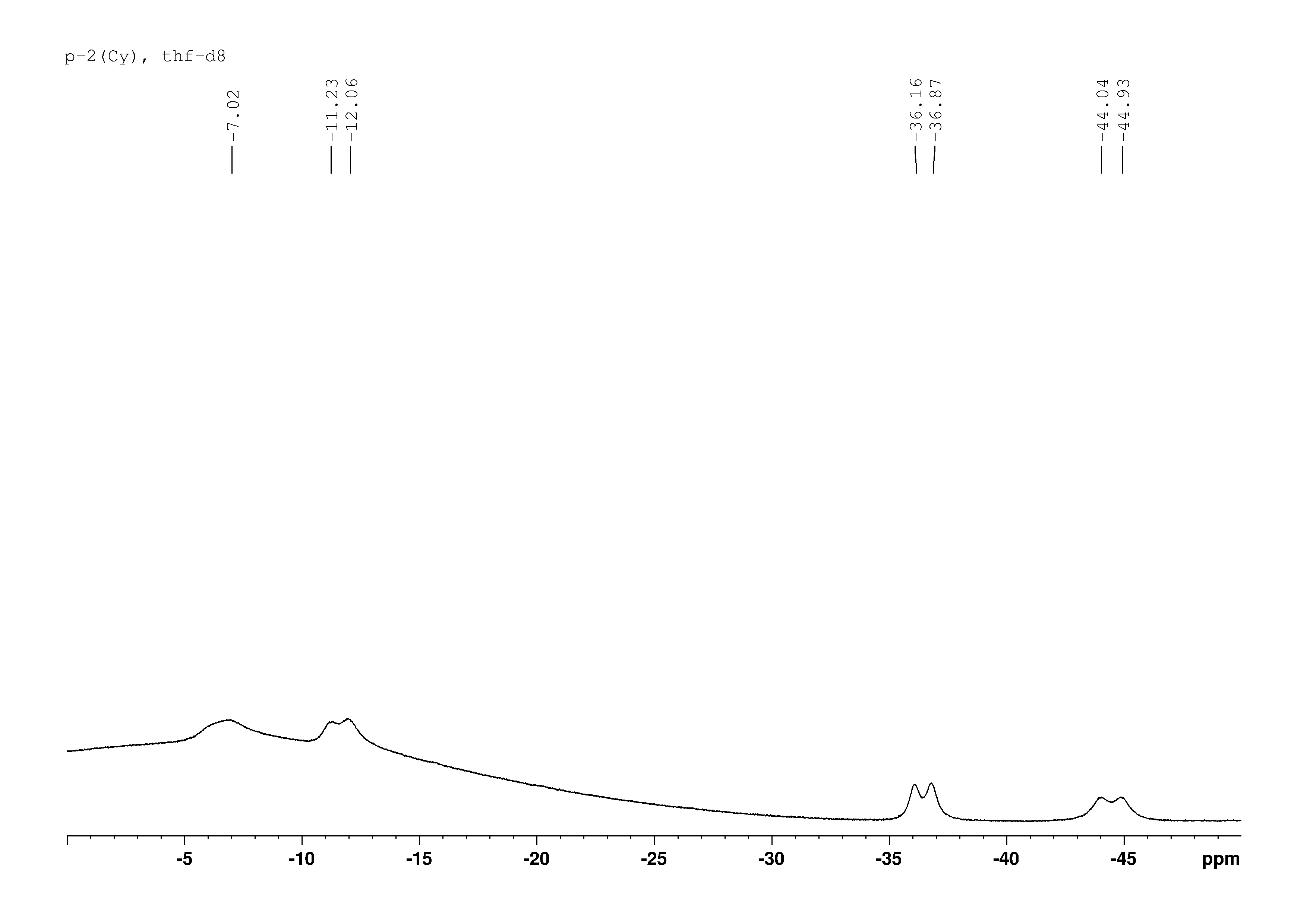


**Figure S73**. ^11^B NMR spectrum of **p-2^Cy^**.


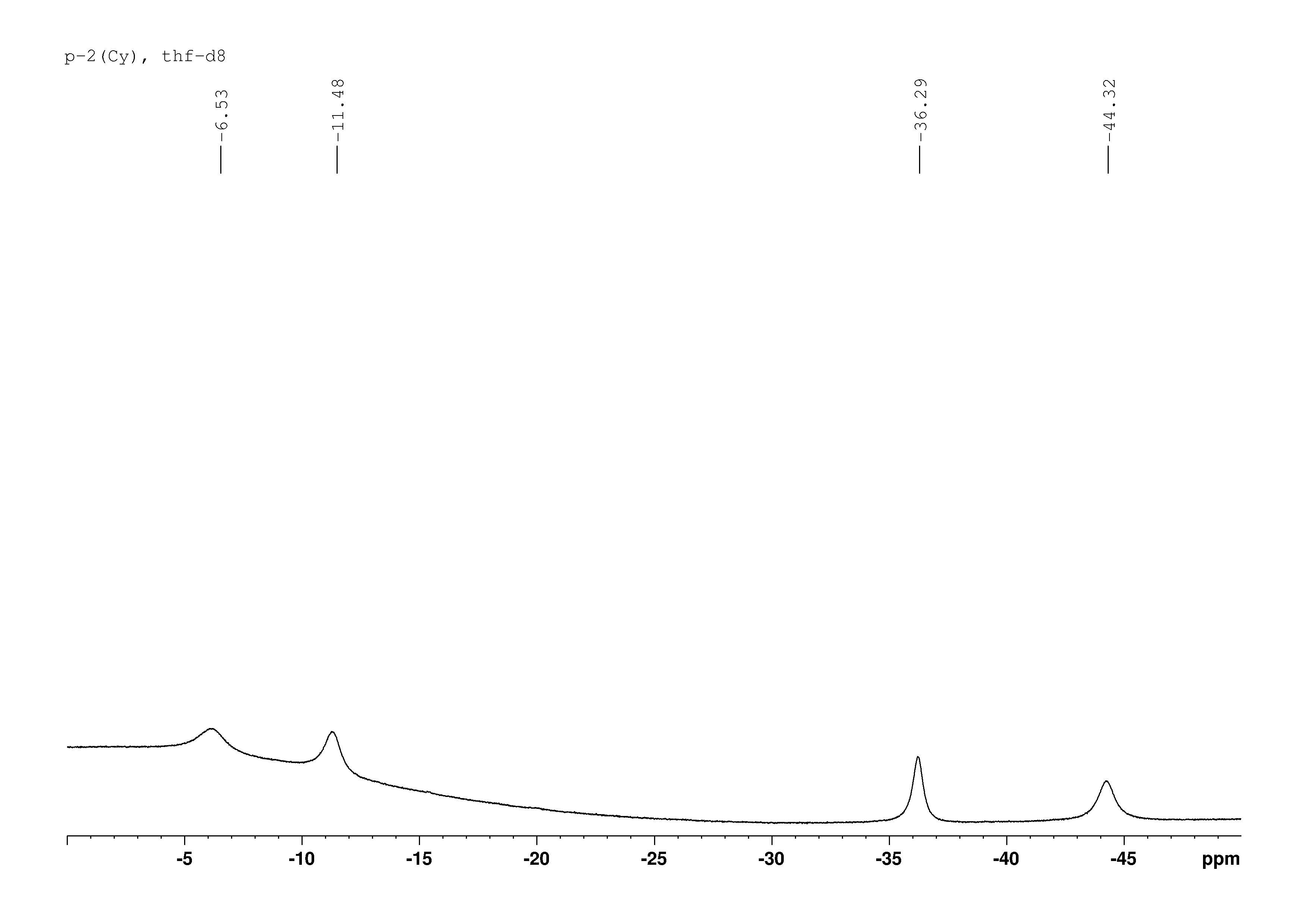


**Figure S74**. ^11^B{^1^H} NMR spectrum of **p-2^Cy^**.

**Figure S75**. Mass spectrum of positively charged ions (ESI+, Orbitrap) for ***p*-2^Cy^**. The spectrum shows the sodium adduct of ***p*-2*^Cy^*** (*m/z* 609.5). The ion *m/z* 353.3 (C_17_H_33_N_2_B_8_^+^) is consistent with a by-product with one **:I*^i^*^Pr^**; the isotope profile also indicates an overlap with an unknown impurity.

**Figure S76**. Spectrum of positively charged ions (ESI+, Orbitrap @ R=500,000) for ***p*-2^Cy^** enlarged in the sodium adduct region (top) and simulated spectrum (bottom).

**Figure S77**. Spectrum of positively charged ions (ESI+, Orbitrap @ R=500,000) for ***p*-2^Cy^** enlarged in the monoisotopic peak region (top) and simulated spectrum (bottom). Theoretical mass for C_32_H_58_B_8_N_4_Na^+^: *m/z* 609.52981; experimental mass: *m/z* 609.53006; mass error 0.41 ppm.

**Synthesis of [2,6-I*^i^*^Pr^_2_-1,10-C_2_B_8_H_8_]Cl_2_ (*p*-2a*^i^*^Pr^)**

Hydrochloric acid (9.7 mL, 9.73 mmol, 1M solution in diethyl ether) was added to the suspension of ***p*-2*^i^*^Pr^** (1.03 g, 2.43 mmol) in tetrahydrofuran (25 mL) at −78 °C. The resulting reaction mixture was gradually warmed to room temperature and stirred for 16 hours. The suspension was filtered and the solid was washed with dichloromethane (30 mL) and acetonitrile (30 mL)*,* yielding ***p*-2a*^i^*^Pr^** as a white powder*.* Yield 0.724 g, 60 %. **Mp.** 300 °C. **^1^H NMR** (25 °C, CD_3_OD, 500 MHz): *δ* = 0.62 (s, 6H, CH(C*H*_3_)_2_), 1.35 (s broad, 18H, CH(C*H*_3_)_2_), 3.53 (s broad, 2H, C*H*(CH_3_)_2_), 4.52 (s broad, 2H, C*H*(CH_3_)_2_), 7.87 (s broad, 2H, C*H*=C*H*), 8.00 (s, 2H, C*H*=C*H*), 8.26 (s, 2H, BC*H*) ppm. **^13^C{^1^H} NMR** (25 °C, CD_3_OD, 125.76 MHz): *δ* = 21.6, 23.0, 24.1, 24.5 (s, CH(*C*H_3_)_3_), 54.2 (s, *C*H(CH_3_)_3_), 107.5 (s B*C*H), 124.5, 125.3 (s, *C*H=*C*H), 137.9 (s very broad, N*C*N) ppm. **^11^B NMR** (25 °C, CD_3_OD, 160.46 MHz) *δ* = −12.0 (s broad, 2B, B6,9 ), −9.7 (m broad) ppm.

**Spectroscopic characterization of *p*-2a*^i^*^Pr^**


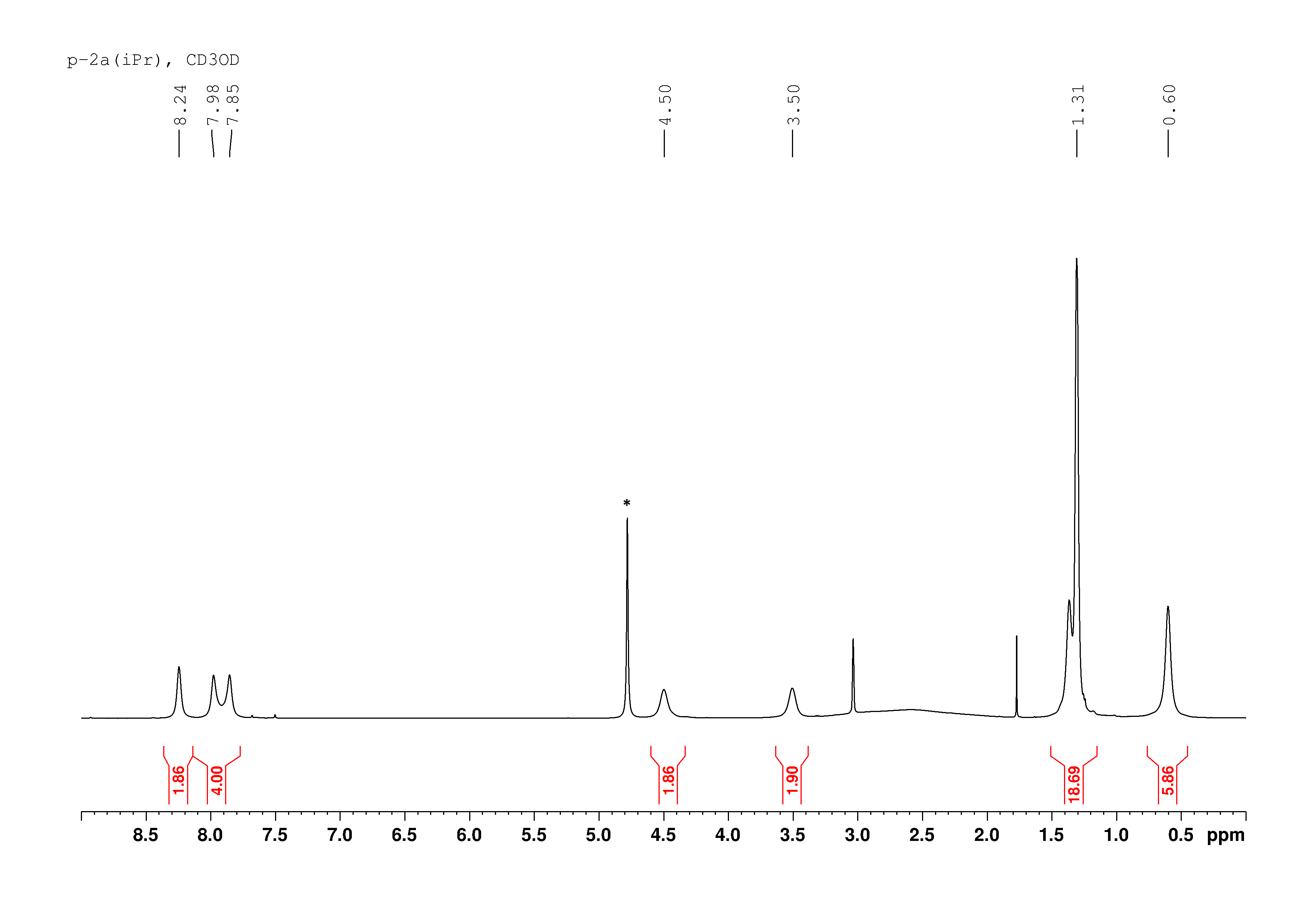


**Figure S78**. ^1^H NMR spectrum of **p-2a^iPr^**. Residual water is marked by *.


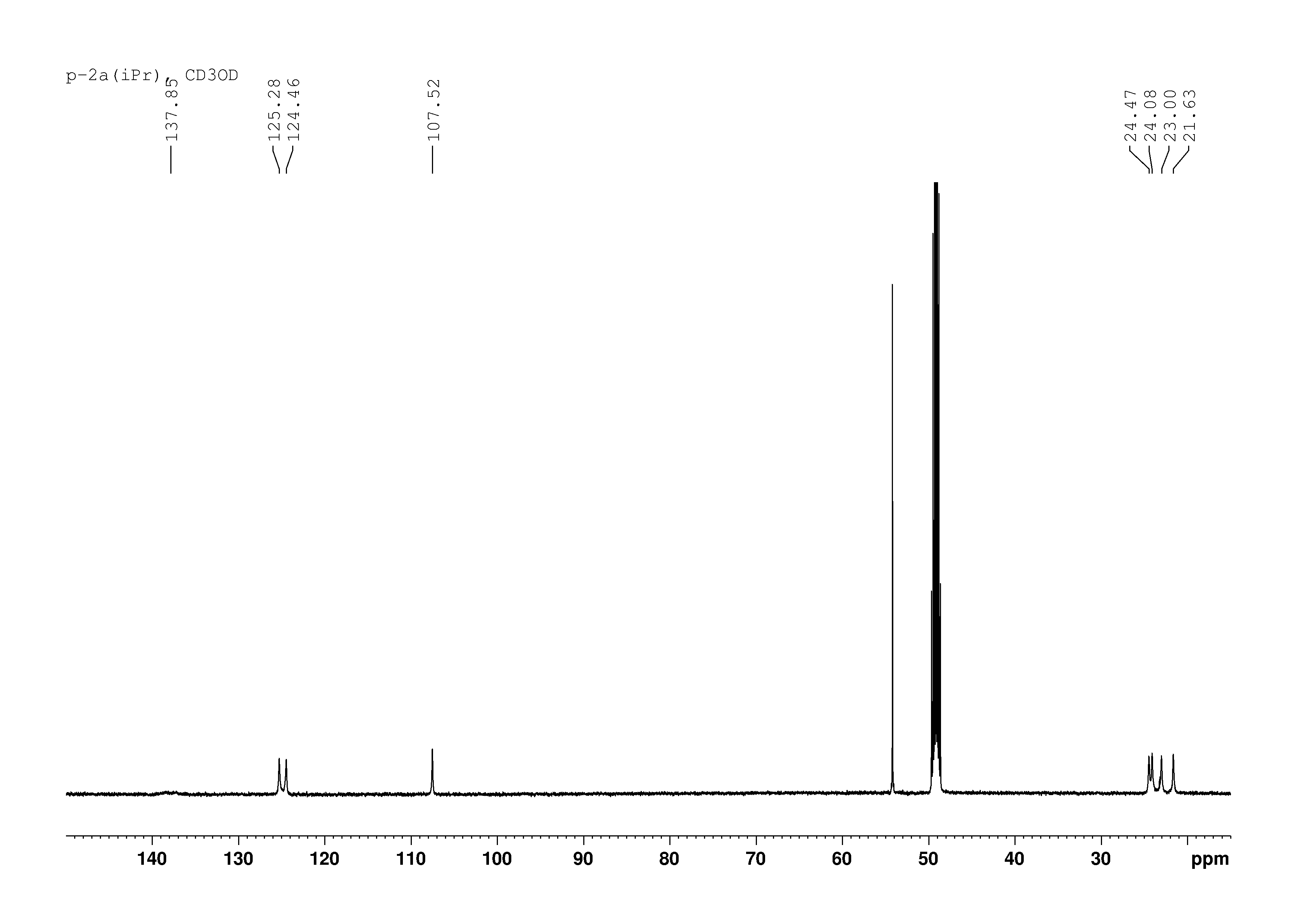


**Figure S79**. ^13^C{^1^H} NMR spectrum of **p-2a^iPr^**. Residual dichloromethane is marked by *.


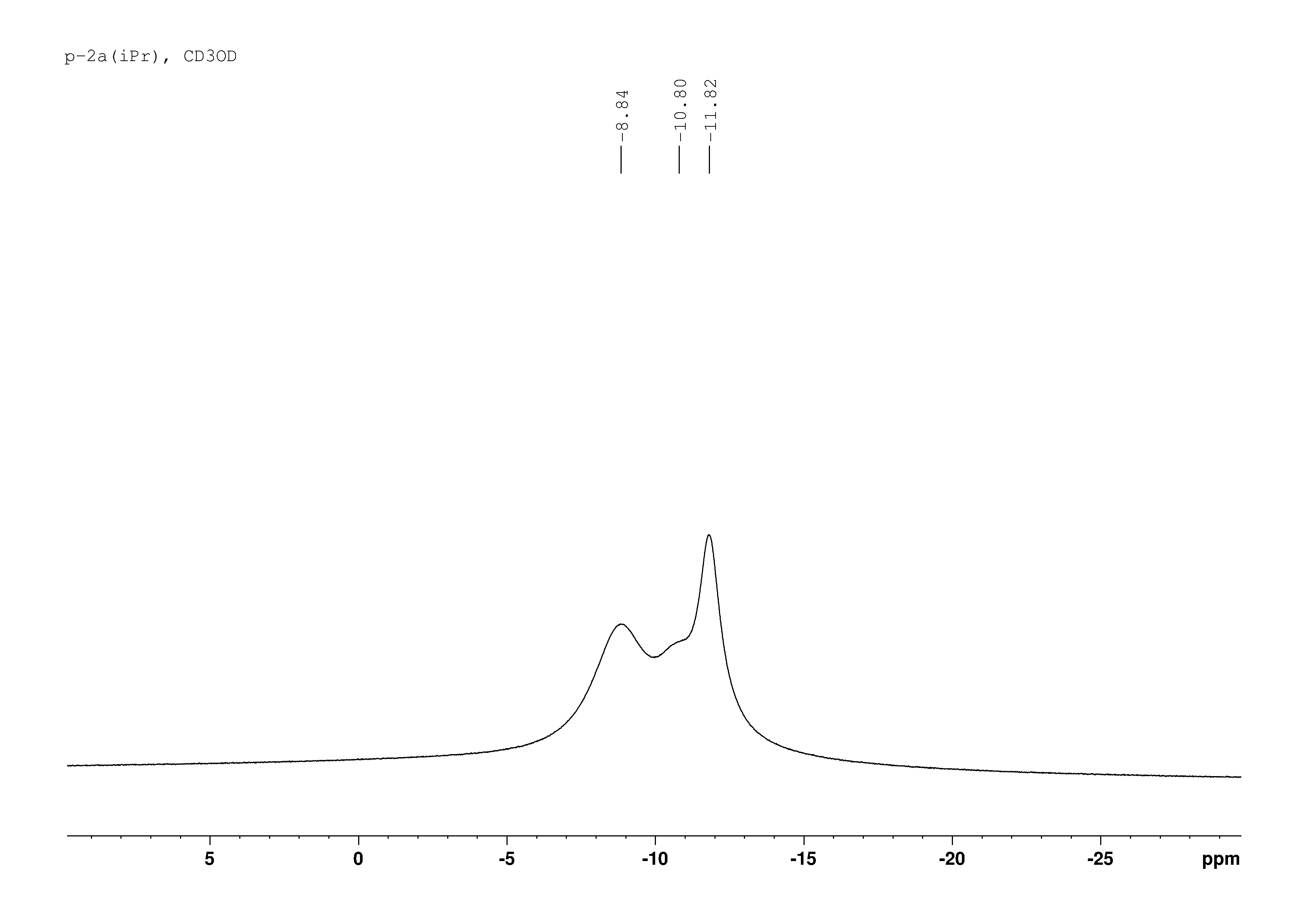


**Figure S80**. ^11^B NMR spectrum of **p-2a^iPr^**.


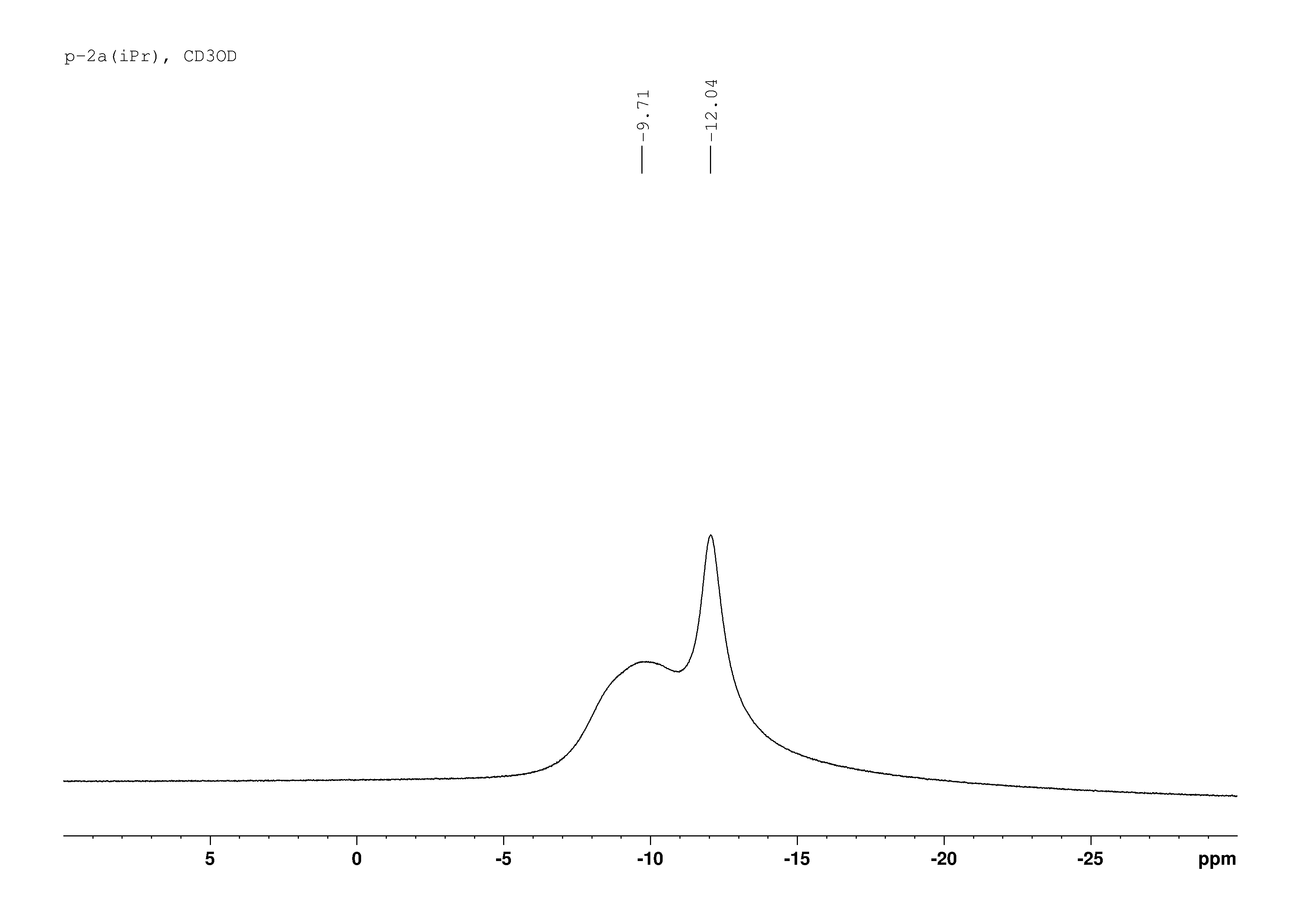


**Figure S81**. ^11^B{^1^H} NMR spectrum of **p-2a^iPr^**.

**Figure S82**. Mass spectrum of positively charged ions (ESI+, Orbitrap) for ***p*-2a*^i^*^Pr^**. The spectrum shows a doubly charged ion of ***p*-2a*^iPr^*** (*m/z* 212.2). The ion *m/z* 373.3 (C_11_H_25_N_2_B_8_^+^) is consistent with a by-product with one **:I*^i^*^Pr^**.

**Figure S83**. Spectrum of positively charged ions (ESI+, Orbitrap @ R=500,000) for ***p*-2a*^i^*^Pr^** enlarged in the isotope cluster region (top) and simulated spectrum (bottom).

**Figure S84**. Spectrum of positively charged ions (ESI+, Orbitrap @ R=500,000) for ***p*-2a*^i^*^Pr^** enlarged in the monoisotopic peak region (top) and simulated spectrum (bottom). Theoretical mass for C_20_H_40_B_8_N_4_^2+^: *m/z* 212.19932; experimental mass: *m/z* 212.19937; mass error 0.24 ppm.

**Synthesis of [2,6-I^Cy^_2_-1,10-C_2_B_8_H_8_]Cl_2_ (*p*-2a^Cy^)**

Hydrochloric acid (4 mL, 4.0 mmol, 1M solution in diethyl ether) was added to the suspension of ***p*-2^Cy^** (0.59 g, 1.0 mmol) in tetrahydrofuran (25 mL) at −78 °C. The resulting reaction mixture was gradually warmed to room temperature and stirred for 16 hours. The suspension was filtered and the solid was washed with dichloromethane (30 mL) and acetonitrile (30 mL). The solid was dried in *vacuo,* yielding ***p*-2a^Cy^** as a white powder*.* Yield 0.32 g, 49 %. **Mp.** 246 °C. **^1^H NMR** (25 °C, CD_3_OD, 500 MHz): *δ* = 0.68−0.76 (m, 2H, C_6_*H*_12_), 0.94−0.97 (m, 3H, C_6_*H*_12_), 1.21−2.26 (m, 34 H, C_6_*H*_12_), 3.07 (m broad, 2H, C_6_*H*_12_), 4.40 (m broad, 2H, C_6_*H*_12_), 8.14 (s, 2H, C*H*=C*H*), 8.31 (s, 2H, C*H*=C*H*), 8.53 (s broad, 2H, BC*H*) ppm. **^13^C{^1^H} NMR** (25 °C, CD_3_OD, 125.76 MHz): *δ* = 23.6, 24.1, 24.6, 24.9, 25.0, 30.7, 32.3, 33.1, 34.8, 35.1 (s, *C*_6_H_12_), 59.7 (s, *C*_6_H_12_), 60.2 (s, *C*_6_H_12_), 106.9 (s B*C*H), 123.4, 124.2 (s, *C*H=*C*H), 136.8, 137.7 (s very broad, N*C*N) ppm. **^11^B NMR** (25 °C, CD_3_OD, 160.46 MHz) *δ* = −12.2 (m broad), −9.8 (m broad).

**Spectroscopic characterization of *p*-2a^Cy^**


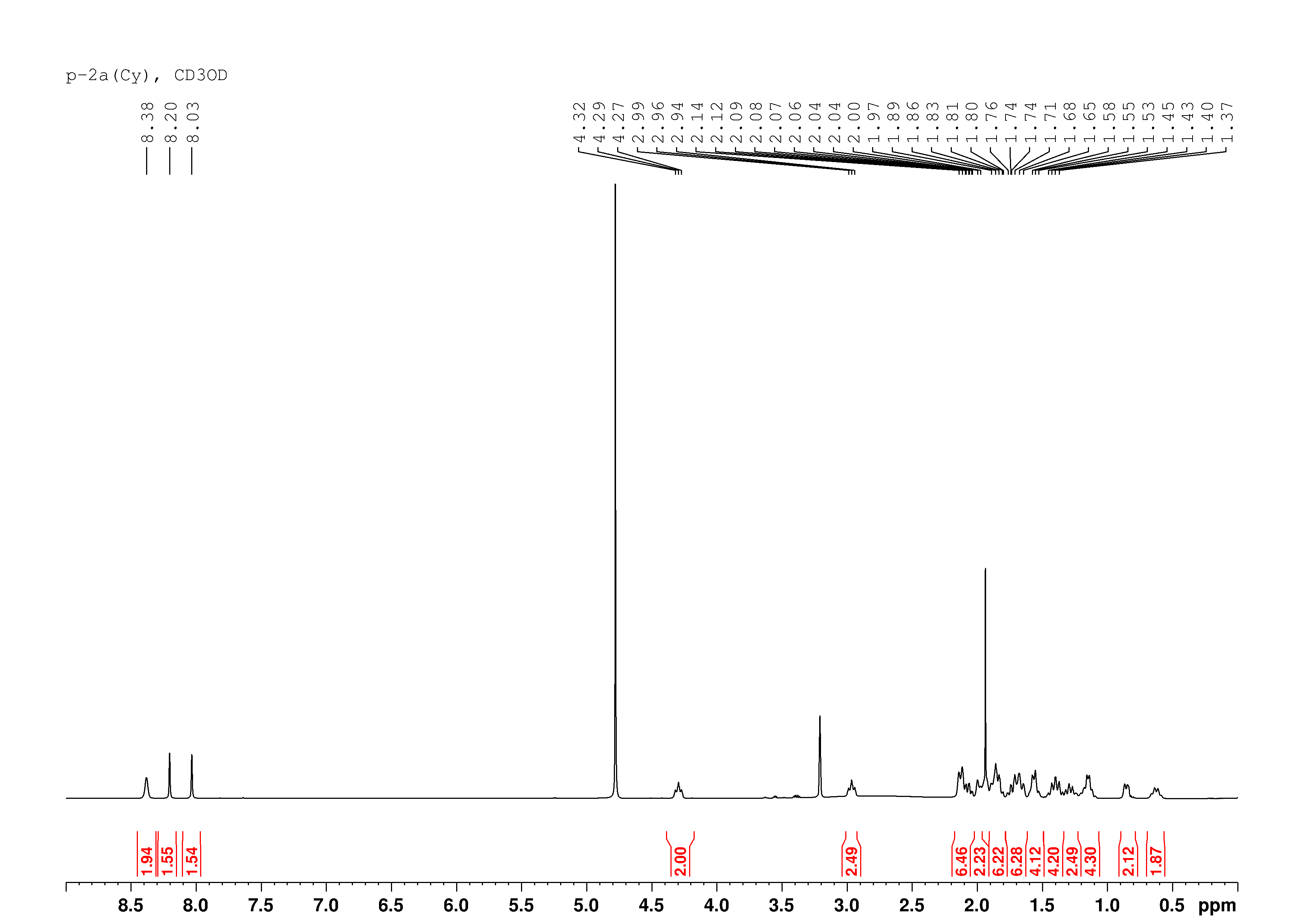


**Figure S85**. ^1^H NMR spectrum of **p-2a^Cy^**. Residual water is marked by *.


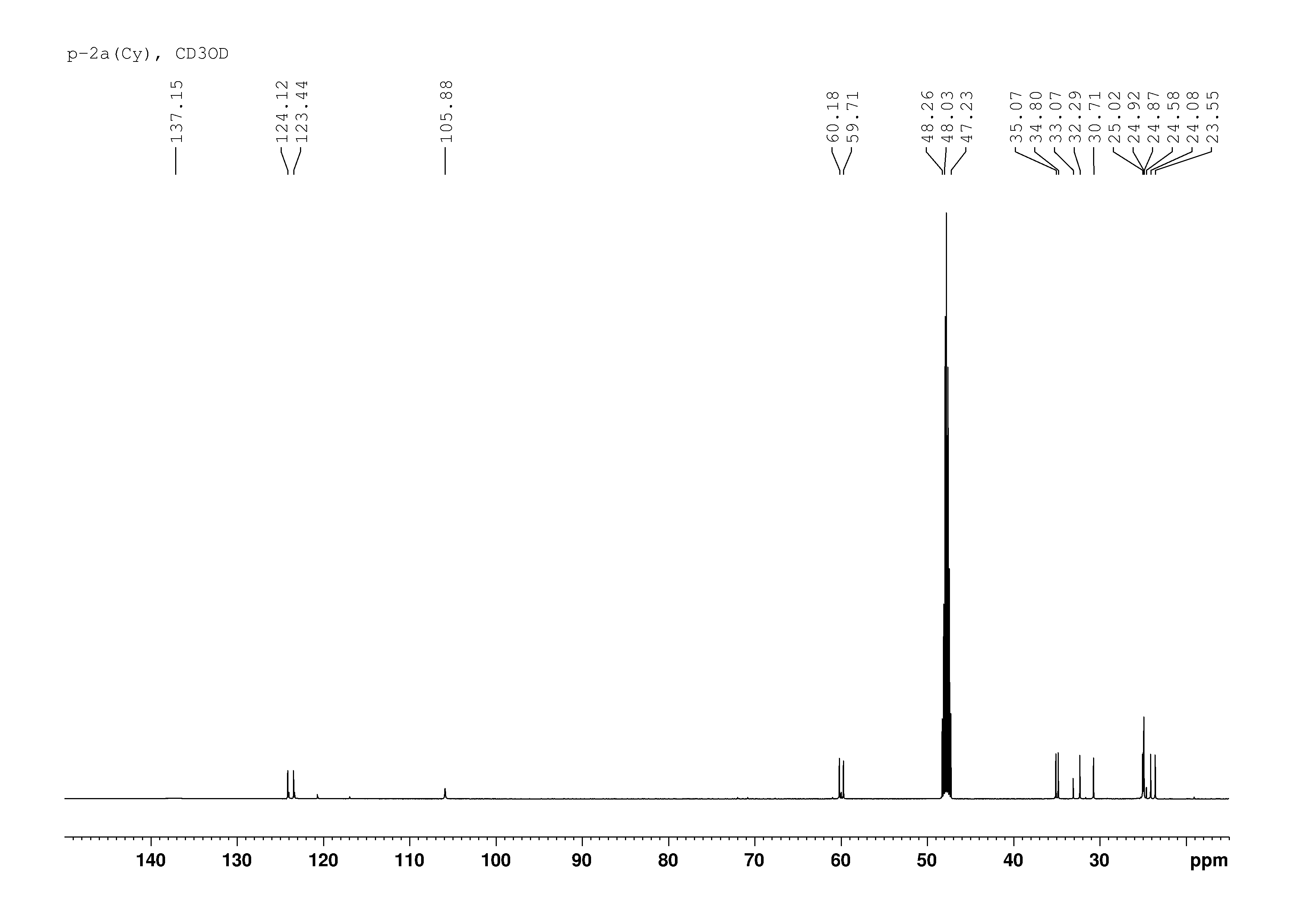


**Figure S86**. ^13^C{^1^H} NMR spectrum of **p-2a^Cy^**. Residual dichloromethane is marked by *.


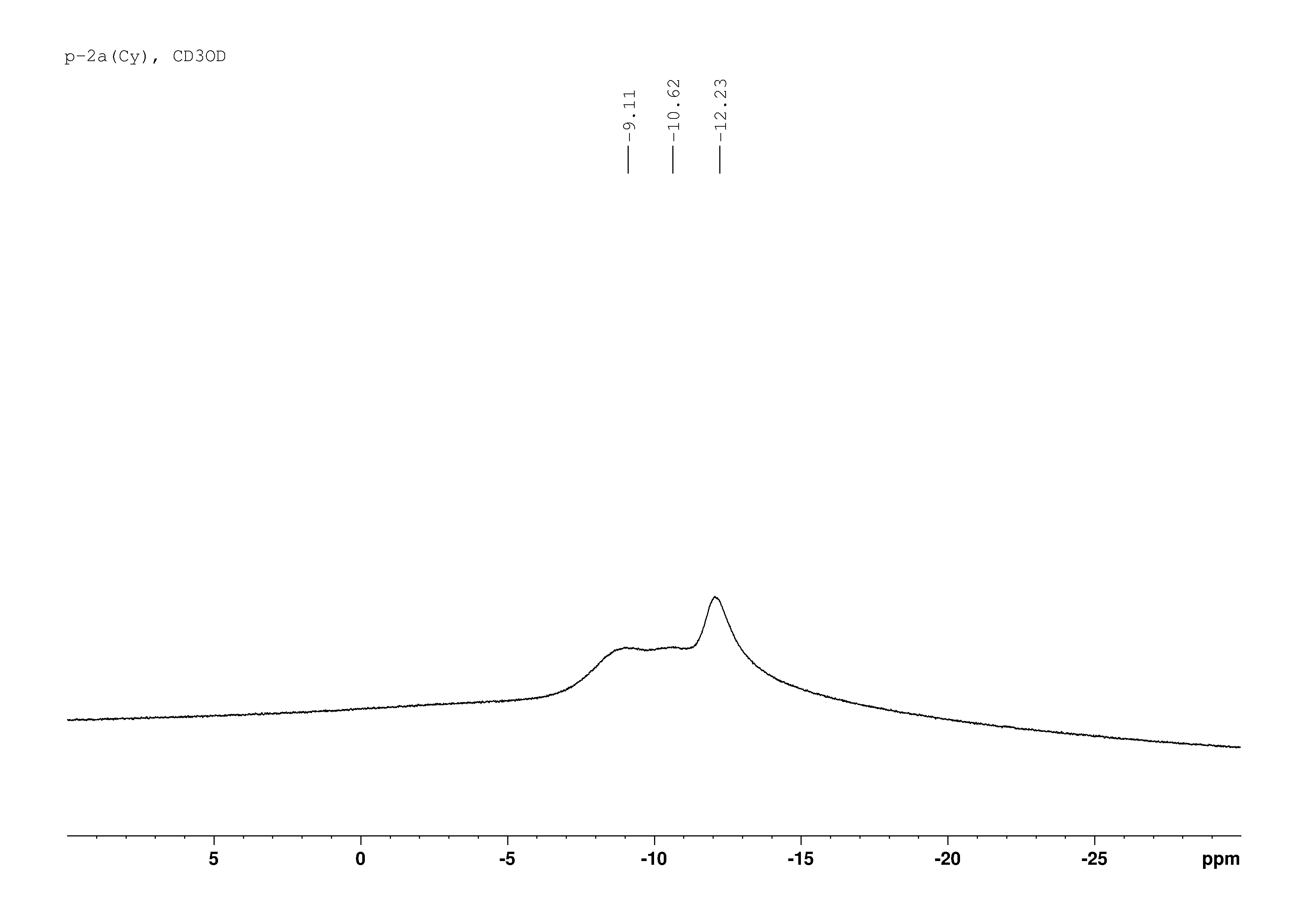


**Figure S87**. ^11^B NMR spectrum of **p-2a^Cy^**.


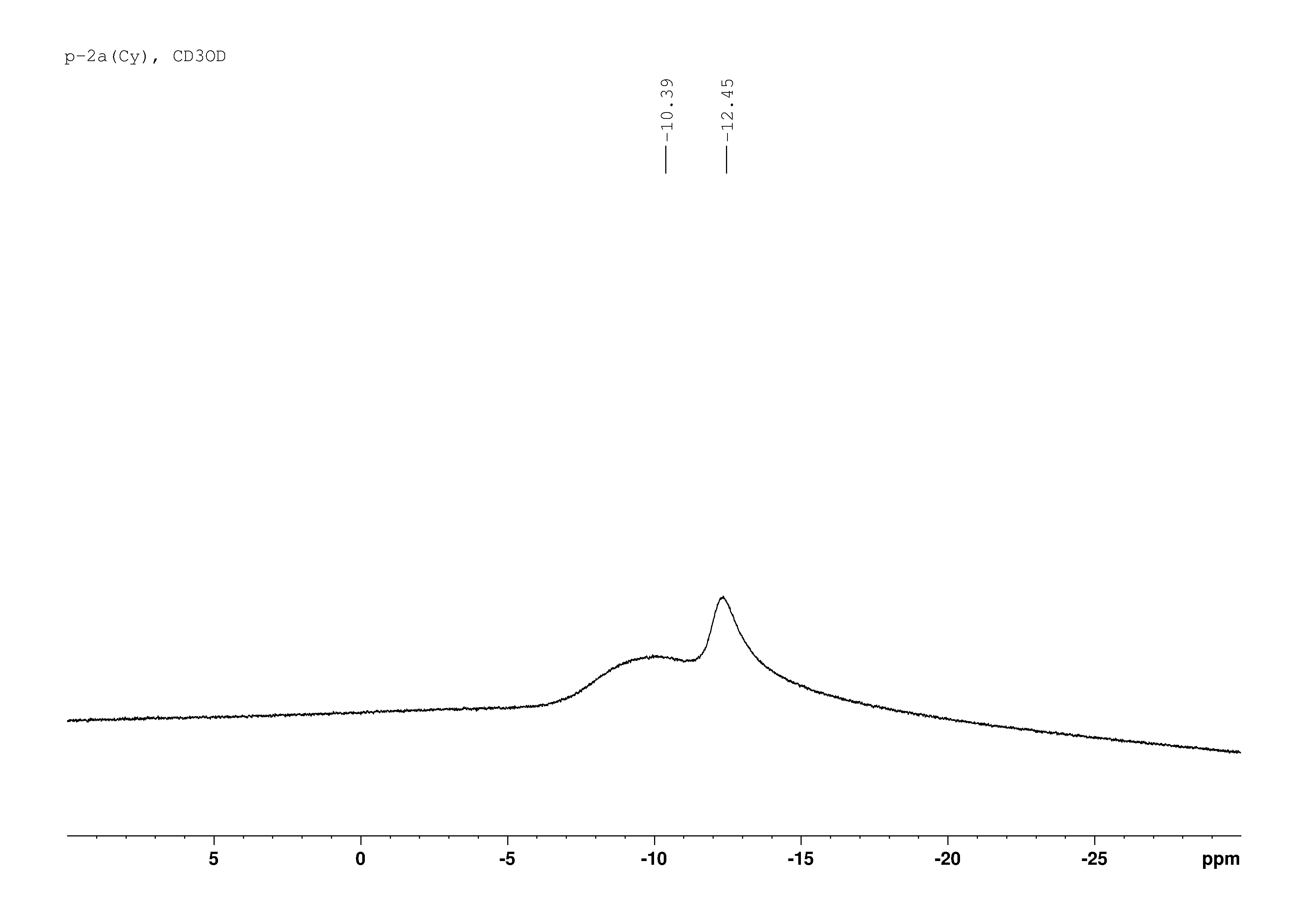


**Figure S88**. ^11^B{^1^H} NMR spectrum of **p-2a^Cy^**.

**Figure S89**. Mass spectrum of positively charged ions (ESI+, Orbitrap) for ***p*-2a^Cy^**. The spectrum shows a doubly charged ion of ***p*-2a*^Cy^*** (*m/z* 292.3). The ion *m/z* 233.2 (C_15_H_25_N_2_^+^) is a protonated **:I^Cy^**.

**Figure S90**. Spectrum of positively charged ions (ESI+, Orbitrap @ R=500,000) for ***p*-2a^Cy^** enlarged in the isotope cluster region (top) and simulated spectrum (bottom).

**Figure S91**. Spectrum of positively charged ions (ESI+, Orbitrap @ R=500,000) for ***p*-2a^Cy^** enlarged in the monoisotopic peak region (top) and simulated spectrum (bottom). Theoretical mass for C_32_H_56_B_8_N_4_^2+^: *m/z* 292.26192; experimental mass: *m/z* 292.26200; mass error 0.26 ppm.

**Synthesis of 9,10-I*^i^*^Pr^_2_-6,9-(CN)_2_-5,8-C_2_B_8_H_8_ (*p*-2b*^i^*^Pr^)**

**Path A**

A solution of potassium cyanide (0.02 g, 0.3 mmol) in methanol (2 mL) was added dropwise to a solution of ***p*-2a*^i^*^Pr^** (0.06 g, 0.12 mmol) in methanol (5 mL) at –78 °C and stirred for 16 hours. The volatiles were removed *in vacuo* and the solid was extracted with mixture of dichloromethane and hexane (2:1) and left for 7 days for crystallization. The colorless crystals were decanted and dried *in vacuo.* Yield 0.02 g, 35 %.

**Path B**

Solution of tetrabutylammonium cyanide (0.08 g, 0.3 mmol) in acetonitrile (2 mL) was added dropwise to a solution of ***p*-2c*^i^*^Pr^** (0.1 g, 0.13 mmol) in acetonitrile (2 mL) and stirred for 16 hours. The volatiles were removed *in vacuo* and the solid was extracted with mixture of dichloromethane and hexane (2:1) and left for 7 days for crystallization. The colorless crystals were decanted and dried *in vacuo*. Yield 0.03 g, 47 %. **Mp.** 149 °C. **^1^H NMR** (25 °C, CD_3_CN, 500 MHz): *δ* = 0.88 (d, *^3^J*(^1^H, ^1^H) = 6.7 Hz, 3H, CH(C*H*_3_)_2_), 1.07 (d, *^3^J*(^1^H, ^1^H) = 6.7 Hz, 3H, CH(C*H*_3_)_2_), 1.15 (d, *^3^J*(^1^H, ^1^H) = 6.5 Hz, 3H, CH(C*H*_3_)_2_), 1.39-1.42 (m, 9H, CH(C*H*_3_)_2_), 1.51 (d, *^3^J*(^1^H, ^1^H) = 6.6 Hz, 3H, CH(C*H*_3_)_2_) 1.51 (d, *^3^J*(^1^H, ^1^H) = 6.7 Hz, 3H, CH(C*H*_3_)_2_), 4.42 (d, *^3^J*(^1^H, ^1^H) = 6.6 Hz, 1H, C*H*(CH_3_)_2_), 4.99 (d, *^3^J*(^1^H, ^1^H) = 6.7 Hz, 1H, C*H*(CH_3_)_2_), 5.37 (d, *^3^J*(^1^H, ^1^H) = 6.6 Hz, 1H, C*H*(CH_3_)_2_), 5.46 (d, *^3^J*(^1^H, ^1^H) = 6.7 Hz, 1H, C*H*(CH_3_)_2_), 7.21 (d, *^3^J*(^1^H, ^1^H) = 2.2 Hz, 1H, CH=C*H*), 7.31 (d, *^3^J*(^1^H, ^1^H) = 2.2 Hz, 1H, C*H*=CH), 7.35 (d, *^3^J*(^1^H, ^1^H) = 2.1 Hz, 1H, CH=C*H*), 7.48 (d, *^3^J*(^1^H, ^1^H) = 2.1 Hz, 1H, C*H*=CH) ppm. **^13^C{^1^H} NMR** (25 °C, CD_3_CN, 125.76 MHz): *δ* = 21.5, 21.9, 22.2, 22.4, 22.6, 23.0, 23.1, 24.1 (s, CH(*C*H_3_)_3_), 33.4 (s, B*C*H), 50.2, 50.6, 50.9, 51.5 (s, *C*H(CH_3_)_3_), 117.8, 118.7, 119.3, 119.7 (s, *C*H=*C*H), 129.3, 136.4 (s very broad, *C*N), 155.7, 155.2 (s very broad, N*C*N) ppm. **^11^B NMR** (25 °C, CD_3_CN, 160.46 MHz) *δ* = –44.2 (d, *^3^J*(^1^H, ^11^B) = 131.9 Hz, B3), –43.4 (d, *^3^J*(^1^H, ^11^B) = 116.3 Hz, B1), –35.4 (s, B9), –34.0 (d, *^3^J*(^1^H, ^11^B) = 148.8 Hz, B6), –22.0 (s, B10), –11.4 (d, *^3^J*(^1^H, ^11^B) = 132.8 Hz, B7), 2.5 (d, *^3^J*(^1^H, ^11^B) = 122.8 Hz, B4), 4.0 (d, *^3^J*(^1^H, ^11^B) = 139.3 Hz, B2).

**Spectroscopic characterization of *p*-2b*^i^*^Pr^**


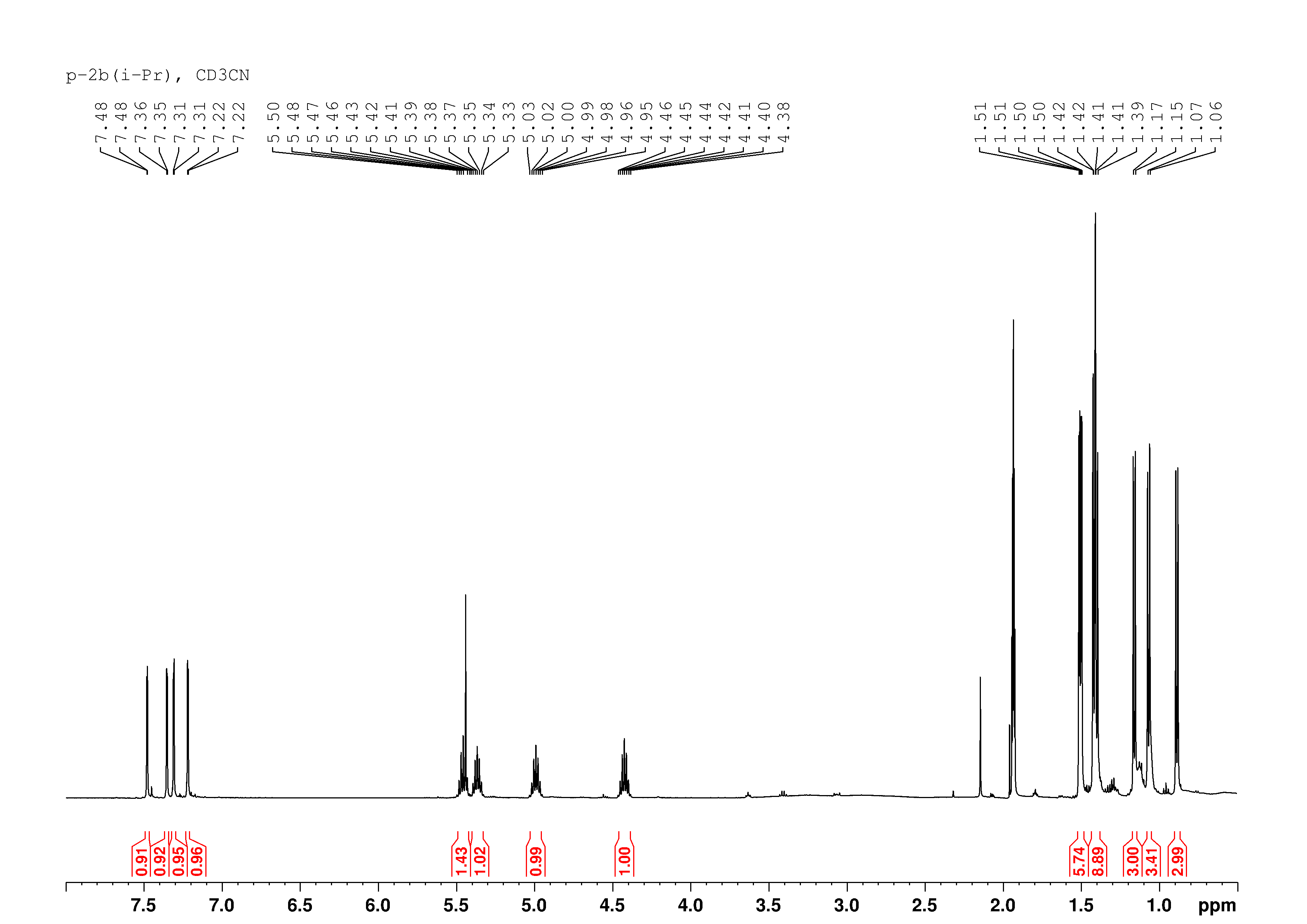


**Figure S92**. ^1^H NMR spectrum of **p-2b^iPr^**.


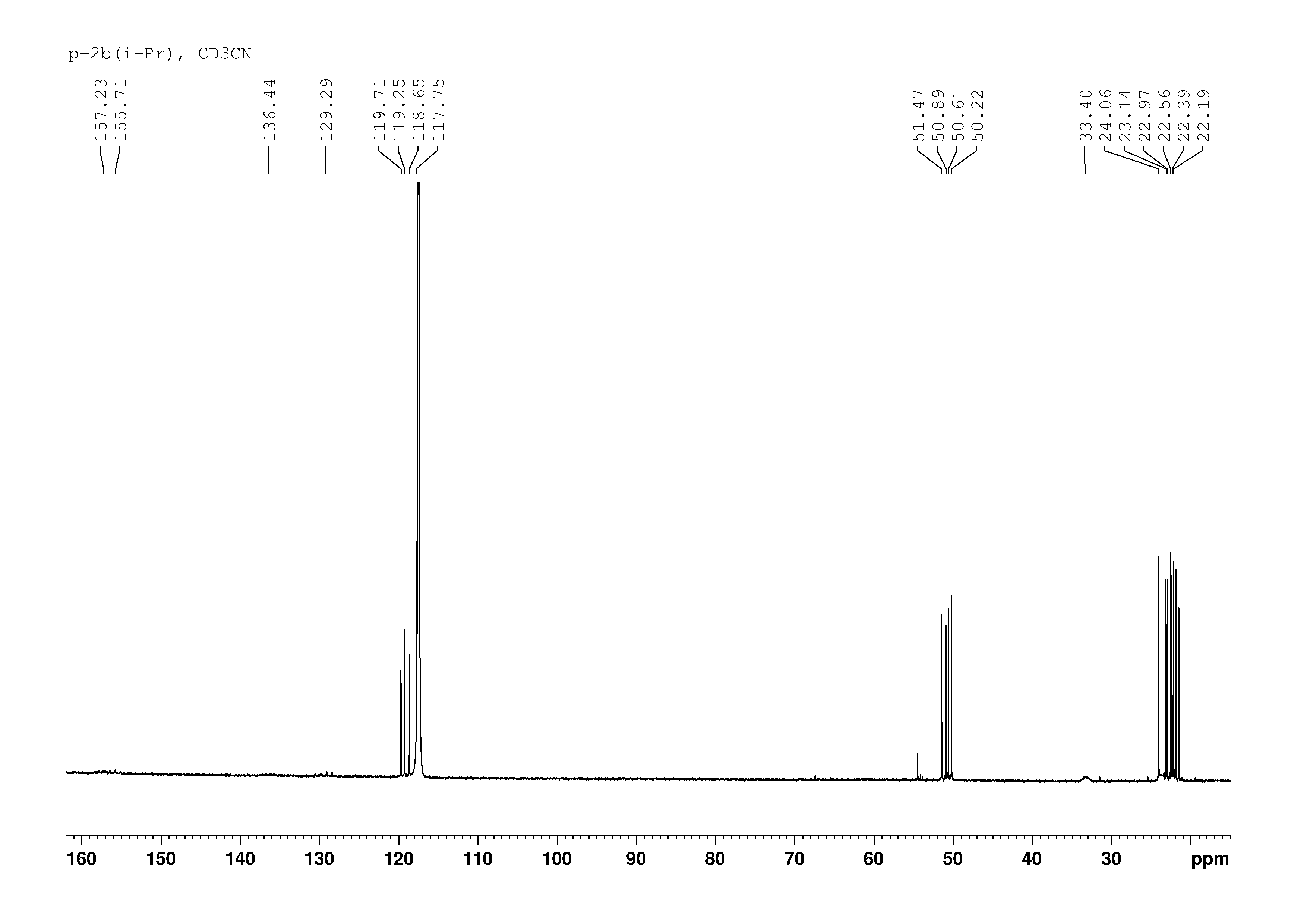


**Figure S93**. ^13^C{^1^H} NMR spectrum of **p-2b^iPr^**.


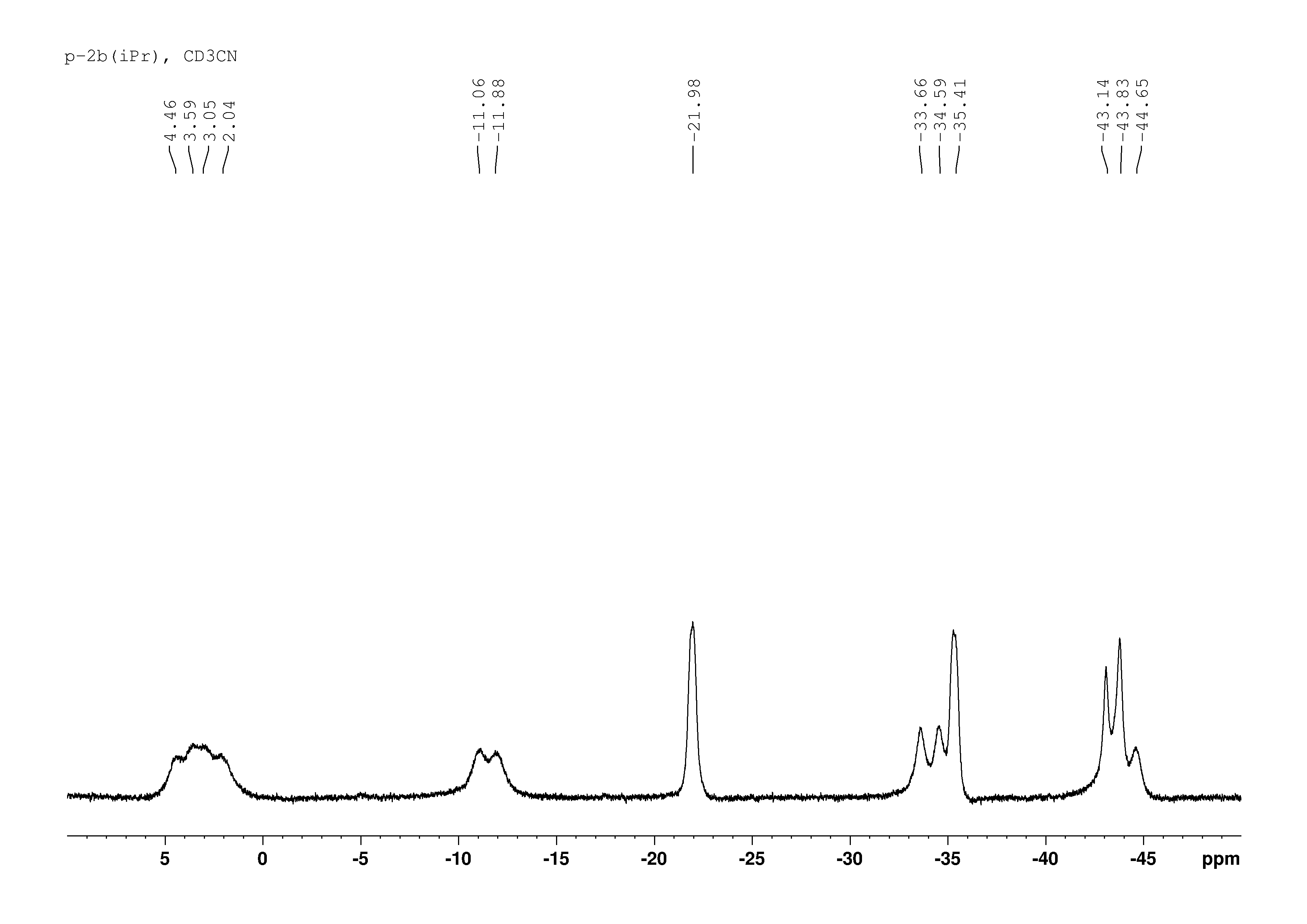


**Figure S94**. ^11^B NMR spectrum of **p-2b^iPr^**.


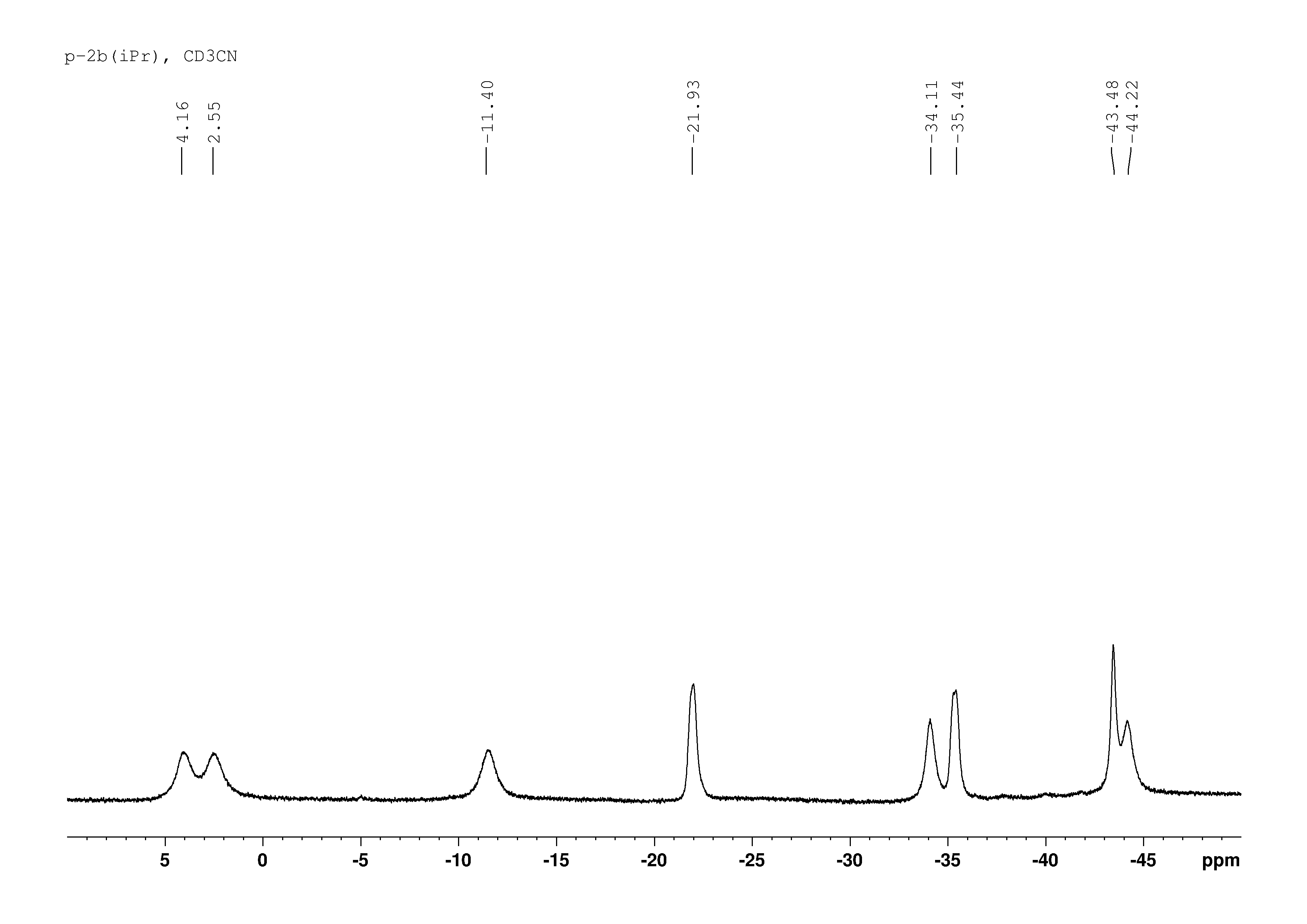


**Figure S95**. ^11^B{^1^H} NMR spectrum of **p-2b^iPr^**.

**Figure S96**. Mass spectrum of positively charged ions (ESI+, Orbitrap) for ***p*-2b*^i^*^Pr^**. The spectrum shows the protonated molecule and the sodium adduct of ***p*-2b*^iPr^*** (*m/z* 477.4 and *m/z* 499.4, respectively). The ion *m/z* 452.4 (C_21_H_42_N_5_B_8_^+^) is consistent with a by-product lacking one CN group.

**Figure S97**. Spectrum of positively charged ions (ESI+, Orbitrap @ R=500,000) for ***p*-2b*^i^*^Pr^** enlarged in the isotope cluster region (top) and simulated spectrum (bottom).

**Figure S98**. Spectrum of positively charged ions (ESI+, Orbitrap @ R=500,000) for ***p*-2b*^i^*^Pr^** enlarged in the monoisotopic peak region (top) and simulated spectrum (bottom). Theoretical mass for C_22_H_41_B_8_N_4_^+^: *m/z* 477.41317; experimental mass: *m/z* 477.41359; mass error 0.88ppm.

**Synthesis of [2,6-I*^i^*^Pr^_2_-1,10-C_2_B_8_H_8_][OTf]_2_ (*p*-2c*^i^*^Pr^)**

A solution of silver triflate (0.75 g, 2.9 mmol) in methanol (10 mL) was added to the solution of ***p*-2a*^i^*^Pr^** (0,72 g, 1.4 mmol) in methanol (15 mL), and the reaction mixture was stirred for ten minutes at room temperature. The suspension was filtered, and the volatiles were removed *in vacuo*, yielding ***p*-2c*^i^*^Pr^** as a white powder*.* Yield 0.98 g, 94 %. **Mp.** 238 °C**.** **^1^H NMR** (25 °C, CD_3_CN, 500 MHz): *δ* = 0.77 (s broad, 6H, CH(C*H*_3_)_2_), 1.47 (d, *^3^J*(^1^H, ^1^H) = 6.5 Hz, 12H, CH(C*H*_3_)_2_), 1.54 (s broad, 6H, CH(C*H*_3_)_2_), 3.66 (s broad, 2H, C*H*(CH_3_)_2_), 4.63 (s broad, 2H, C*H*(CH_3_)_2_), 7.75 (s broad, 2H, C*H*=C*H*), 7.85 (s, 2H, C*H*=C*H*), 7.98 (s, 2H, BC*H*) ppm. **^13^C NMR** (25 °C, CD_3_CN, 125.76 MHz): *δ* = 21.5, 23.0, 23.6, 24.0 (s, CH(*C*H_3_)_3_), 53.7 (s, *C*H(CH_3_)_3_), 106.5 (s B*C*H), 120.9 (s, *C*F_3_) 123.8, 124.6 (s, *C*H=*C*H), 137.4 (s very broad, N*C*N) ppm. **^11^B NMR** (25 °C, CD_3_CN, 160.46 MHz) *δ* = –12.2 (m broad), –10.5 (m broad), –8.1 (m broad) ppm.

**Spectroscopic characterization of *p*-2c*^i^*^Pr^**


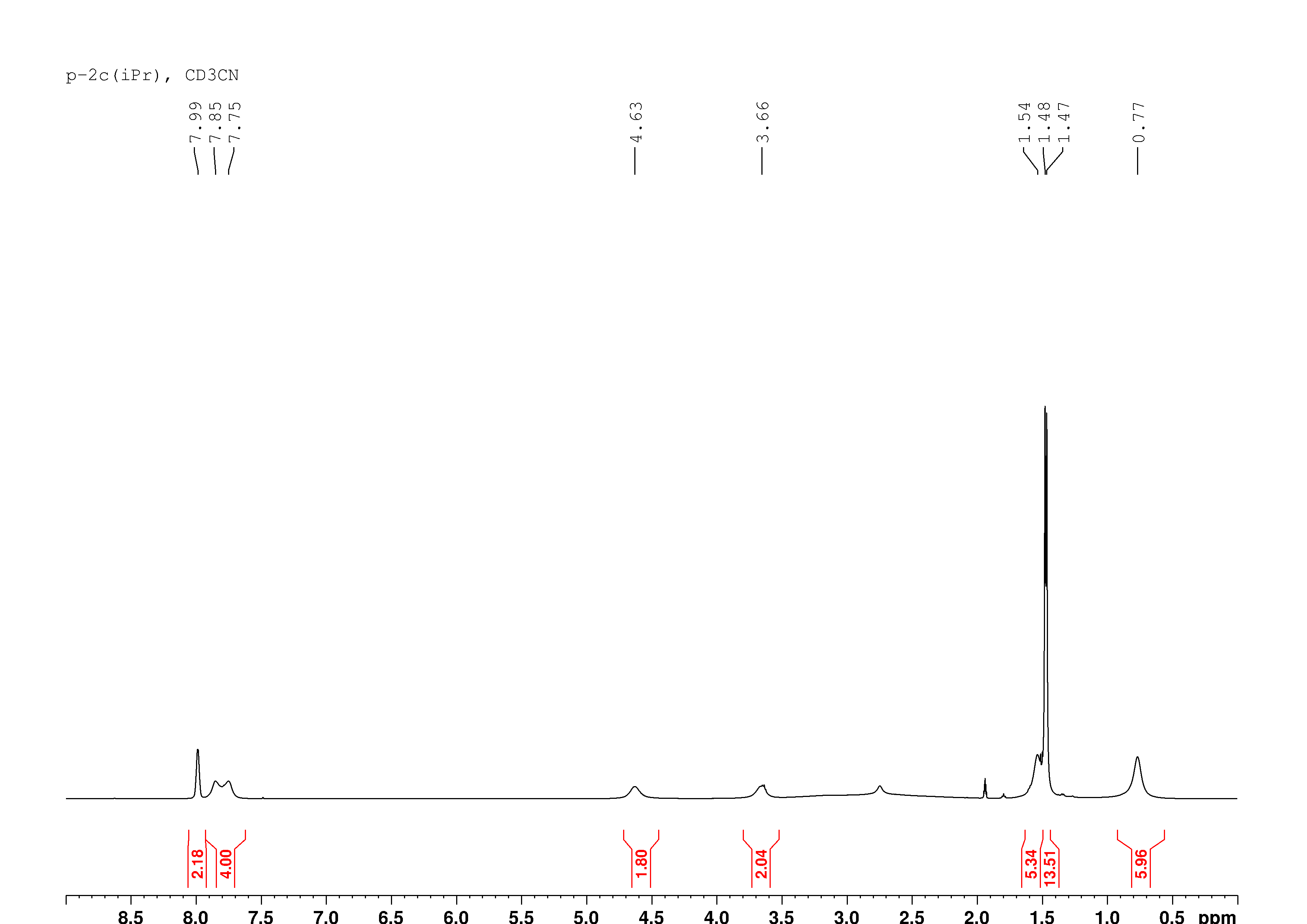


**Figure S99**. ^1^H NMR spectrum of ***p-*2c*^i^*^Pr^**.


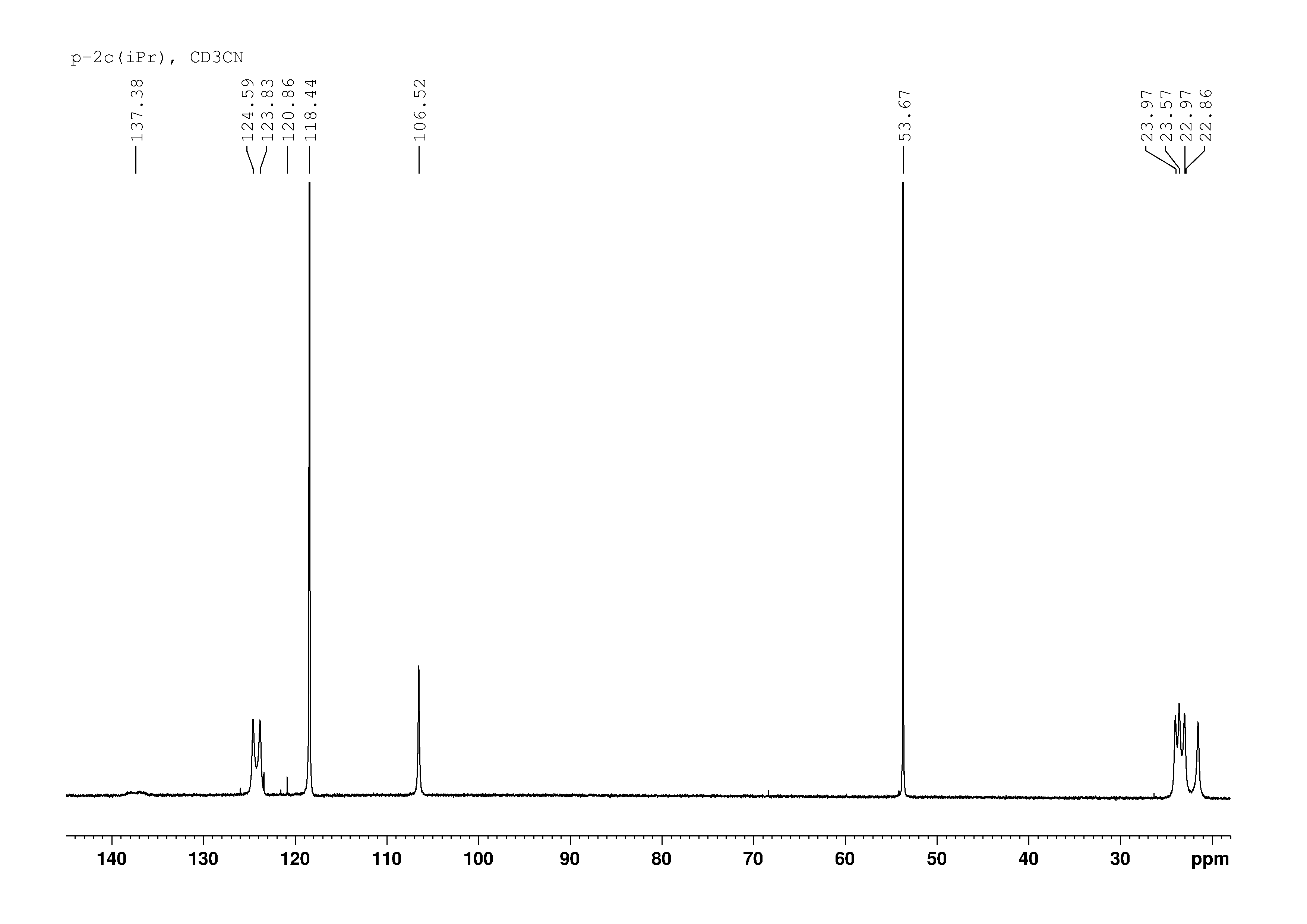


**Figure S100**. ^13^C{^1^H} NMR spectrum of ***p-*2c*^i^*^Pr^**.


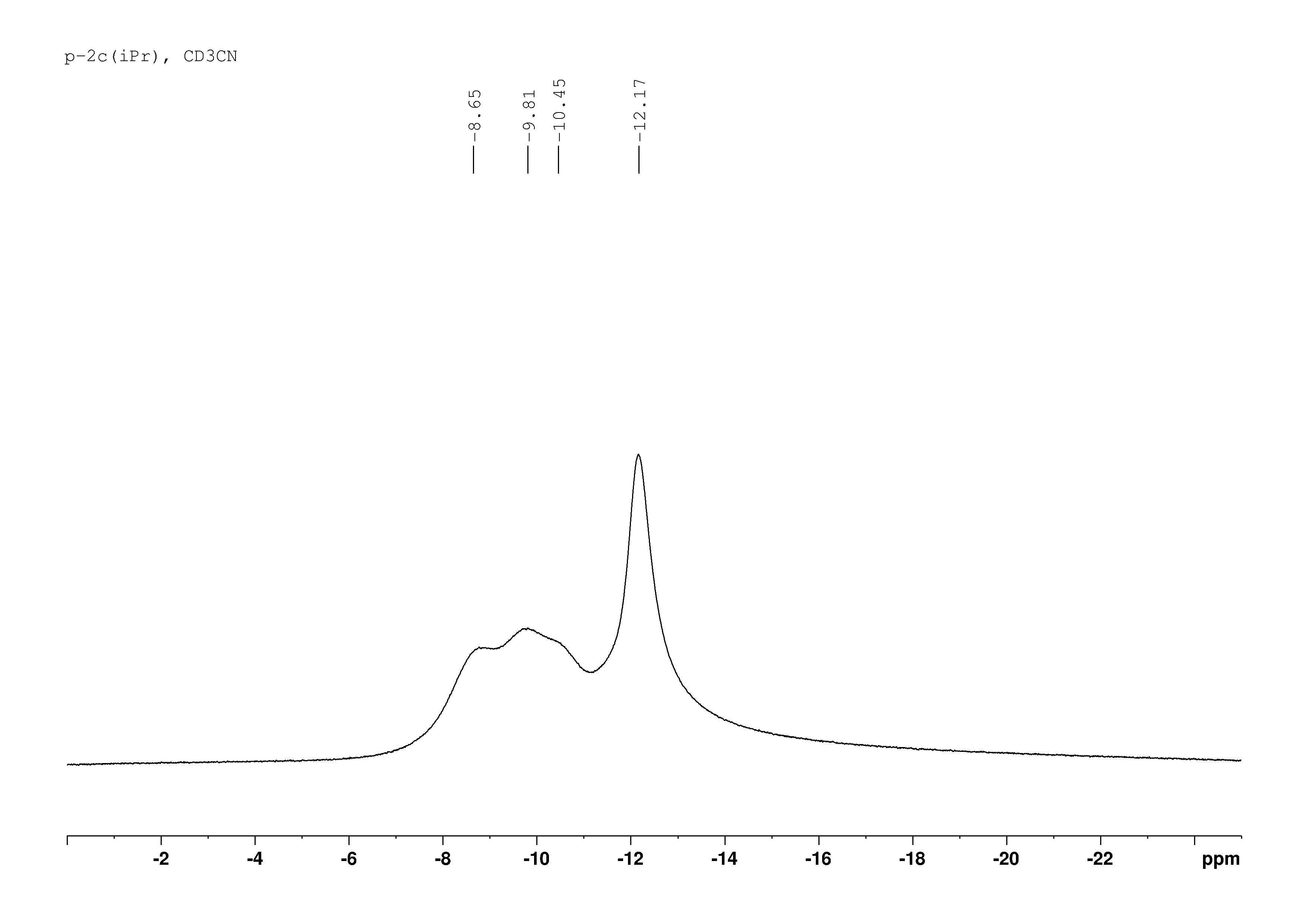


**Figure S101**. ^11^B NMR spectrum of ***p-*2c*^i^*^Pr^**.


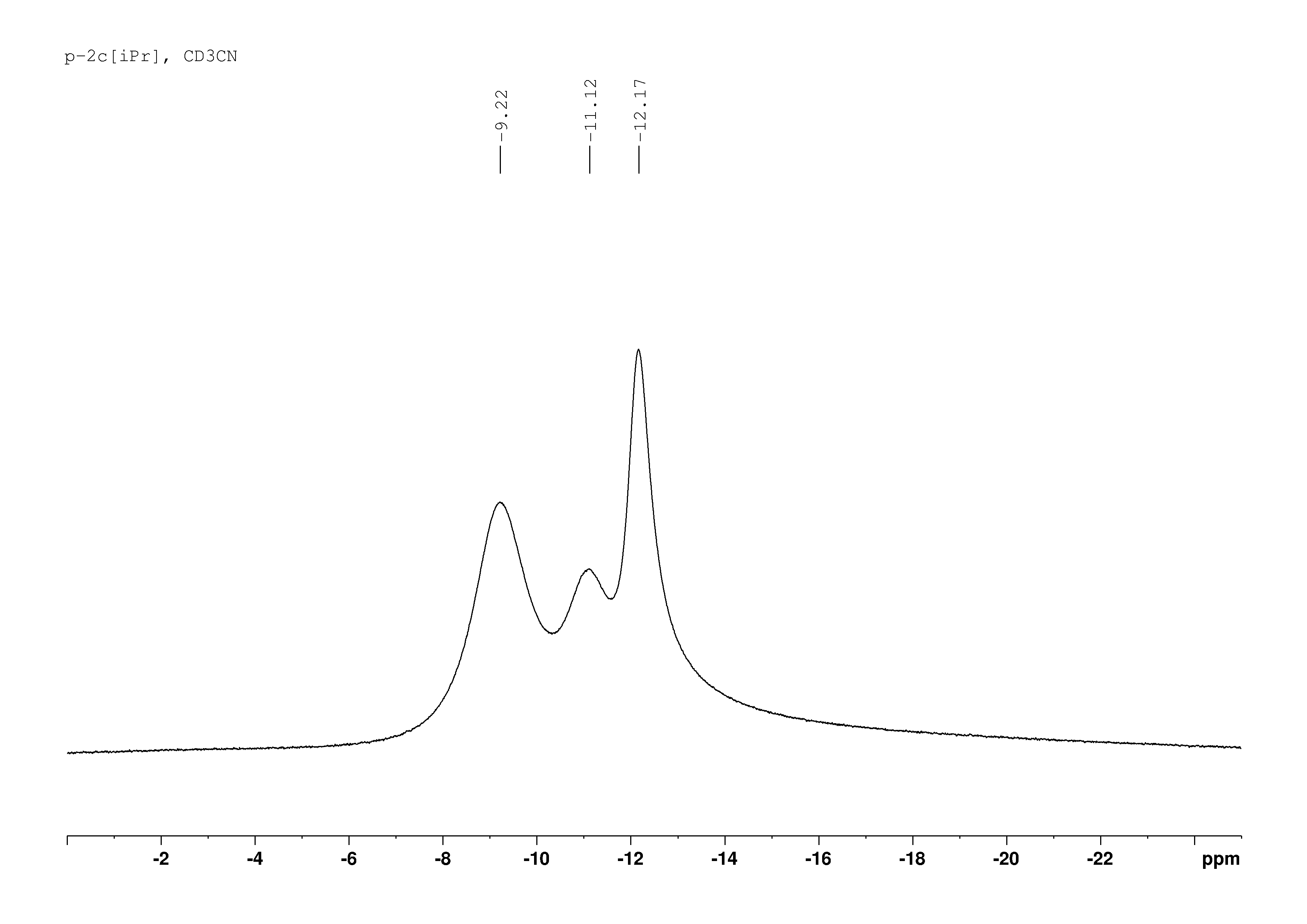


**Figure S102**. ^11^B{^1^H} NMR spectrum of ***p-*2c*^i^*^Pr^**.

**Figure S103**. Mass spectrum of positively charged ions (ESI+, Orbitrap) for ***p-*2c*^i^*^Pr^**. The spectrum shows the dication of ***p*-2c*^iPr^*** (*m/z* 212.2). The other ions can be explained as follows: *m/z* 273.3 (C_11_H_25_N_2_B_8_^+^), the cation lacking one **:I*^i^*^Pr^**; *m/z* 441.4 (C_20_H_41_N_4_B_8_O^+^), the dication with OH^−^; *m/z* 573.3 (C_21_H_40_N_4_B_8_F_3_O_3_S^+^), the dication with OTf^−^; m/z 745.3 (C_22_H_40_N_4_B_8_F_6_O_6_S_2_Na^+^), sodium adduct of ***p*-2c*^iPr^***.

**Figure S104**. Spectrum of positively charged ions (ESI+, Orbitrap @ R=500,000) for ***p-*2c*^i^*^Pr^** enlarged in the isotope cluster region (top) and simulated spectrum (bottom) for a singly charged species resulting from the association of the doubly charged molecule with triflate anion.

**Figure S105**. Spectrum of positively charged ions (ESI+, Orbitrap @ R=500,000) for ***p-*2c*^i^*^Pr^** enlarged in the monoisotopic peak region (top) and simulated spectrum (bottom) for a singly charged species resulting from the association of the doubly charged molecule with triflate anion. Theoretical mass for C_21_H_40_B_8_F_3_N_4_O_3_S^+^: *m/z* 573.35122; experimental mass: *m/z* 573.35161; mass error 0.69 ppm.

**Figure S106**. Spectrum of positively charged ions (ESI+, Orbitrap @ R=500,000) for ***p-*2c*^i^*^Pr^** enlarged in the isotope cluster region (top) and simulated spectrum (bottom) for the doubly charged molecule.

**Figure S107**. Spectrum of positively charged ions (ESI+, Orbitrap @ R=500,000) for ***p-*2c*^i^*^Pr^** enlarged in the monoisotopic peak region (top) and simulated spectrum (bottom) for the doubly charged molecule. Theoretical mass for C_20_H_40_B_8_N_4_^2+^: *m/z* 212.19932; experimental mass: *m/z* 212.19927; mass error −0.25 ppm.

**Synthesis of [2,4-I*^i^*^Pr^_2_-6,9-(DMAP)_2_-5,10-C_2_B_8_H_8_][OTf]_2_ (*p*-2d*^i^*^Pr^)**

Solution of DMAP (0.2 g, 1.7 mmol) in acetonitrile (2 mL) was added dropwise to the solution of ***p*-2c*^i^*^Pr^** (0.24 g, 0.33 mmol) in acetonitrile (2 mL), and the reaction mixture was stirred for one day at room temperature. The suspension was filtered, and volatiles were removed *in vacuo.* The solid was twice washed with tetrahydrofuran (1 mL), yielding ***p*-2d*^i^*^Pr^** as a white powder*.* Yield 0.245 g, 76 %. **Mp.** 149 °C. **^1^H NMR** (25 °C, CD_3_CN, 500 MHz): *δ* = 1.19 (d, *^3^J*(^1^H, ^1^H) = 6.5 Hz, 12H, CH(C*H*_3_)_2_), 1.32 (d, *^3^J*(^1^H, ^1^H) = 6.6 Hz, 12H, CH(C*H*_3_)_2_), 1.87 (s broad, 2H, BCH), 3.12 (s, 12H, C*H*_3_), 4.96 (h, *^3^J*(^1^H, ^1^H) = 6.6 Hz, 4H, C*H*(CH_3_)_2_), 6.73 (d, *^3^J*(^1^H, ^1^H) = 8.23 Hz, 4H, *o*-C_5_*H*_4_N**)**, 7.52 (s, 4H C*H*=C*H*), 8.05 (d, *^3^J*(^1^H, ^1^H) = 7.9 Hz, 4H, *m*-C_5_*H*_4_N) ppm. **^13^C{^1^H} NMR** (25 °C, CD_3_CN, 125.76 MHz): *δ* = 22.8, 23.0 (s, CH(*C*H_3_)_3_), 32.8 (s B*C*H), 39.7 (s, *C*H_3_), 51.3 (s, *C*H(CH_3_)_3_), 107.8 (s, *m*-*C*_5_H_4_N), 121.2 (s, *C*H=*C*H), 144.8 (s, *o*-*C*_5_H_4_N), 148.8 (s very broad, N*C*N), 156.4 (s, *p*-*C*_5_H_4_N) ppm. **^11^B NMR** (25 °C, CD_3_CN, 160.46 MHz) *δ* = –41.9 (s, 2B, B1,3), –20.7 (s broad, 2B, B6,9), –8.6, –7.4 (s broad, 4B, B2,4,7,8) ppm.

**Spectroscopic characterization of *p*-2d*^i^*^Pr^**


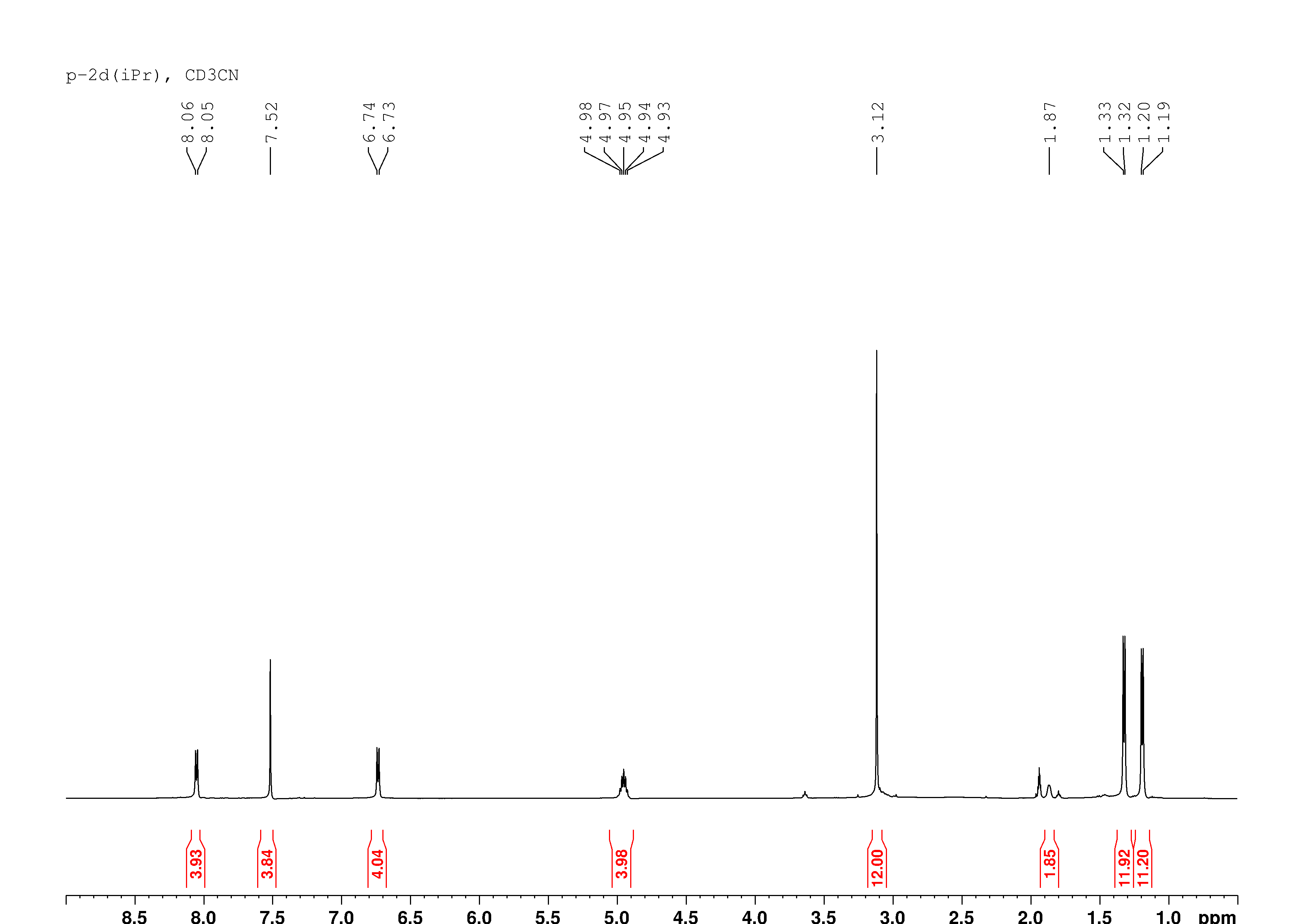


**Figure S108**. ^1^H NMR spectrum of ***p-*2d*^i^*^Pr^**.


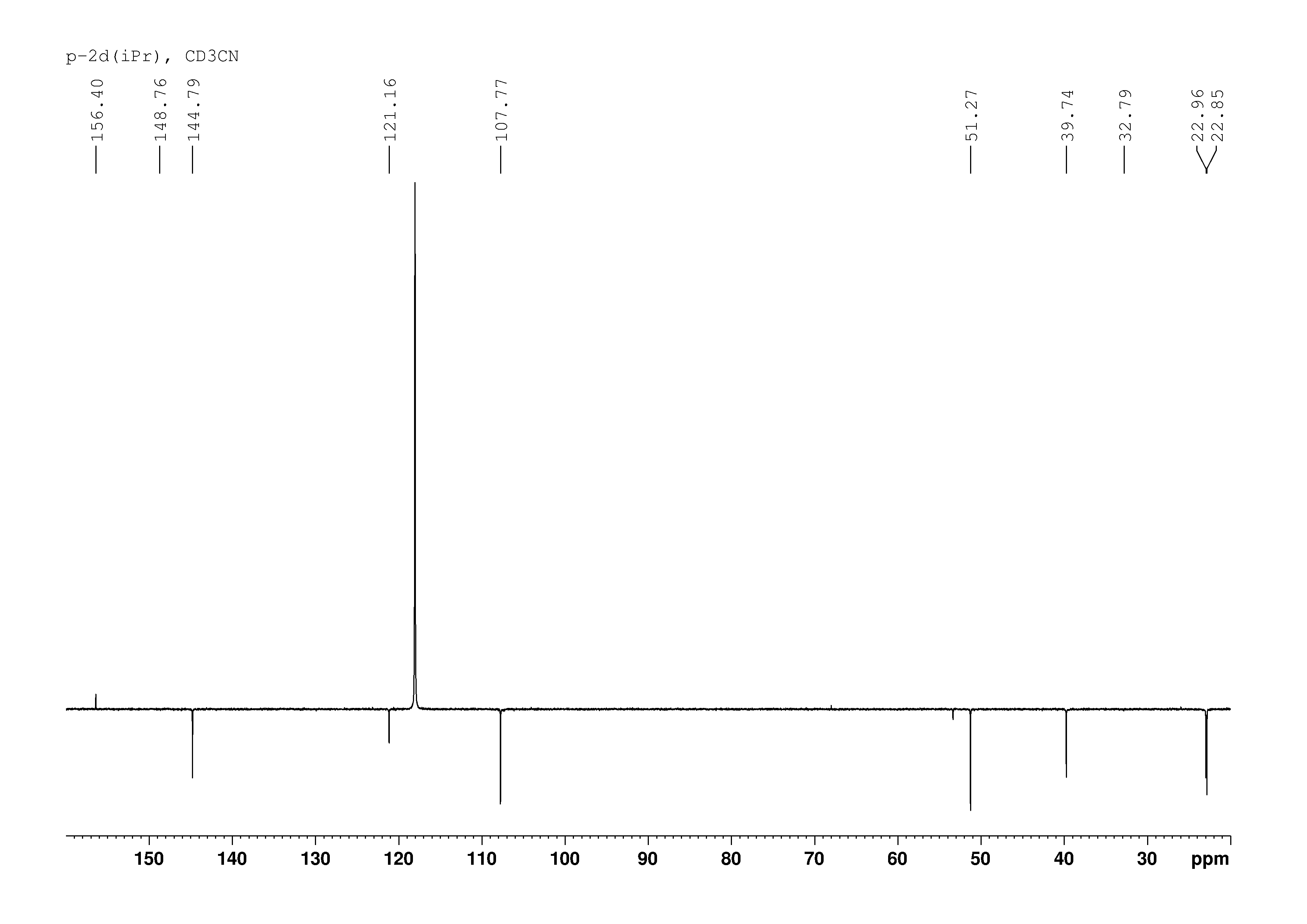


**Figure S109**. ^13^C{^1^H} NMR spectrum of ***p-*2d*^i^*^Pr^**.


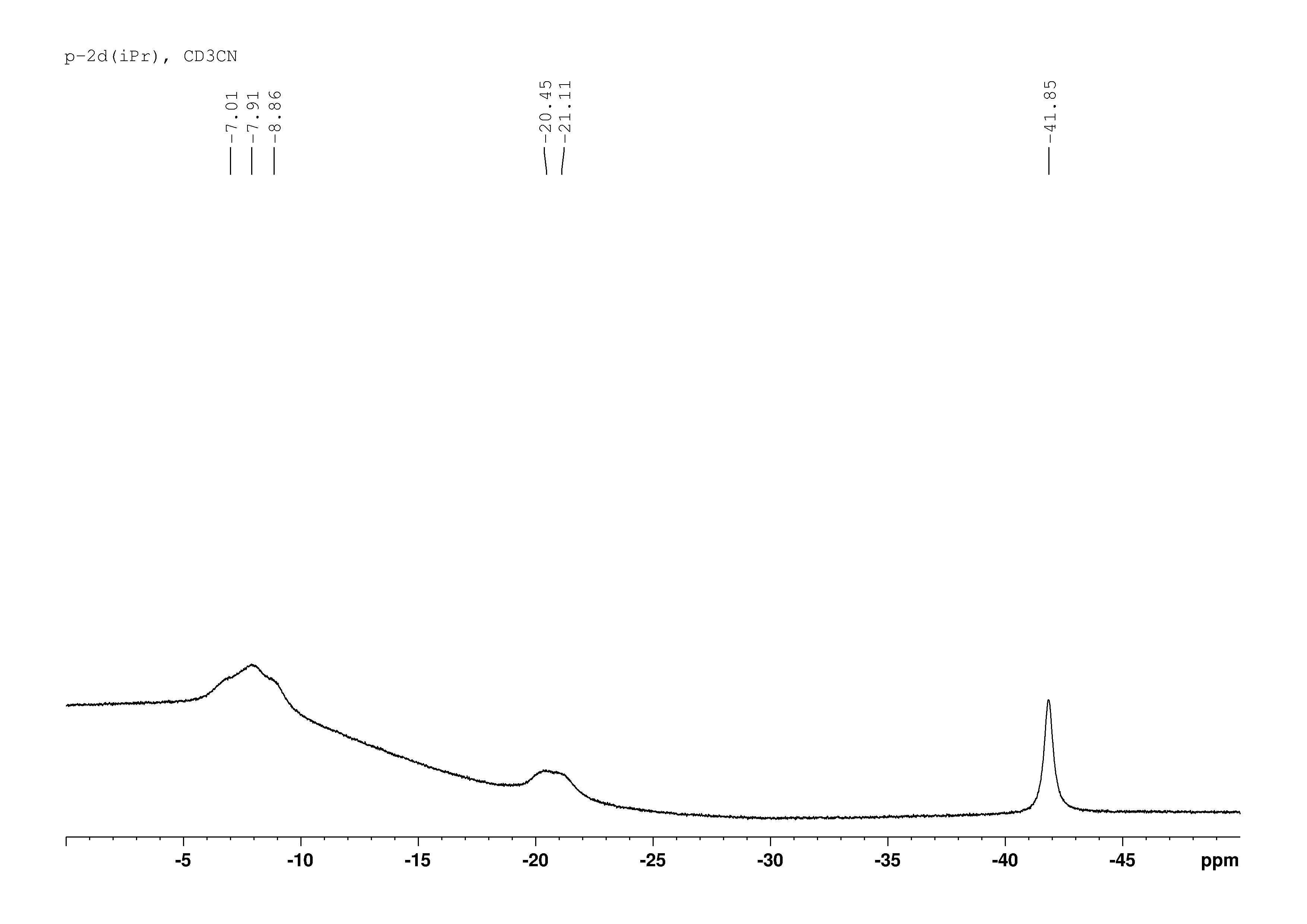


**Figure S110**. ^11^B NMR spectrum of ***p-*2d*^i^*^Pr^**.


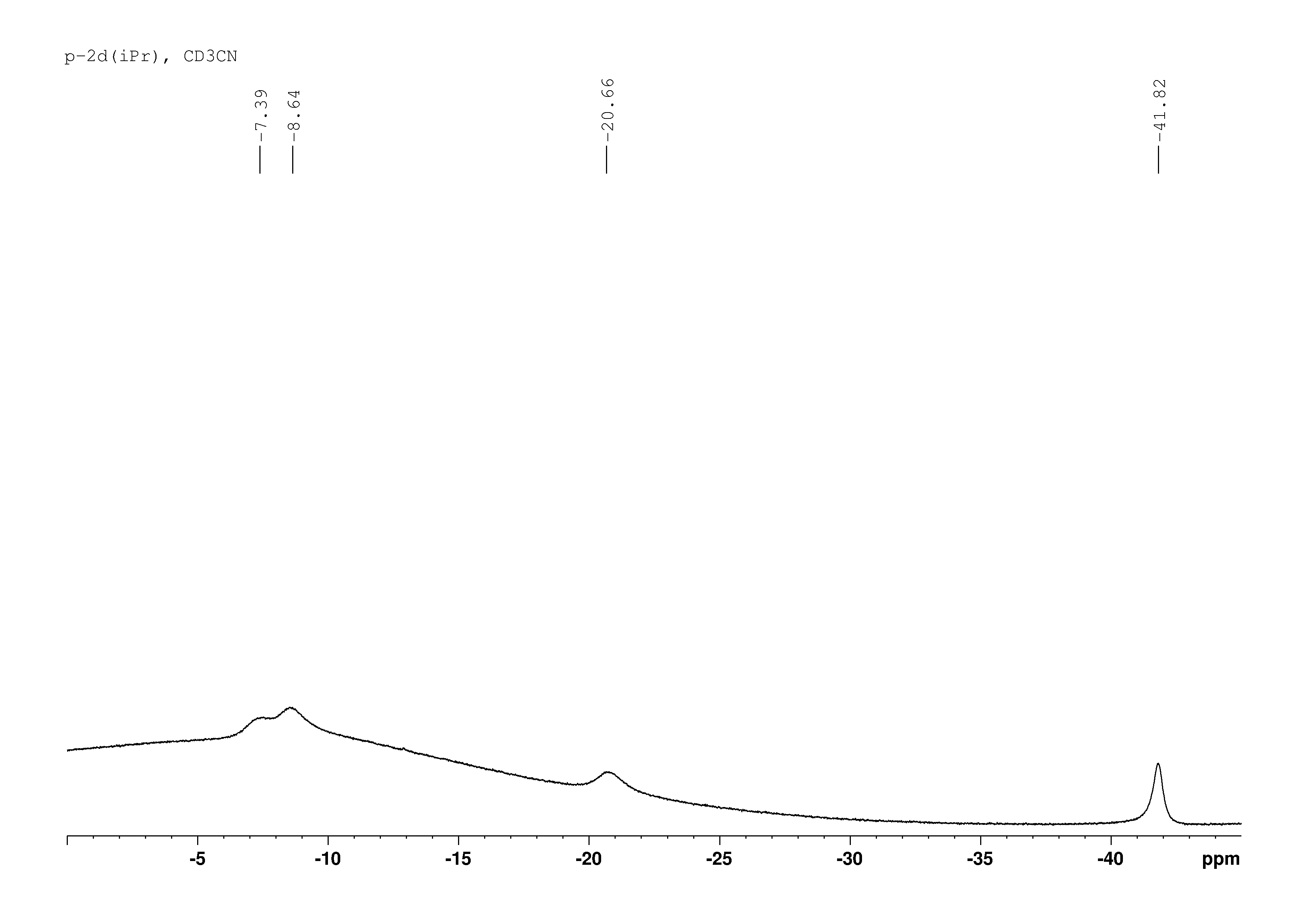


**Figure S111**. ^11^B{^1^H} NMR spectrum of ***p-*2d*^i^*^Pr^**.

**Figure S112**. Mass spectrum of positively charged ions (ESI+, Orbitrap) for ***p-*2d*^i^*^Pr^**. The spectrum shows the dication of ***p*-2d*^iPr^*** (*m/z* 334.3) and the the dication with OTf^−^ (*m/z* 817.5, C_35_H_60_N_8_B_8_F_3_O_3_S^+^).

**Figure S113**. Spectrum of positively charged ions (ESI+, Orbitrap @ R=500,000) for ***p-*2d*^i^*^Pr^** enlarged in the isotope cluster region (top) and simulated spectrum (bottom) for the doubly charged molecule.

**Figure S114**. Spectrum of positively charged ions (ESI+, Orbitrap @ R=500,000) for ***p-*2d*^i^*^Pr^** enlarged in the isotope cluster region (top) and simulated spectrum (bottom) for a singly charged species resulting from the association of the doubly charged molecule with triflate anion.

**Figure S115**. Spectrum of positively charged ions (ESI+, Orbitrap @ R=500,000) for ***p-*2d*^i^*^Pr^** enlarged in the monoisotopic peak region (top) and simulated spectrum (bottom) for a singly charged species resulting from the association of the doubly charged molecule with triflate anion. Theoretical mass for C_35_H_60_B_8_F_3_N_8_O_3_S^+^: *m/z* 817.52001; experimental mass: *m/z* 817.52027; mass error 0.31 ppm.

**Synthesis of** **2,4-I*^i^*^Pr^_2_-6,9-C_2_B_8_H_8_ (*p*-2e*^i^*^Pr^)**

The flask was charged with solution of triethylamine (0.13 mL, 0.9 mmol) and ***p*-2c*^i^*^Pr^** (0.13 g, 0.2 mmol) in acetonitrile (2 mL) was filled with H_2_ atmosphere at 1.5 bar and sealed. The reaction mixture was stirred for 3 days. The resulting suspension was concentrated to 1 mL, and the solution was filtered. The solid was washed with benzene (2 mL) and extracted with tetrahydrofuran (2 mL). The volatiles were removed in *vacuo*, and the solid was extracted with dichloromethane (2 mL) and water (1 mL). The organic layer was separated, dried over MgSO_4_ and filtered. The volatiles were removed in *vacuo,* yielding ***p*-2e*^i^*^Pr^** as a white powder. Yield 10 mg, 13 %. **Mp:** 70 °C with decomposition. C_20_H_40_B_8_N_4_ (424.96). **^1^H NMR** (25 °C, THF-D_8_, 500 MHz): *δ* = 1.30 (d, *^3^J*(^1^H, ^1^H) = 6.6 Hz, 12H, CH(C*H*_3_)_2_), 1.39 (d, *^3^J*(^1^H, ^1^H) = 6.7 Hz, 12H, CH(C*H*_3_)_2_), 1.50 (s broad, 2H, BC*H*), 5.86 (d, *^3^J*(^1^H, ^1^H) = 6.7 Hz, 4H, C*H*(CH_3_)_2_), 7.24 (s, 4H, C*H*=C*H*) ppm. **^13^C NMR** (25 °C, THF-D_8_, 125.76 MHz): *δ* = 21.8, 22.8 (s, CH(*C*H_3_)_3_), 46.5 (s broad, B*C*H), 48.8 (s, *C*H(CH_3_)_3_), 116.0 (s, *C*H=*C*H), 162.8 (s broad, N*C*N). **^11^B NMR** (25 °C, THF-D_8_, 160.46 MHz) *δ* = −36.2 (d, *^1^J*(^1^H, ^11^B) = 144.8 Hz, 2B, B1,3), −19.6 (s, 2B, B2,4), −7.3 (d broad, *^1^J*(^1^H, ^11^B) = 132.5 Hz, 2B, B5,7,8,10), −5.9 (d, *^1^J*(^1^H, ^11^B) = 137.8 Hz, 2B, B5,7,8,10) ppm.


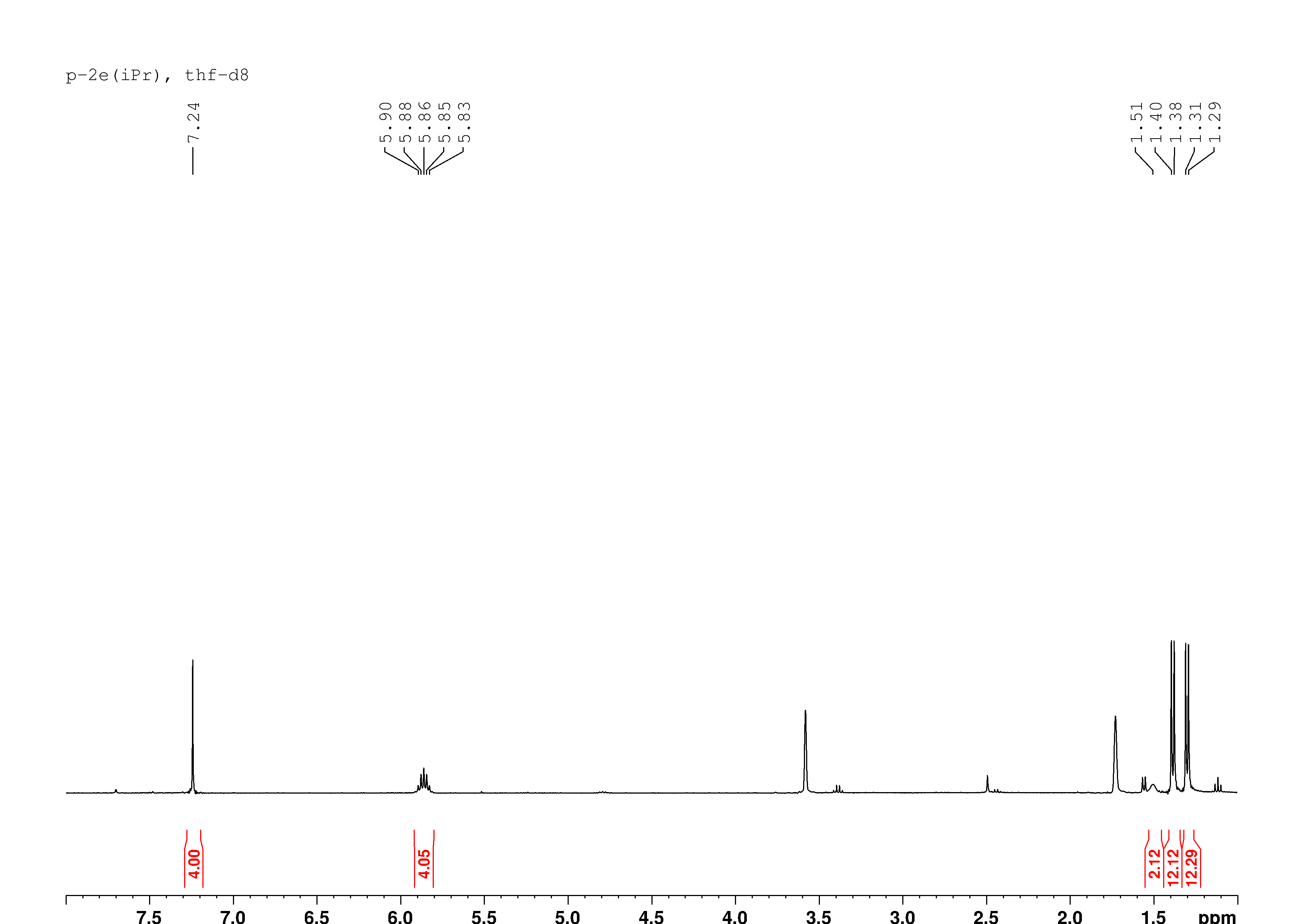


**Figure S116**. ^1^H NMR spectrum of ***p-*2e*^i^*^Pr^**.


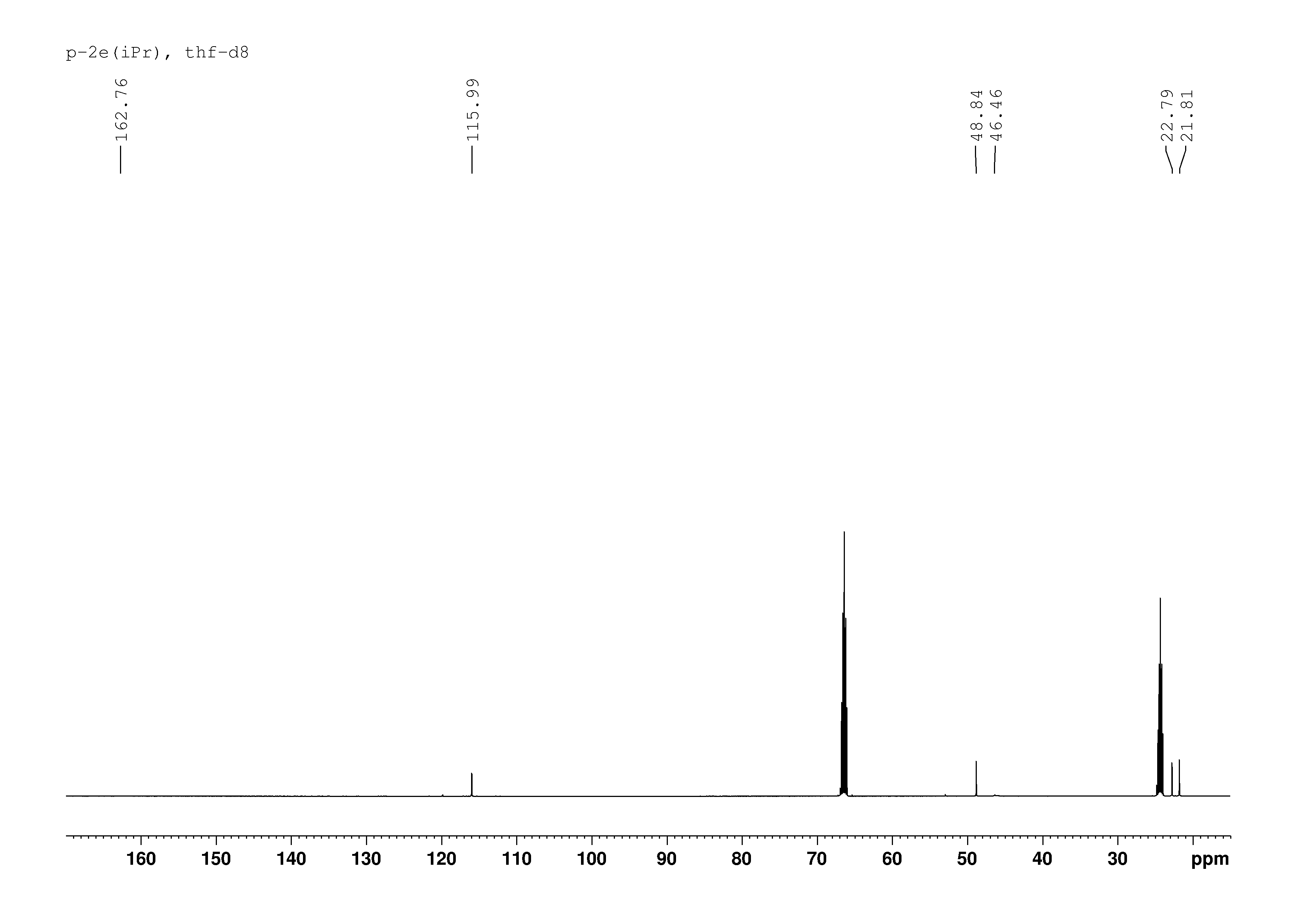


**Figure S117**. ^13^C{^1^H} NMR spectrum of ***p-*2e*^i^*^Pr^**.


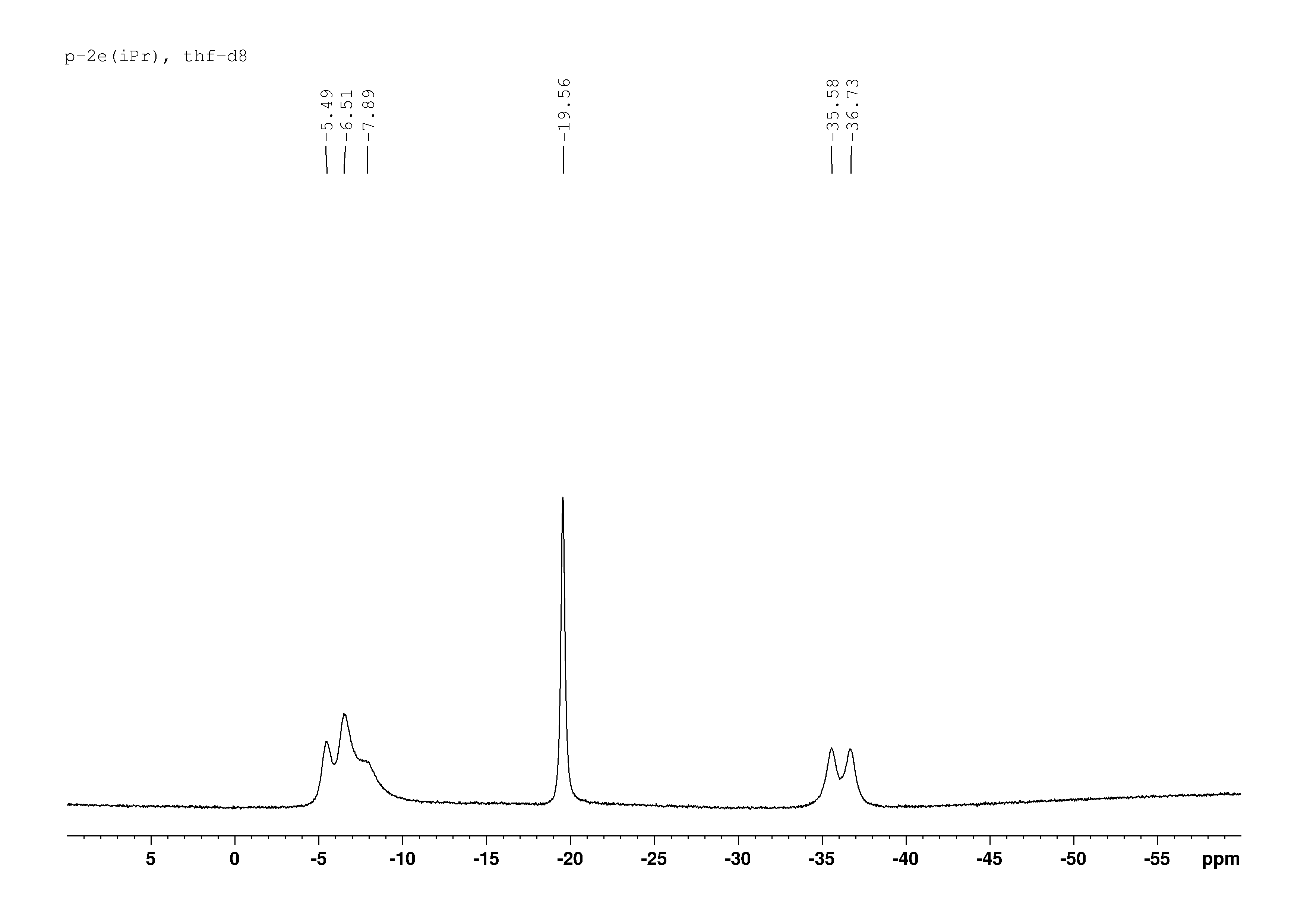


**Figure S118**. ^11^B NMR spectrum of ***p-*2e*^i^*^Pr^**.


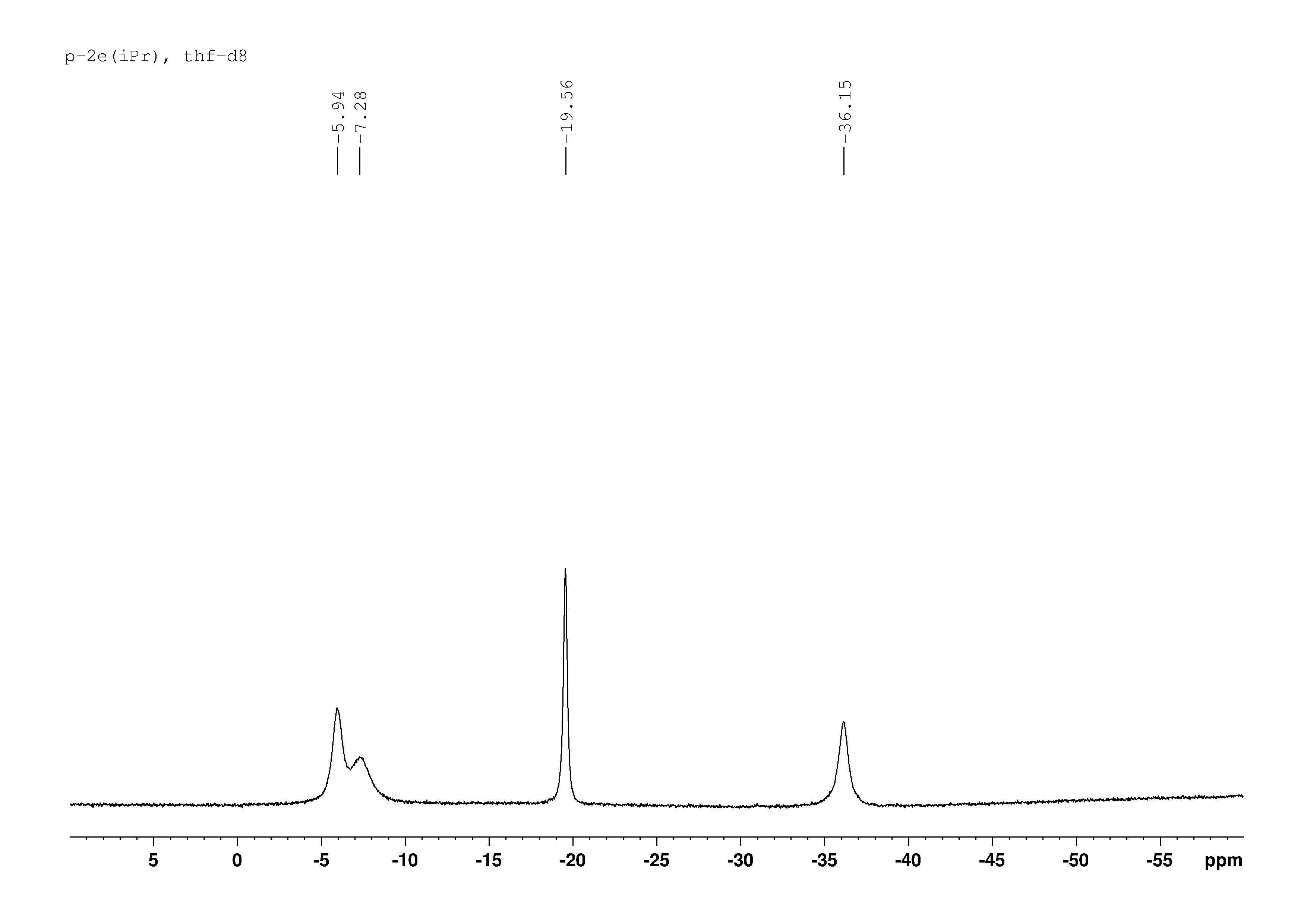


**Figure S119**. ^11^B{^1^H} NMR spectrum of ***p-*2e*^i^*^Pr^**.

**Figure S120**. Mass spectrum of positively charged ions (ESI+, Orbitrap) for ***p-*2e*^i^*^Pr^**. The ion at *m/z* 273.3 (C_11_H_25_N_2_B_8_^+^) corresponds to a by-product with one **:I*^i^*^Pr^** ligand; its oxidized form is at *m/z* 289.3 (C_11_H_25_N_2_B_8_O^+^). The spectrum base peak at *m/z* 441.4 (C_20_H_41_N_4_B_8_O^+^) is an oxidized form of ***p*-2e*^iPr^***; its sodium adduct appears at *m/z* 463.4 (C_20_H_40_N_4_B_8_ONa^+^).

**Figure S121**. Spectrum of positively charged ions (ESI+, Orbitrap @ R=500,000) for oxidized ***p‑*2e*^i^*^Pr^** enlarged in the isotope cluster region (top) and simulated spectrum (bottom).

**NMR data of skeletal atoms**

**Table S1**. Experimental ^13^C (**bold**) and ^11^B NMR shifts of the skeletal carbon (**bold**) and boron atoms. Molecular structures are listed below the table and correlation diagram of the ^11^B NMR shifts in Figure SX.

|  | **1** | **2** | **3** | **4** | **5** | **6** | **7** | **8** | **9** | **10** |
| --- | --- | --- | --- | --- | --- | --- | --- | --- | --- | --- |
| **p-C_2_B_8_H_10_** | **103.3** | −13 | –13 | –13 | –13 | –13 | –13 | –13 | –13 | **103.2** |
| **p-2^iPr^** | −44.5 | −7.1 | −44.5 | −7.1 | **28.6** | −36.1 | −11.9 | **28.6** | −36.1 | -11.9 |
| **p-2a^iPr^** | **107.5** | −9.7 | −9.7 | −9.7 | −9.7 | −12.0 | −9.7 | −9.7 | −12.0 | **107.5** |
| **p-2b^iPr^** | −43.4 | −2.5 | −44.2 | −4.0 | **33.4** | −34.0 | −11.4 | **33.4** | -35.4 | −22.0 |
| **p-2d^iPr^** | −41.9 | −7.4 | −41.9 | −7.4 | −8.6 | −20.7 | **32.8** | −8.6 | -20.7 | **32.8** |
| **p-2e^iPr^** | −36.2 | −19.6 | −36.2 | −19.6 | −7.3 | **46.5** | −5.9 | −7.3 | **46.5** | −5.9 |


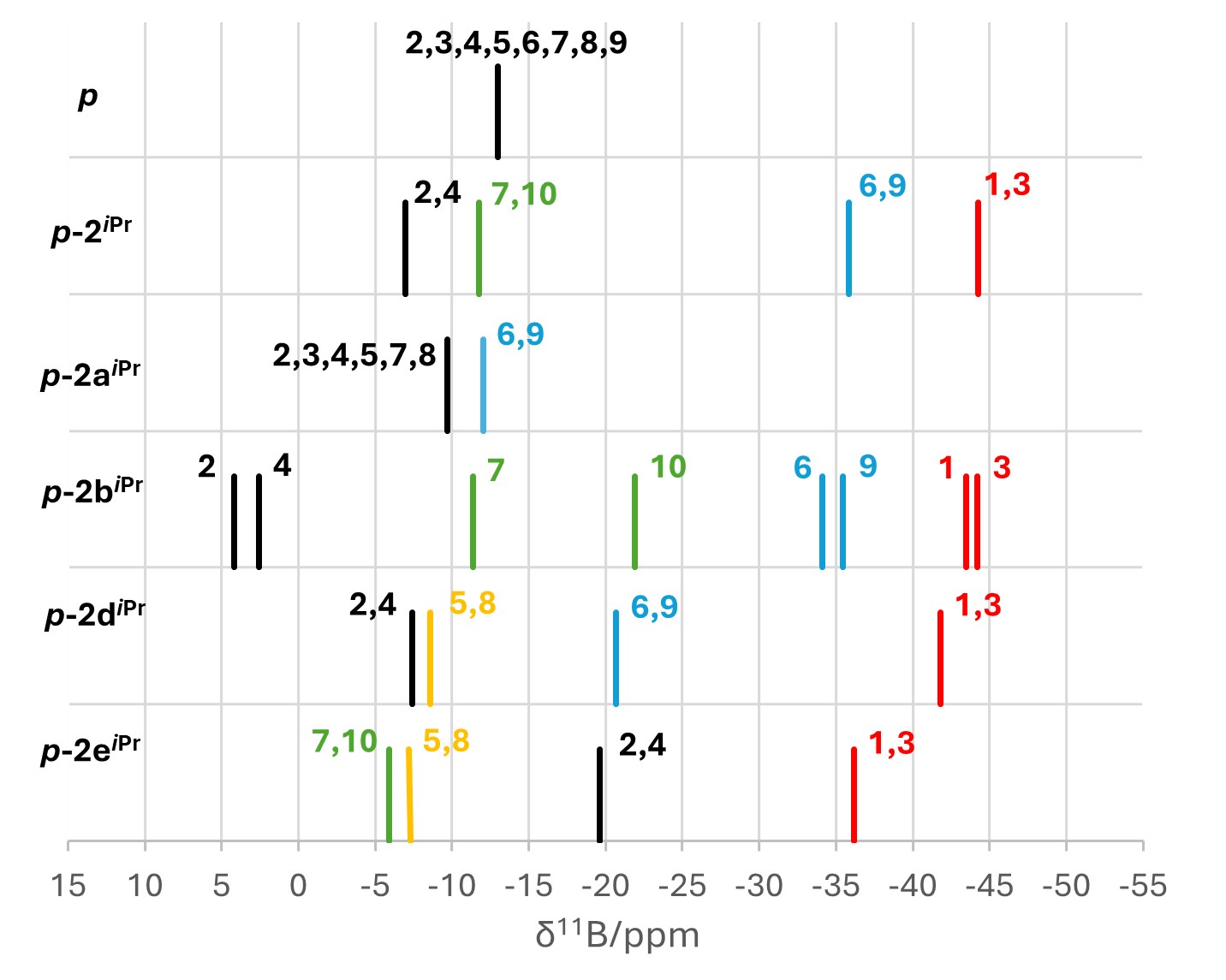


**Crystallographic data**

# **
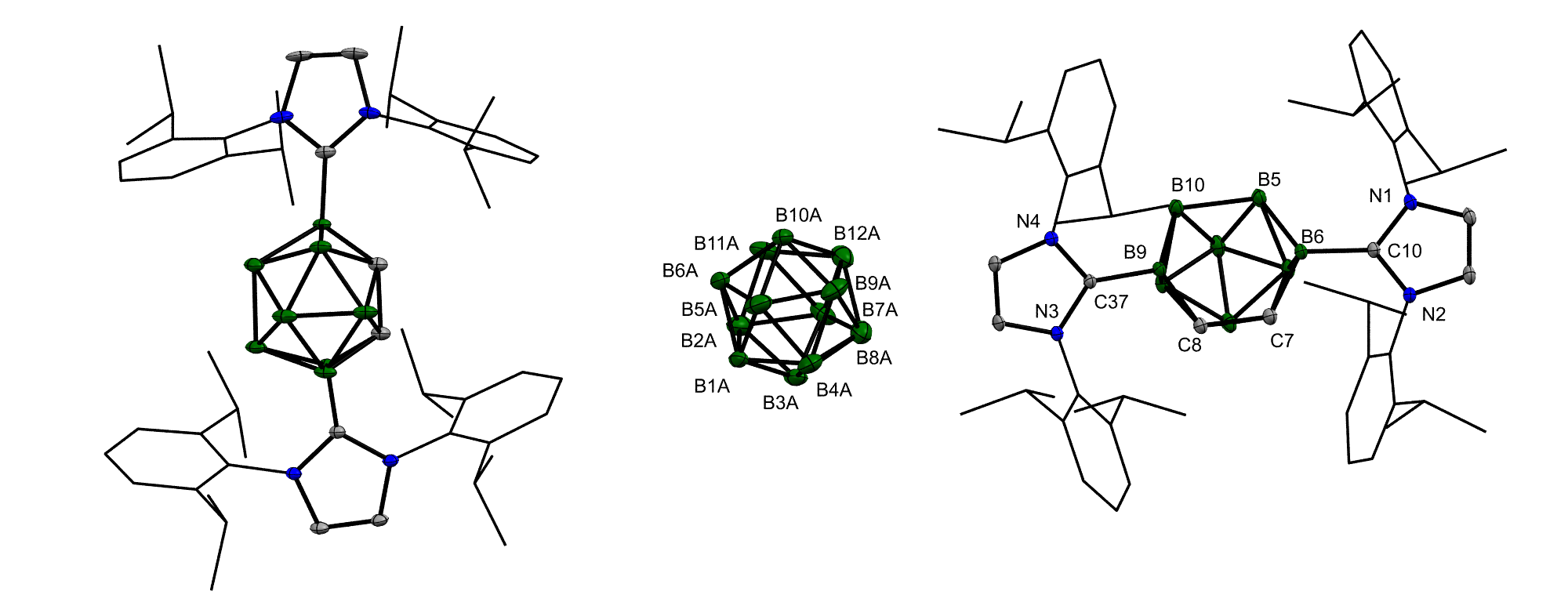
**

**A**

**B**


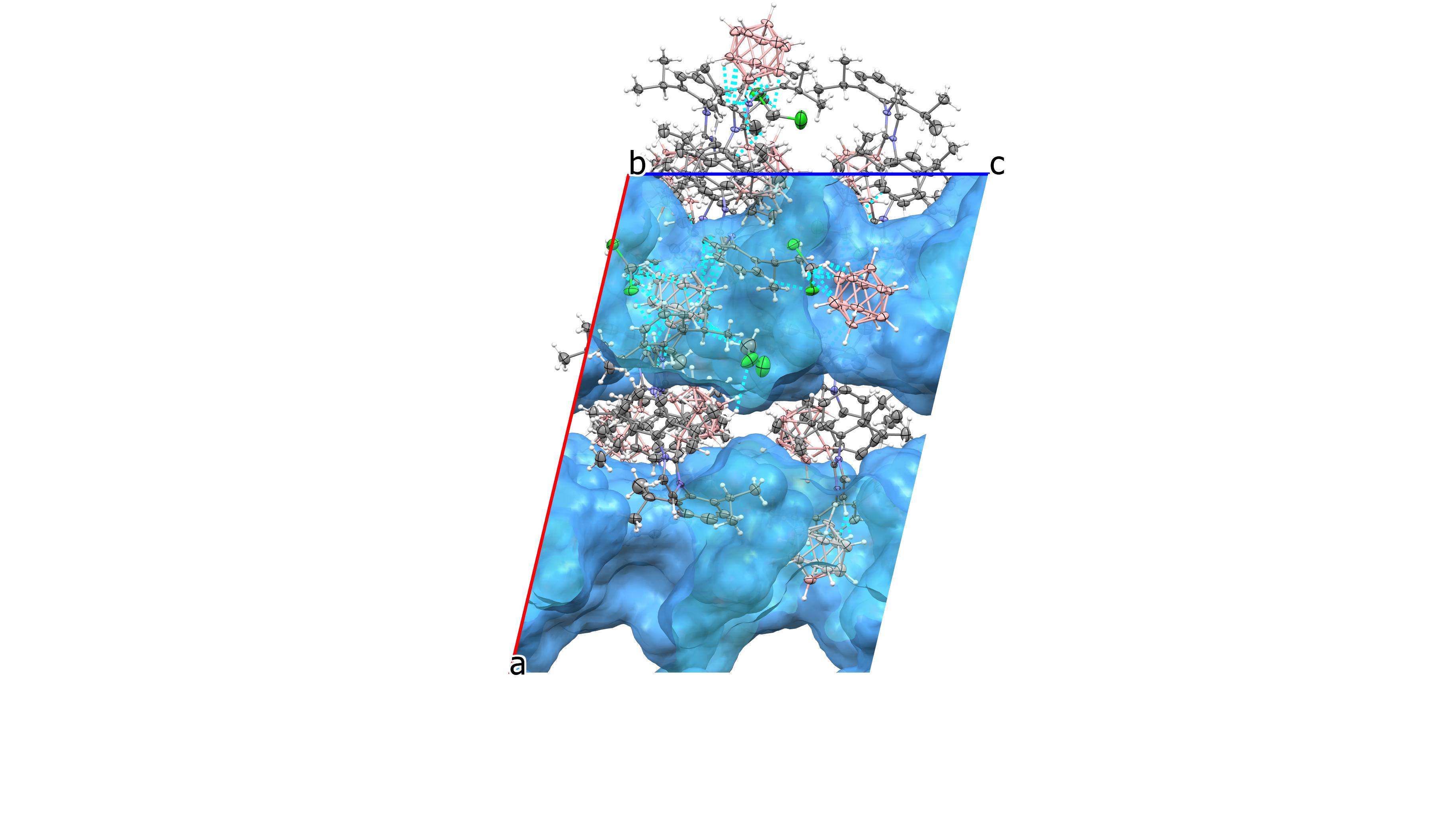

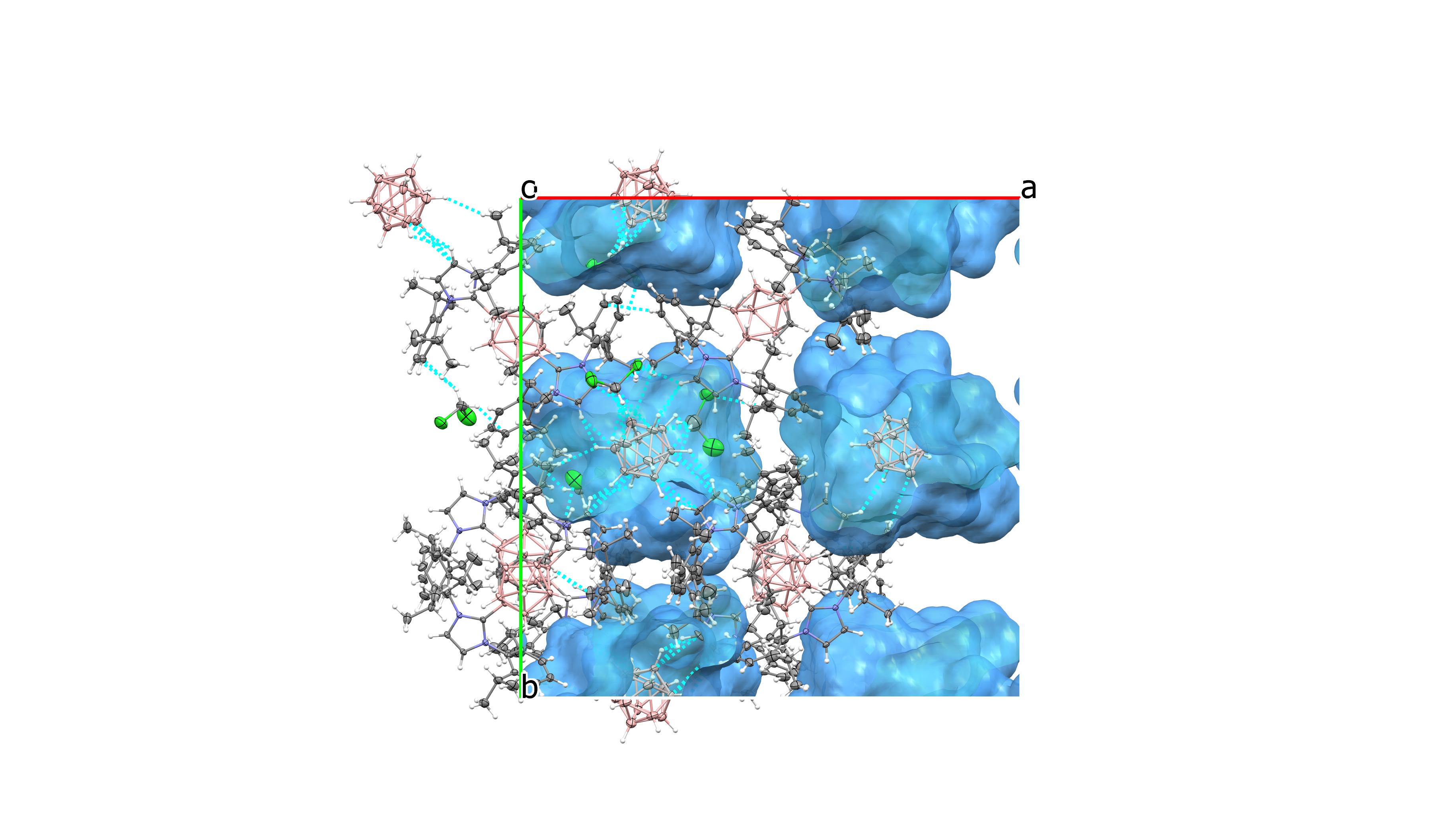


**Figure S122.** The molecular structure of ***o*-2c** (**A**). Supramolecular architecture of ***o*-2c** (**B**), voids of 25.6% of the unit cell within the structure of ***o*-2c** shown along a-, b- and c-axes, probe radius 1.2 Å. ORTEP diagrams, 40% probability level; the 2,6-di*iso*propylphenyl groups were displayed as wireframes for clarity. Water and dichloromethane molecules were omitted for clarity. Selected interatomic distances [Å]: B5-B10 1.848(8), B9-B10 2.987(9), B9-C8 1.634(7), C7-C8 1.544(7), C7-B6 1.641(7), B5-B6 1.989(8), B9-C37 1.607(7), B6-C10 1.612(7).

**Table S2.** Crystal data and structure refinement for **o-2c**.

| Crystal data | |
| --- | --- |
| Chemical formula | C_115_H_184_B_28_Cl_6_N_8_ |
| *M*_r_ | 2194.07 |
| Crystal system, space group | Monoclinic, *P*2_1_/*c* |
| Temperature (K) | 150 |
| *a*, *b*, *c* (Å) | 28.8967 (12), 28.1135 (11), 20.2878 (9) |
| β (°) | 103.379 (2) |
| *V* (Å^3^) | 16034.3 (12) |
| *Z* | 4 |
| Radiation type | Mo *K*α |
| µ (mm^−1^) | 0.15 |
| Crystal size (mm) | 0.59 × 0.55 × 0.54 |
|  | |
| Data collection | |
| Diffractometer | Bruker D8 - Venture |
| Absorption correction | – |
| No. of measured, independent and observed [*I* > 2σ(*I*)] reflections | 288891, 29099, 25411 |
| *R*_int_ | 0.046 |
| (sin θ/λ)_max_ (Å^−1^) | 0.602 |
|  | |
| Refinement | |
| *R*[*F*^2^ > 2σ(*F*^2^)], *wR*(*F*^2^), *S* | 0.158, 0.324, 1.06 |
| No. of reflections | 29099 |
| No. of parameters | 1482 |
| No. of restraints | 1470 |
| H-atom treatment | H atoms treated by a mixture of independent and constrained refinement |
|  | *w* = 1/[σ^2^(*F*_o_^2^) + (0.030*P*)^2^ + 108.*P*] where *P* = (*F*_o_^2^ + 2*F*_c_^2^)/3 |
| Δρ_max_, Δρ_min_ (e Å^−3^) | 5.98, −1.21 |

**
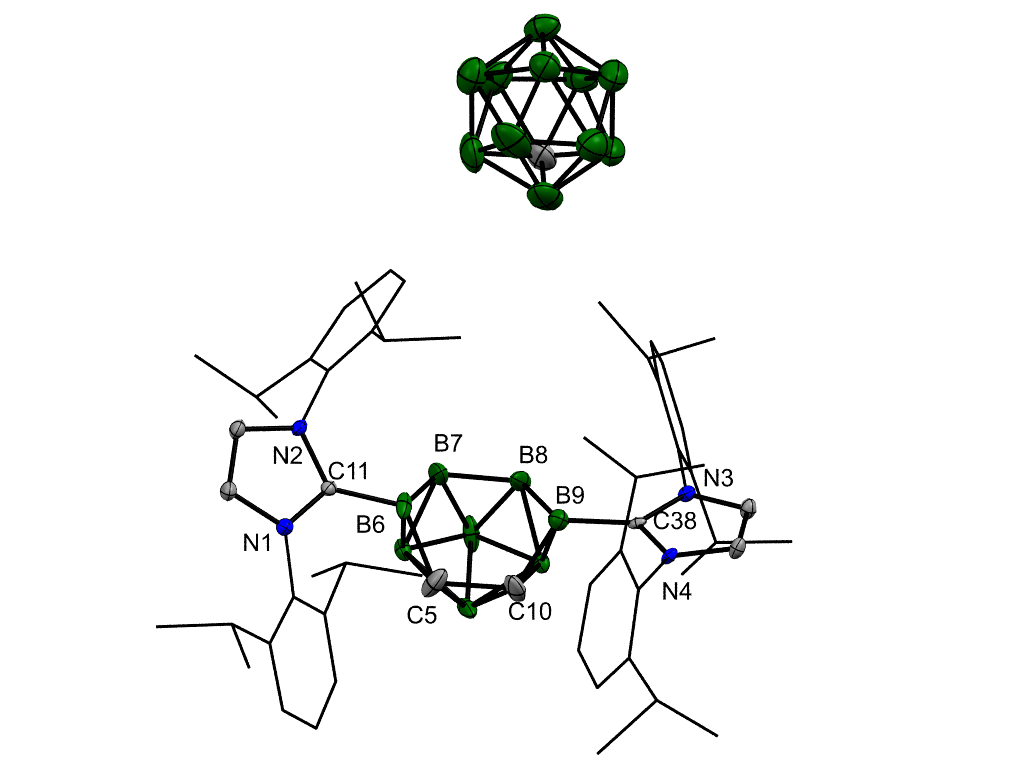
**

**Figure S123.** The molecular structure of ***o*-2d**. ORTEP diagrams, 40% probability level; the 2,6-di*iso*propylphenyl groups were displayed as wireframes for clarity. Selected interatomic distances [Å]: B5-B10 1.846(3), B9-B10 2.009(3), B9-C8 1.636(2), C7-C8 1.533(2), C7-B6 1.645(2), B5-B6 1.997(3), B9-C32 1.608(2), B6-C11 1.610(2).

**Table S3.** Crystal data and structure refinement for **o-2d**.

| Crystal data | |
| --- | --- |
| Chemical formula | 2(C_56_H_82_B_8_N_4_)·2(CH_12_B_11_)·0.5(C_6_H_14_)·2.5(CH_2_Cl_2_) |
| *M*_r_ | 2337.90 |
| Crystal system, space group | Orthorhombic, *Pbcn* |
| Temperature (K) | 150 |
| *a*, *b*, *c* (Å) | 19.9335 (9), 27.5899 (13), 27.4935 (11) |
| *V* (Å^3^) | 15120.4 (12) |
| *Z* | 4 |
| Radiation type | Mo *K*α |
| µ (mm^−1^) | 0.14 |
| Crystal size (mm) | 0.57 × 0.57 × 0.12 |
|  | |
| Data collection | |
| Diffractometer | Bruker D8 - Venture |
| Absorption correction | Multi-scan *SADABS2016*/2 - Bruker AXS area detector scaling and absorption correction |
| *T*_min_, *T*_max_ | 0.595, 0.746 |
| No. of measured, independent and observed [*I* > 2σ(*I*)] reflections | 169293, 13232, 11738 |
| *R*_int_ | 0.072 |
| (sin θ/λ)_max_ (Å^−1^) | 0.595 |
|  | |
| Refinement | |
| *R*[*F*^2^ > 2σ(*F*^2^)], *wR*(*F*^2^), *S* | 0.146, 0.333, 1.09 |
| No. of reflections | 13232 |
| No. of parameters | 741 |
| No. of restraints | 910 |
| H-atom treatment | H atoms treated by a mixture of independent and constrained refinement |
|  | *w* = 1/[σ^2^(*F*_o_^2^) + (0.1143*P*)^2^ + 58.0518*P*] where *P* = (*F*_o_^2^ + 2*F*_c_^2^)/3 |
| Δρ_max_, Δρ_min_ (e Å^−3^) | 1.24, −1.06 |


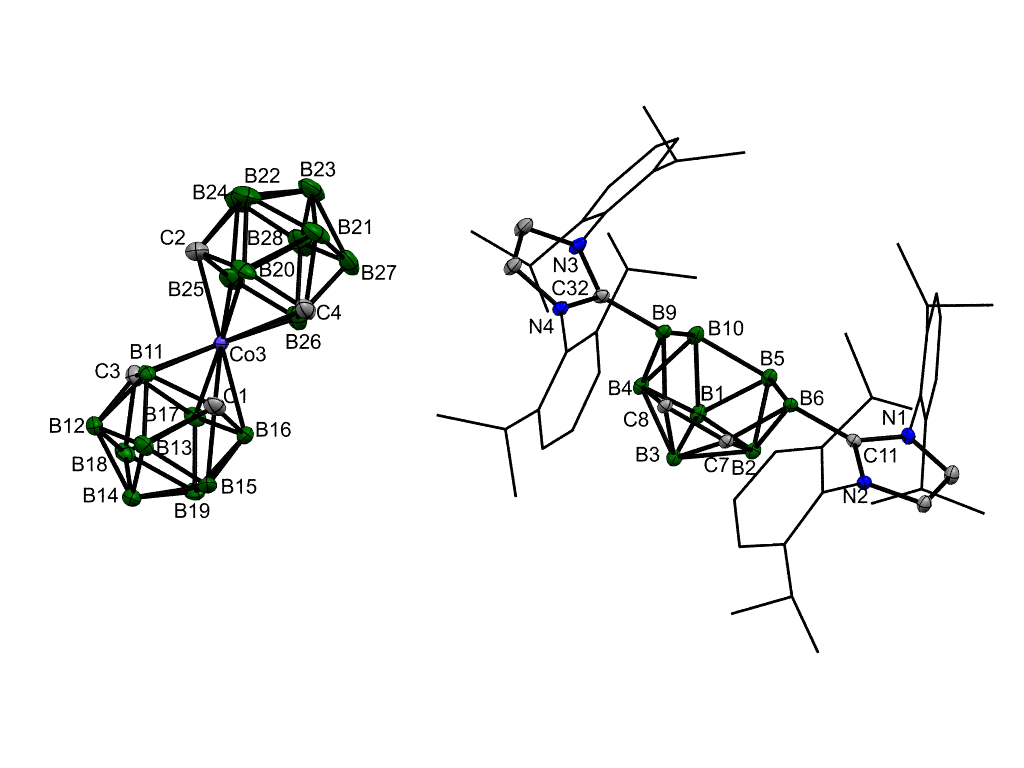


**Figure S124.** The molecular structure of ***o*-2e**. ORTEP diagrams, 40% probability level; the 2,6-di*iso*propylphenyl groups were displayed as wireframes for clarity. Selected interatomic distances [Å]: B6-B7 1.727(3), B7-B8 1.660(3), B8-B9 1.738(2), B9-C10 1.630(2), C5-C10 1.624(2), C5-B6 1.674(2).

**Table S4.** Crystal data and structure refinement for **o-2e**.

| Crystal data | |
| --- | --- |
| Chemical formula | C_60_H_104_B_26_CoN_4_·C_7_H_8_ |
| *M*_r_ | 1313.59 |
| Crystal system, space group | Triclinic, *P-*1 |
| Temperature (K) | 150 |
| *a*, *b*, *c* (Å) | 10.5201 (4), 18.5311 (7), 20.6009 (9) |
| α, β, γ (°) | 78.440 (2), 89.034 (2), 88.887 (2) |
| *V* (Å^3^) | 3933.6 (3) |
| *Z* | 2 |
| Radiation type | Mo *K*α |
| µ (mm^−1^) | 0.26 |
| Crystal size (mm) | 0.59 × 0.41 × 0.29 |
|  | |
| Data collection | |
| Diffractometer | Bruker D8 - Venture |
| Absorption correction | Multi-scan *SADABS2016*/2 - Bruker AXS area detector scaling and absorption correction Reference: Krause, L., Herbst-Irmer, R., Sheldrick G.M. & Stalke D., *J. Appl. Cryst.* 48 (2015) 3-10. |
| *T*_min_, *T*_max_ | 0.708, 0.745 |
| No. of measured, independent and observed [*I* > 2σ(*I*)] reflections | 134072, 15438, 13064 |
| *R*_int_ | 0.062 |
| (sin θ/λ)_max_ (Å^−1^) | 0.617 |
|  | |
| Refinement | |
| *R*[*F*^2^ > 2σ(*F*^2^)], *wR*(*F*^2^), *S* | 0.046, 0.143, 1.06 |
| No. of reflections | 15438 |
| No. of parameters | 869 |
| No. of restraints | 1029 |
| H-atom treatment | H atoms treated by a mixture of independent and constrained refinement |
| Δρ_max_, Δρ_min_ (e Å^−3^) | 0.76, −0.68 |

**
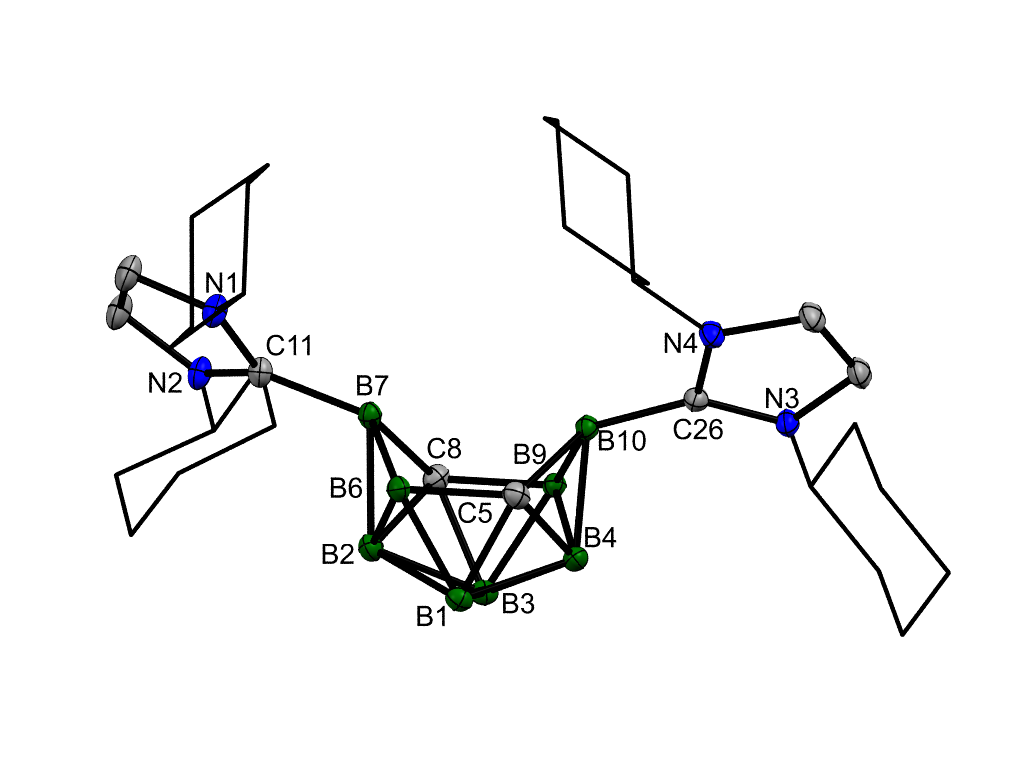
**

**Figure S125.** The molecular structure of ***p*-2^Cy^**. ORTEP diagrams, 40% probability level; the solvent (acetonitrile) molecule was omitted for clarity; the cyclohexyl groups were displayed as wireframes for clarity. Selected interatomic distances [Å]: C5-B6 1.6135(18), B6-B7 1.7966(19), B7-C8 1.7189(18), C8-B9 1.6186(18), B9-B10 1.7762(19), C5-B10 1.7261(18), B7-C11 1.6123(18), B10-C26 1.6019(17).

**Table S5.** Crystal data and structure refinement for **p-2^Cy^**.

| Crystal data | |
| --- | --- |
| Chemical formula | C_32_H_58_B_8_N_4_·C_2_H_3_N |
| *M*_r_ | 626.35 |
| Crystal system, space group | Monoclinic, *P*2_1_/*c* |
| Temperature (K) | 150 |
| *a*, *b*, *c* (Å) | 10.6784 (2), 11.9820 (2), 29.7968 (5) |
| β (°) | 98.096 (1) |
| *V* (Å^3^) | 3774.46 (11) |
| *Z* | 4 |
| Radiation type | Mo *K*α |
| µ (mm^−1^) | 0.06 |
| Crystal size (mm) | 0.17 × 0.15 × 0.06 |
|  | |
| Data collection | |
| Diffractometer | Bruker D8 - Venture |
| Absorption correction | Multi-scan *SADABS2016*/2 - Bruker AXS area detector scaling and absorption correction Reference: Krause, L., Herbst-Irmer, R., Sheldrick G.M. & Stalke D., J. Appl. Cryst. 48 (2015) 3-10. |
| *T*_min_, *T*_max_ | 0.712, 0.746 |
| No. of measured, independent and observed [*I* > 2σ(*I*)] reflections | 88034, 9332, 7807 |
| *R*_int_ | 0.049 |
| (sin θ/λ)_max_ (Å^−1^) | 0.667 |
|  | |
| Refinement | |
| *R*[*F*^2^ > 2σ(*F*^2^)], *wR*(*F*^2^), *S* | 0.054, 0.161, 1.02 |
| No. of reflections | 9332 |
| No. of parameters | 434 |
| No. of restraints | 1 |
| H-atom treatment | H atoms treated by a mixture of independent and constrained refinement |
| Δρ_max_, Δρ_min_ (e Å^−3^) | 0.61, −0.59 |


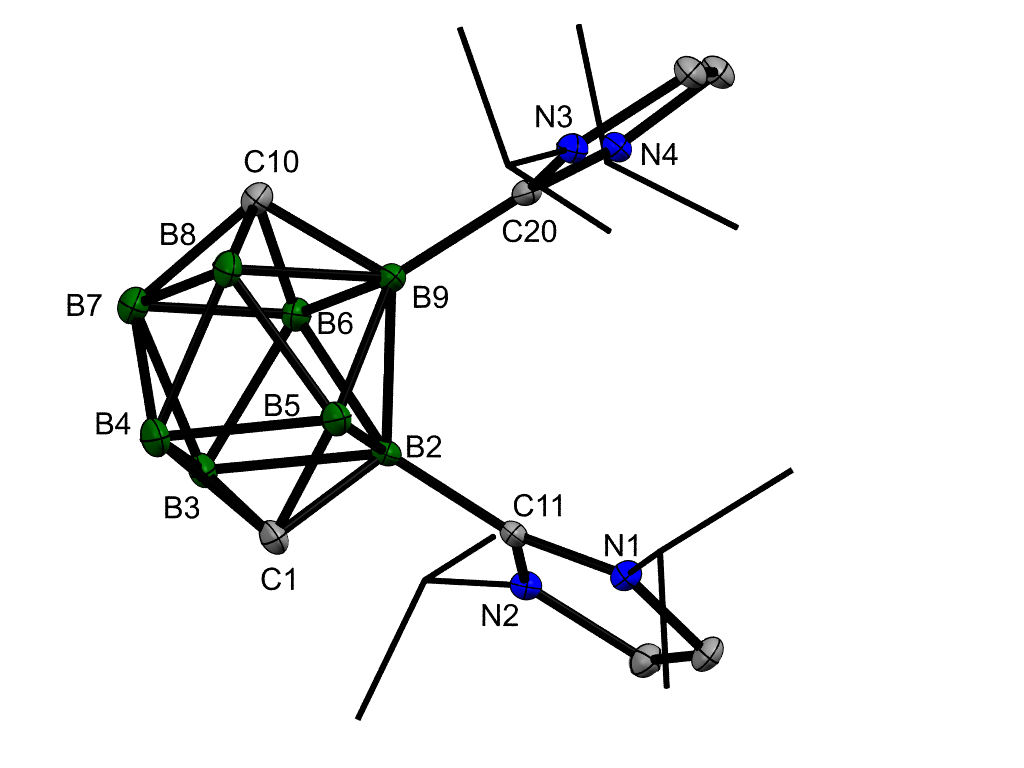


**Figure S126.** Molecular structure of the cationic part of ***p*-2a*^i^*^Pr^**. ORTEP diagram, 40% probability level; the *i*Pr groups were displayed as wireframes for clarity. The solvent molecules and anions were omitted for clarity. Selected interatomic distances [Å]: B-B 1.825(5)–1.865(5), C1-B 1.594(5)–1.611(5), C10-B 1.599(5)–1.607(4), B2-C11 1.591(4), B9-C20 1.589(4).

**Table S6.** Crystal data and structure refinement for **p-2a^iPr^**.

| Crystal data | |
| --- | --- |
| Chemical formula | C_20_H_40_B_8_N_4_·2(Cl)·0.5(C_4_H_10_O) |
| *M*_r_ | 531.00 |
| Crystal system, space group | Monoclinic, *P*2_1_/*c* |
| Temperature (K) | 150 |
| *a*, *b*, *c* (Å) | 9.774 (2), 30.076 (6), 10.373 (2) |
| β (°) | 96.48 (3) |
| *V* (Å^3^) | 3029.8 (11) |
| *Z* | 4 |
| Radiation type | Mo *K*α |
| µ (mm^−1^) | 0.24 |
| Crystal size (mm) | 0.56 × 0.19 × 0.12 |
|  | |
| Data collection | |
| Diffractometer | Bruker D8 - Venture |
| Absorption correction | Multi-scan *SADABS2016*/2 - Bruker AXS area detector scaling and absorption correction |
| *T*_min_, *T*_max_ | 0.486, 0.746 |
| No. of measured, independent and observed [*I* > 2σ(*I*)] reflections | 26957, 5209, 4875 |
| *R*_int_ | 0.044 |
| (sin θ/λ)_max_ (Å^−1^) | 0.595 |
|  | |
| Refinement | |
| *R*[*F*^2^ > 2σ(*F*^2^)], *wR*(*F*^2^), *S* | 0.070, 0.160, 1.22 |
| No. of reflections | 5209 |
| No. of parameters | 315 |
| No. of restraints | 336 |
| H-atom treatment | H-atom parameters constrained |
| Δρ_max_, Δρ_min_ (e Å^−3^) | 0.53, −0.49 |

**Table S7.** Crystal data and structure refinement for **p-2a^Cy^**.

| Crystal data | |
| --- | --- |
| Chemical formula | 2(C_32_H_56_B_8_N_4_)·3(CH_2_Cl_2_)·6(Cl)·2(H_3_O) |
| *M*_r_ | 1672.09 |
| Crystal system, space group | Monoclinic, *C*2/*c* |
| Temperature (K) | 150 |
| *a*, *b*, *c* (Å) | 11.9849 (12), 19.2069 (19), 39.299 (4) |
| β (°) | 96.433 (3) |
| *V* (Å^3^) | 8989.4 (15) |
| *Z* | 4 |
| Radiation type | Mo *K*α |
| µ (mm^−1^) | 0.41 |
| Crystal size (mm) | 0.39 × 0.19 × 0.08 |
|  | |
| Data collection | |
| Diffractometer | Bruker D8 - Venture |
| Absorption correction | *SADABS2016*/2 - Bruker AXS area detector scaling and absorption correction |
| *T*_min_, *T*_max_ | 0.570, 0.746 |
| No. of measured, independent and observed [*I* > 2σ(*I*)] reflections | 50176, 7764, 7238 |
| *R*_int_ | 0.052 |
| (sin θ/λ)_max_ (Å^−1^) | 0.595 |
|  | |
| Refinement | |
| *R*[*F*^2^ > 2σ(*F*^2^)], *wR*(*F*^2^), *S* | 0.148, 0.304, 1.30 |
| No. of reflections | 7764 |
| No. of parameters | 505 |
| No. of restraints | 552 |
| H-atom treatment | H atoms treated by a mixture of independent and constrained refinement |
|  | *w* = 1/[σ^2^(*F*_o_^2^) + (0.0001*P*)^2^ + 269.0649*P*] where *P* = (*F*_o_^2^ + 2*F*_c_^2^)/3 |
| Δρ_max_, Δρ_min_ (e Å^−3^) | 1.54, −1.12 |

**Table S8.** Crystal data and structure refinement for **p-2b^Cy^**.

| Crystal data | |
| --- | --- |
| Chemical formula | C_34_H_56_B_8_N_6_·3(CH_2_Cl_2_) |
| *M*_r_ | 890.10 |
| Crystal system, space group | Monoclinic, *P*2_1_/*c* |
| Temperature (K) | 150 |
| *a*, *b*, *c* (Å) | 9.8324 (5), 30.1833 (15), 16.5745 (9) |
| β (°) | 105.588 (2) |
| *V* (Å^3^) | 4738.0 (4) |
| *Z* | 4 |
| Radiation type | Mo *K*α |
| µ (mm^−1^) | 0.40 |
| Crystal size (mm) | 0.41 × 0.17 × 0.09 |
|  | |
| Data collection | |
| Diffractometer | Bruker D8 - Venture |
| Absorption correction | Multi-scan *SADABS2016*/2 - Bruker AXS area detector scaling and absorption correction Reference: Krause, L., Herbst-Irmer, R., Sheldrick G.M. & Stalke D., J. Appl. Cryst. 48 (2015) 3-10. |
| *T*_min_, *T*_max_ | 0.693, 0.745 |
| No. of measured, independent and observed [*I* > 2σ(*I*)] reflections | 96178, 8305, 7209 |
| *R*_int_ | 0.048 |
| (sin θ/λ)_max_ (Å^−1^) | 0.595 |
|  | |
| Refinement | |
| *R*[*F*^2^ > 2σ(*F*^2^)], *wR*(*F*^2^), *S* | 0.096, 0.264, 1.07 |
| No. of reflections | 8305 |
| No. of parameters | 541 |
| No. of restraints | 518 |
| H-atom treatment | H atoms treated by a mixture of independent and constrained refinement |
|  | *w* = 1/[σ^2^(*F*_o_^2^) + (0.1186*P*)^2^ + 17.0489*P*] where *P* = (*F*_o_^2^ + 2*F*_c_^2^)/3 |
| Δρ_max_, Δρ_min_ (e Å^−3^) | 1.11, −0.95 |

**Table S9.** Crystal data and structure refinement for **p-2d^iPr^**.

| Crystal data | |
| --- | --- |
| Chemical formula | C_34_H_60_B_8_N_8_·2(CF_3_O_3_S)·C_2_H_3_N |
| *M*_r_ | 1006.57 |
| Crystal system, space group | Triclinic, *P-*1 |
| Temperature (K) | 150 |
| *a*, *b*, *c* (Å) | 9.4593 (2), 13.9415 (3), 19.8670 (5) |
| α, β, γ (°) | 88.557 (1), 89.881 (1), 78.575 (1) |
| *V* (Å^3^) | 2567.26 (10) |
| *Z* | 2 |
| Radiation type | Mo *K*α |
| µ (mm^−1^) | 0.18 |
| Crystal size (mm) | 0.59 × 0.13 × 0.10 |
|  | |
| Data collection | |
| Diffractometer | Bruker D8 - Venture |
| Absorption correction | Multi-scan *SADABS2016*/2 - Bruker AXS area detector scaling and absorption correction Reference: Krause, L., Herbst-Irmer, R., Sheldrick G.M. & Stalke D., J. Appl. Cryst. 48 (2015) 3-10. |
| *T*_min_, *T*_max_ | 0.723, 0.746 |
| No. of measured, independent and observed [*I* > 2σ(*I*)] reflections | 123676, 12746, 11150 |
| *R*_int_ | 0.050 |
| (sin θ/λ)_max_ (Å^−1^) | 0.667 |
|  | |
| Refinement | |
| *R*[*F*^2^ > 2σ(*F*^2^)], *wR*(*F*^2^), *S* | 0.048, 0.135, 1.05 |
| No. of reflections | 12746 |
| No. of parameters | 641 |
| No. of restraints | 605 |
| H-atom treatment | H atoms treated by a mixture of independent and constrained refinement |
| Δρ_max_, Δρ_min_ (e Å^−3^) | 0.85, −0.63 |

**Reactions of *p*-2a*^i^*^Pr^ with bases**

**Figure S127.** Plausible mechanism of the reaction of ***p*-2a*^i^*^Pr^** with cyanide.

**Figure S128.** Plausible mechanism of the reaction of ***p*-2c*^i^*^Pr^** with DMAP. The triflate anions were omitted for clarity.

**Theoretical Investigations**

**Computational Methods**

All calculations were performed using the Gaussian 16 program.^[66]^ Reaction-energy profiles were computed at the B3LYP/def2-TZVP level of theory,^[67-69]^ with solvation effects accounted for via the polarizable continuum model (PCM)^[70]^ using tetrahydrofuran as the solvent. Dispersion interactions were included using the Grimme’s D3 dispersion correction.^[71]^ All optimized structures were confirmed as minima on the potential energy surface through frequency analysis at the same level of theory, with transition states exhibiting only a single imaginary frequency. Topological analysis of the electron-density function *ρ*(**r**) and charge-distribution analysis were performed using the AIMALL program package.^[72]^ For this purpose, the structures of ***p*-2a*^i^*^Pr^** and ***p*-2a^Cy^** were optimized at the B3LYP-D3(BJ)/def2-TZVP level of theory.

**The Analysis of Charge Distribution in Prepared Carboranes**

**Table S10.** QTAIM charge distribution on key atoms and the fragments of **p-2a^iPr^** and **p-2a^Cy^** [e].

| ***p*-2a^Cy^** | | | | | | | | |
| --- | --- | --- | --- | --- | --- | --- | --- | --- |
| **B5** | **B7** | **B10** | **B12** | **B14** | **B16** | **B18** | **B20** | **C2** |
| 1.019 | 1.052 | 1.075 | 1.048 | 1.153 | 1.148 | 1.113 | 1.122 | −2.096 |
| **C21** | **C23(B5)** | **C62(B20)** | **Cl101** | **Cl102** |  |  |  |  |
| −2.183 | 0.413 | 0.436 | −0.837 | −0.834 |  |  |  |  |
| Boron cage | | | Carbenes | | | Anions | | |
| 1.399 | | | 0.148, 0.121 | | | −1.671 | | |
| ***p*-2a*^i^*^Pr^** | | | | | | | | |
| **B6** | **B7** | **B10** | **B13** | **B15** | **B17** | **B19** | **B21** | **C3** |
| 1.069 | 1.068 | 1.085 | 1.079 | 1.108 | 1.133 | 1.123 | 1.109 | −2.099 |
| **C72** | **C22(B6)** | **C47(B21)** | **Cl1** | **Cl74** |  |  |  |  |
| −2.191 | 0.372 | 0.446 | −0.852 | −0.833 |  |  |  |  |
| Boron cage | | | Carbenes | | | Anions | | |
| 1.398 | | | 0.119, 0.166 | | | −1.685 | | |

**The Investigation of the Dication-Formation Mechanism**





**Figure S129.** The mechanistic pathways for the reactions of ***p*-2*^i^*^Pr^** and ***p*-2^Dipp^** with hydrogen chloride. The DFT-estimated Gibbs free-energy profiles (kcal/mol) for the reaction mechanisms of ***p*-2*^i^*^Pr^** (blue) and ***p*-2^Dipp^** (red) with hydrogen chloride. Substituents are indicated by colored dots: blue = *i*Pr, red = Dipp, and blue-red = Dipp or *i*Pr.

**Table S11.** Comparison of the DFT-estimated Gibbs free-energies from the PCM and SMD models [kcal/mol].

| Model | *p*-2*^i^*^Pr^ | TS-1 | INT-1 | TS-2 | INT-2 | TS-3 | INT-3 | TS-4 | TS-5 | *p*-2a*^i^*^Pr^ |
| --- | --- | --- | --- | --- | --- | --- | --- | --- | --- | --- |
| PCM | 0 | 4.71 | 7.39 | 24.13 | −11.24 | 17.29 | 26.58 | 37.73 | 22.97 | −35.64 |
| SMD | 0 | 6.94 | 8.98 | 25.95 | −9.02 | 23.24 | 28.52 | 35.92 | 24.74 | −36.52 |


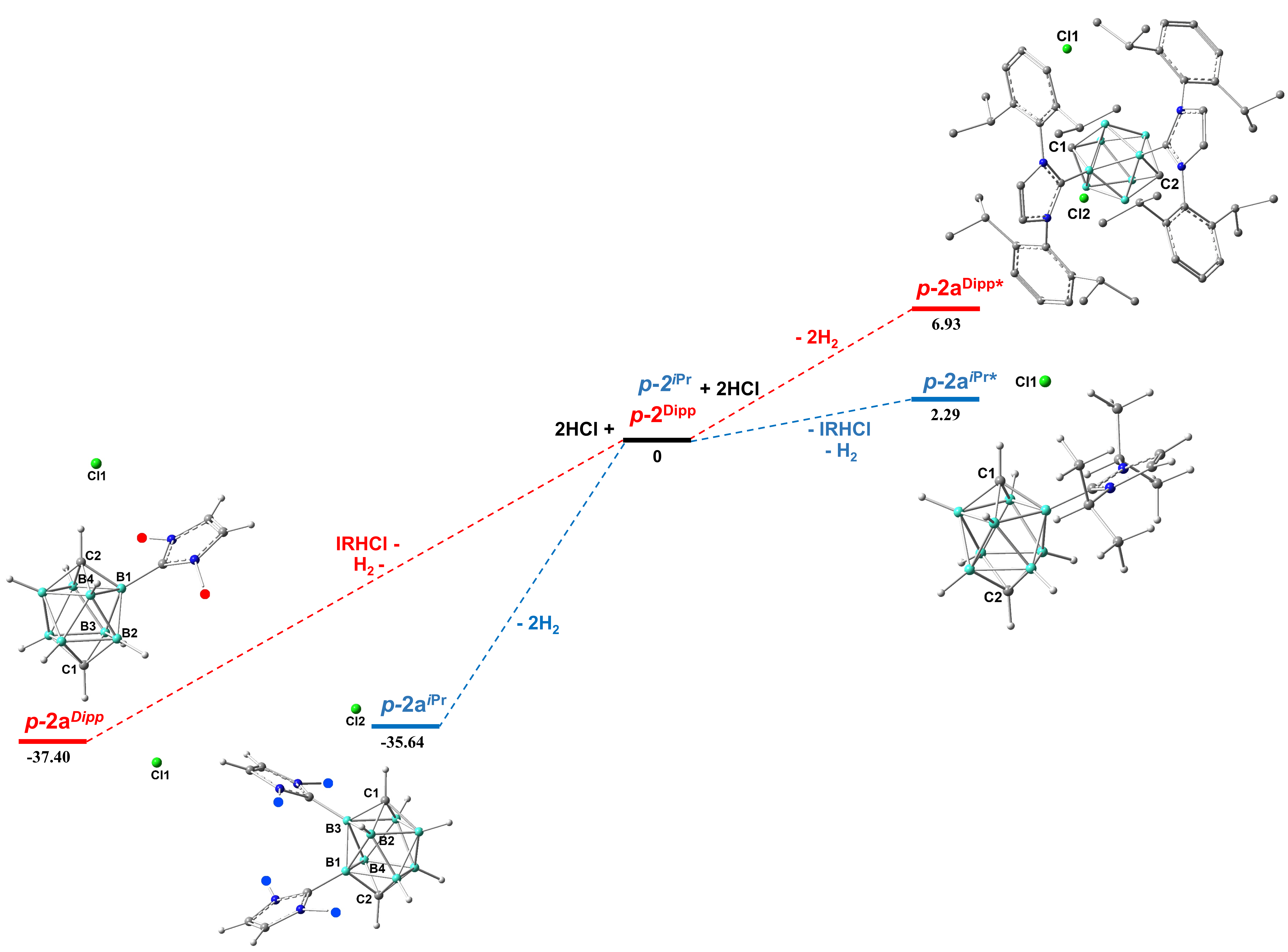


**Figure S130.** Relative Gibbs free energies (kcal/mol) for ***p-2a^Dipp^*** and ***p*-2a*^i^*^Pr^** and their alternative products ***p-2a^Dipp^**** and ***p*-2a*^i^*^Pr^*****. Hydrogen atoms in ***p*-2a^Dipp^** are omitted for clarity. IR =1,3-(2,6-*i*Pr_2_C_6_H_3_)-imidazole-2-ylidene.

**HOMO and LUMO Energy Levels**

To gain insight into the reactivity of the ten-vertex *closo*-carboranes ***p***, ***p*-*2*a^Dipp^**, ***p*-2a^iPr^** and ***p*-2a^Cy^,** we calculated their HOMO and LUMO energy levels (Table S12).

**Table S12.** Calculated HOMO and LUMO energy levels (in eV) at the B3LYP-D3(BJ)/def2-TZVP level of theory.

|  | **p** | **p-2a^Dipp^** | **p-2a^iPr^** | **p-2a^Cy^** |
| --- | --- | --- | --- | --- |
| HOMO | −8.63 | −5.03 | −4.83 | −4.88 |
| LUMO | −1.34 | −1.72 | −2.61 | −2.01 |
| Gap | 7.29 | 3.31 | 2.22 | 2.87 |

| **Compound** | **EE** | **EE+ZPE** | **EE + Thermal Energy Correction** | **EE + Thermal Enthalpy Correction** | **EE + Thermal Free Energy Correction** | **Nuclear repulsion energy** |
| --- | --- | --- | --- | --- | --- | --- |
| **p-2^iPr^** | -1205.877432 | -1205.246453 | -1205.213431 | -1205.212487 | -1205.309746 | 3227.9433175980 |
| **TS-1** | -1666.723758 | -1666.086035 | -1666.051030 | -1666.050086 | -1666.151095 | 3697.0974018716 |
| **INT-1** | -1666.724526 | -1666.080153 | -1666.044738 | -1666.043794 | -1666.146821 | 3520.1793071964 |
| **TS-2** | -1666.692214 | -1666.053354 | -1666.017975 | -1666.017031 | -1666.120136 | 3535.7171848092 |
| **INT-2** | -1665.553029 | -1664.928556 | -1664.893409 | -1664.892464 | -1664.995312 | 3588.4290548373 |
| **TS-3** | -2126.364006 | -2125.731379 | -2125.694748 | -2125.693803 | -2125.798694 | 4111.6072088506 |
| **INT-3** | -2126.348417 | -2125.712303 | -2125.674662 | -2125.673717 | -2125.783893 | 3936.0371748983 |
| **TS-4** | -2126.331351 | -2125.697138 | -2125.660281 | -2125.659337 | -2125.766124 | 3950.4385928383 |
| **TS-5** | -2125.160900 | -2124.543627 | -2124.507954 | -2124.507010 | -2124.608448 | 4133.2540002497 |
| **p-2a^iPr^** | -2125.256627 | -2124.636704 | -2124.600828 | -2124.599883 | -2124.701847 | 4208.1888299318 |
|  | | | | | | |
| **p-2^Dipp^** | -2602.446313 | -2601.154591 | -2601.086231 | -2601.085287 | -2601.256984 | 11534.8477362726 |
| **TS-1** | -3063.275574 | -3061.976678 | -3061.906520 | -3061.905576 | -3062.080591 | 12314.0875825967 |
| **INT-1** | -3063.307419 | -3062.002685 | -3061.931674 | -3061.930729 | -3062.109535 | 12130.3285791533 |
| **TS-2** | -3063.271577 | -3061.971542 | -3061.900986 | -3061.900041 | -3062.076777 | 12164.4484368099 |
| **INT-2** | -3062.111562 | -3060.824994 | -3060.754834 | -3060.753890 | -3060.928880 | 12148.2962011448 |
| **TS-3** | -3062.084096 | -3060.798509 | -3060.728799 | -3060.727855 | -3060.900208 | 12278.3083269735 |
| **p-2a^Dipp^** | -1901.549931 | -1900.836699 | -1900.797712 | -1900.796768 | -1900.904914 | 5054.7937434279 |
|  | | | | | | |
| **I^Dipp^⸱HCl** | -1621.447266 | -1620.863623 | -1620.831526 | -1620.830582 | -1620.928167 | 3310.4267587464 |
| **H_2_** | -1.179770 | -1.169708 | -1.167347 | -1.166403 | -1.181199 | 0.7110295637 |
| **HCl** | -460.837615 | -460.830965 | -460.828605 | -460.827660 | -460.848850 | 7.0035146855 |
| **p-2a^iPr^*** | -1203.272235 | -1202.889677 | -1202.868098 | -1202.867154 | -1202.938787 | 1899.0368063562 |
| **p-2a^Dipp^*** | -3521.767553 | -3520.483527 | -3520.413606 | -3520.412662 | -3520.581249 | 13169.3050460143 |

**Table S13.** Raw energy data from the PCM model (au).

**Table S14.** Raw energy data from the SMD model (au).

| **Compound** | **EE** | **EE+ZPE** | **EE + Thermal Energy Correction** | **EE + Thermal Enthalpy Correction** | **EE + Thermal Free Energy Correction** | **Nuclear repulsion energy** |
| --- | --- | --- | --- | --- | --- | --- |
| **p-2^iPr^** | -1205.8955 | -1205.2648 | -1205.2317 | -1205.2308 | -1205.3288 | 3226.1341962508 |
| **TS-1** | -1666.7409 | -1666.1035 | -1666.0685 | -1666.0676 | -1666.1689 | 3686.9202826133 |
| **INT-1** | -1666.7411 | -1666.0977 | -1666.0621 | -1666.0612 | -1666.1656 | 3518.8942150492 |
| **TS-2** | -1666.7102 | -1666.0715 | -1666.0361 | -1666.0352 | -1666.1386 | 3534.4727795440 |
| **INT-2** | -1665.569 | -1664.9455 | -1664.9111 | -1664.9101 | -1665.0108 | 3576.0117547296 |
| **TS-3** | -2126.3791 | -2125.7441 | -2125.7076 | -2125.7066 | -2125.8118 | 4086.1579406193 |
| **INT-3** | -2126.3727 | -2125.7359 | -2125.6986 | -2125.6976 | -2125.8074 | 3912.1470069512 |
| **TS-4** | -2126.3582 | -2125.7227 | -2125.6861 | -2125.6852 | -2125.7916 | 3924.8846101396 |
| **p-2a^iPr^** | -2125.2779 | -2124.659 | -2124.6229 | -2124.6219 | -2124.7265 | 4203.8011081009 |
| **HCl** | -460.83985 | -460.83326 | -460.8309 | -460.82996 | -460.85116 | 6.9805635694 |
| **H_2_** | -1.1791718 | -1.169112 | -1.166752 | -1.165808 | -1.180603 | 0.7119025645 |

**Biological Evaluations *in vitro***

***In vitro* Cytotoxicity against Human Cancer and Normal Cells**

The *in vitro* cytotoxicity of the selected compounds—***p*-2a^Cy^**, ***o*-2a**, ***p*-2a^iPr^** and Cs[Co(1,7-C_2_B_9_H_11_)_2_] (a precursor of the compound ***o*-2e**, herein referred to as ***pre*-*o*-2e**]—was evaluated alongside the standard chemotherapeutics *doxorubicin* and *cisplatin* for comparative purposes. It was assessed using the standard MTT assay after the incubation periods of 24 hours for all cell lines, and additionally 48 and 72 hours for the A2780 cell line. The tested cell lines included:

**Cancer cell lines**: ovarian carcinoma (A2780), cisplatin-resistant ovarian carcinoma (A2780R), prostate carcinoma (PC3), lung adenocarcinoma (A549), breast adenocarcinoma (MCF7), osteosarcoma (HOS), and colorectal adenocarcinoma (HT-29).

**Normal cell lines**: lung fibroblast (MRC5) and aneuploid immortal keratinocyte (HaCaT).

Furthermore, the most promising compound, ***o-2a***, was additionally tested on pancreatic-carcinoma (PANC1), colorectal-carcinoma (CaCo2) and cervical-carcinoma (HeLa) cell lines.

All cell lines were obtained from the American Type Culture Collection (ATCC) and cultured according to the supplier’s protocols. Normal human cells (MRC-5 and HaCaT) were obtained from the same commercial source (ATCC) and maintained according to the supplier’s instructions. The half-maximal inhibitory concentrations (IC_50_) were derived from dose–response curves fitted using the GraphPad Prism 6 software (GraphPad Software, San Diego, USA).

**Table S15**. **The antiproliferative activity of selected compounds and reference drugs after 24-hour incubation, assessed via the MTT assay.** IC_50_ values (μM) were determined for the tested compounds and reference drugs (*doxorubicin* and *cisplatin*) on various human cancer and normal cell lines. The values are reported as the mean ± standard deviation (SD). n.t. = not tested. The compound ***o-2a*** was additionally evaluated on PANC1, CaCo2 and HeLa cancer cell lines, yielding IC_50_ values of 3.3 ± 0.2 μM, 13.9 ± 0.4 μM and 2.7 ± 0.3 μM, respectively.

| **Compound** | **Antiproliferative activity against human cancer and normal cell lines (IC_50_ ± SD)** | | | | | | | | |
| --- | --- | --- | --- | --- | --- | --- | --- | --- | --- |
|  | **A2780** | **A2780R** | **PC3** | **A549** | **MCF7** | **HOS** | **HT-29** | **MRC5** | **HaCaT** |
| ***p*-2a^Cy^** | 11.6±1.4 | 11.6±1.3 | >25 | >25 | >25 | >25 | >25 | >25 | >25 |
| ***o*-2a** | 1.4±0.3 | 1.3±0.4 | 2.4±0.1 | 5.0±3.7 | 3.2±0.1 | 2.5±0.1 | 3.4±0.5 | 2.7±0.4 | 2.8±0.5 |
| ***p*-2a*^i^*^Pr^** | >5 | >5 | >5 | >5 | >5 | >5 | >5 | >5 | >5 |
| **pre-*o*-2e** | >50 | >50 | >50 | >50 | >50 | >50 | >50 | >50 | >50 |
| *Doxorubicin* | 2.1±1.1 | 3.6±0.2 | >20 | >20 | 3.7±3.4 | 9.1±3.9. | >20 | >20 | n.t. |
| *Cisplatin* | 17.2±0.9 | >50 | >50 | >50 | 33.0±4.1 | 33.2±4.4 | >50 | >50 | >50 |

***Note*:** A2780 – ovarian carcinoma, A2780R – cisplatin-resistant ovarian carcinoma, PC3 – prostate carcinoma, A549 – lung adenocarcinoma, MCF7 – breast adenocarcinoma, HOS – osteosarcoma, HT-29 – colorectal adenocarcinoma; MRC5 – lung fibroblast, HaCaT – aneuploid immortal keratinocyte

**Table S16. The effect of incubation time on the antiproliferative activity of selected compounds.** IC_50_ values (μM) were determined for the tested complexes and reference drugs (*doxorubicin*, *cisplatin*) on the A2780 human ovarian carcinoma cell line (A2780) after incubation at varying time intervals. The values are reported as the mean ± standard deviation (SD). The IC_50_ values obtained on A2780 after 24 hours of incubation slightly differ from those given in Table 1 due to the use of cells from a different passage.

| **Compound** | **IC_50_ ± SD in µM** | | |
| --- | --- | --- | --- |
|  | **24h** | **48h** | **72h** |
| ***p*-2a^Cy^** | 12.3 ± 1.5 | 12.9 ± 1.1 | 16.4 ± 1.0 |
| ***o*-2a** | 1.6 ± 0.3 | 0.5 ± 0.1 | 0.354 ± 0.004 |
| ***p*-2a*^i^*^Pr^** | >5 | >5 | >5 |
| ***pre-o-*2e** | >50 | >50 | >50 |
| *Doxorubicin* | 2.9 ± 0.8 | 0.2 ± 0.1 | 0.2 ± 0.05 |
| *Cisplatin* | 15.2 ± 0.3 | 4.0 ± 1.7 | 3.2 ± 0.5 |

**Cell-Cycle Analysis**

The A2780 cells (Sigma, 93112519-1VL) were cultured according to the manufacturer's instructions and seeded at a density of 1×10^4^ cells per well in 96-well cell-culture plates. After 24 hours of incubation, the cells were treated with solutions containing half-maximal cytotoxic concentrations (IC₅₀) of the tested compounds and were further incubated at 37 °C for an additional 24 hours. Following treatment, the cells were washed once with PBS (0.1 M, pH 7.4), and cell-cycle analysis was performed using the BD Cycletest^TM^ Plus DNA kit (Becton Dickinson, USA) in accordance with the manufacturer’s protocol. The data were acquired using a BD FACSVerse flow cytometer (Becton Dickinson, USA) in three independent experiments, each performed in duplicate. For each sample, a minimum of 5×10^3^ events was recorded.

**The Induction of Cell Death and the Related Processes**

To study the induction of A2780-cell death by the selected compounds, two complementary methods were employed. Apoptosis was assessed using the Annexin V-FITC/PI commercial kit (V13242, Thermo Fisher Scientific, USA). Afterwards, caspase-3/7 activation was evaluated using the CellEvent™ Caspase-3/7 Green Flow Cytometry Assay Kit (C10427, Thermo Fisher Scientific, USA). Both assays were conducted in accordance with the manufacturers’ protocols, with one modification in the caspase assay: the CellEvent^TM^ Caspase-3/7 Green Detection Reagent was used exclusively for the detection of caspase-3/7 activation. The general procedure was as follows: A2780 cells were seeded at a density of 5×10^4^ cells per well in a 24-well cell-culture plate and cultured for 24 hours. Subsequently, the cells were treated with solutions containing half-maximal cytotoxic concentrations (IC₅₀) of the tested compounds and incubated for an additional 24 hours. Following treatment, the cells were washed once with PBS (0.1 M, pH 7.4) and trypsinized using 0.25% trypsin-EDTA (Gibco™). Trypsin activity was neutralized by adding the cell-culture medium to a final volume of 500 µl. The resulting cell suspensions were divided into two 250-µl aliquots: one for the Annexin V-FITC/PI assay and the other for the caspase-3/7 activation assay. Both assays were performed using a BD FACSVerse flow cytometer (Becton Dickinson, USA) in three independent experiments, each conducted in duplicate.

**Mitochondrial-Membrane Potential Analysis**

The A2780 cells were seeded in 24-well cell-culture plates at a density of 5×10^4^ cells per well and incubated at 37 °C for 24 h. The next day, the cells were treated with solutions containing half-maximal cytotoxic concentrations (IC₅₀) of the tested compounds and further incubated for an additional 24 hours. After the treatment, the cells were washed with PBS, detached using a 0.25% trypsin-EDTA (Gibco™) solution, and resuspended in cell-culture medium. The cells were then concentrated and isolated by centrifugation and resuspended in a staining solution prepared according to the manufacturer’s protocol using the MITO-ID^®^ Membrane Potential Detection Kit (Enzo Life Sciences, USA). Finally, the stained samples were analyzed using a BD FACSVerse flow cytometer (Becton Dickinson, USA). As a positive control, the cells were treated with a solution of 50 µM carbonyl cyanide 3-chlorophenylhydrazone (CCCP). Three independent experiments were conducted, each in duplicate.

**The Induction of Autophagy**

The induction of autophagy was assessed using the CYTO-ID^®^ Autophagy Detection Kit 2.0 (ENZO, USA) in accordance with the manufacturer’s protocol. Briefly, A2780 cells were seeded in a 24-well cell-culture plate at a density of 5×10^4^ cells per well and incubated at 37 °C for 24 hours. The next day, the cells were treated with solutions containing half-maximal cytotoxic concentrations (IC₅₀) of the tested compounds and further incubated for an additional 24 hours. After the treatment, the cells were washed once with PBS (0.1 M, pH 7.4), treated with a 0.25% trypsin-EDTA (Gibco™) solution, and resuspended in cell-culture medium. The cells were then stained with CYTO-ID^®^ Green stain solution and incubated for 30 minutes in the dark. After a final PBS wash, the cell suspensions were analyzed using a BD FACSVerse flow cytometer (Becton Dickinson, USA) in three separate experiments, with a minimum of 10^4^ events recorded for each sample prepared in duplicates. As a positive control, the cells were incubated with a mixture of 10 µM chloroquine (CQ) and 0.5 µM rapamycin (Rap) for 24 hours.


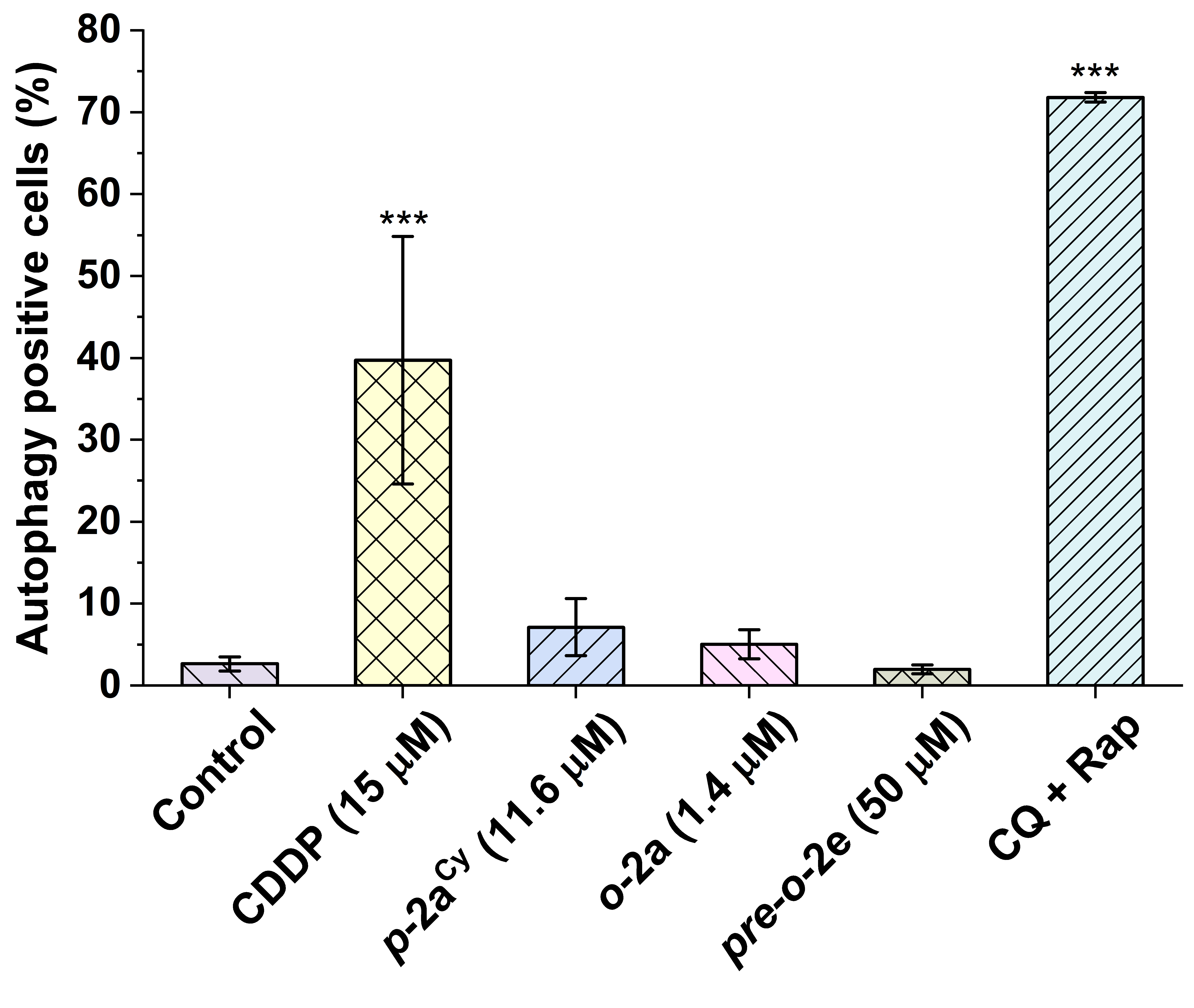


**Figure S131.** **The effect of the selected compounds on the induction of autophagy.** A2780 cells were incubated with half-maximal cytotoxic concentrations (based on MTT-assay results) of the tested compounds for 24 hours. Significance levels: * p<0.05, ** p<0.01, *** p<0.005. The positive control consisted of a mixture of well-known autophagy activators—chloroquine (10 µM) and rapamycin (0.5 µM)—applied to A2780 cells for 24 hours.


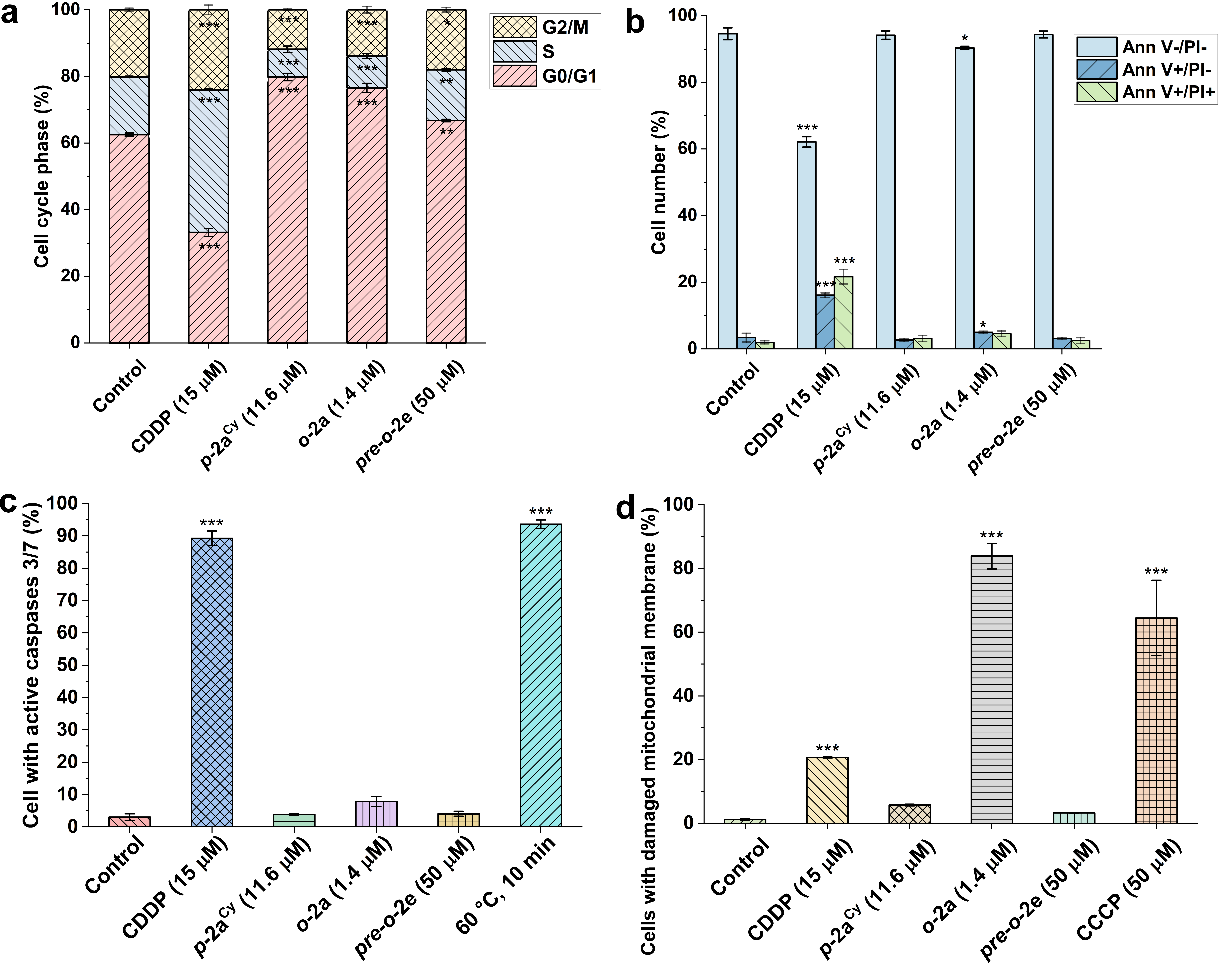


**Figure S132.** **Cellular effects of the tested compounds in A2780 cells.** (**a**) The effects of the tested compounds on the cell cycle. (**b**) The induction of apoptosis/necrosis in the Annexin V/PI assay. (**c**) The activation of executioner caspases Casp 3/7. (**d**) The effect on the mitochondrial-membrane potential in A2780 cells after 24-hour incubation using half-maximal cytotoxic concentrations (based on MTT-test assay results). Significance levels: *p<0.05, **p<0.01, ***p<0.005.

**The Induction of Intracellular ROS/Superoxide Production**

The intracellular levels of reactive oxygen species (ROS) and superoxide were assessed in A2780 cells after incubation with solutions containing the half-maximal cytotoxic concentrations (IC₅₀) of the tested compounds. The analysis was performed using the ROS-ID^®^ Total ROS/Superoxide detection kit (Enzo Life Sciences, US) in accordance with the manufacturer’s protocol. The cells were seeded in 96-well cell-culture plates at a density of 10^4^ cells per well and cultured for 24 hours. Subsequently, they were incubated with the tested compounds for an additional 24 hours. After the treatment, the cells were stained with a ROS/superoxide probe mixture and incubated in the dark at 37 °C for 60 minutes. Finally, the fluorescence in the individual samples was measured using an Infinite M200Pro microplate reader (Tecan, Switzerland). Vehicle-treated cells served as controls. To evaluate oxidative-stress modulation, the fluorescence of the cells was measured either in the absence or presence of 500 µM pyocyanin, a known oxidative-stress inducer. Three independent experiments were conducted, each in triplicate.


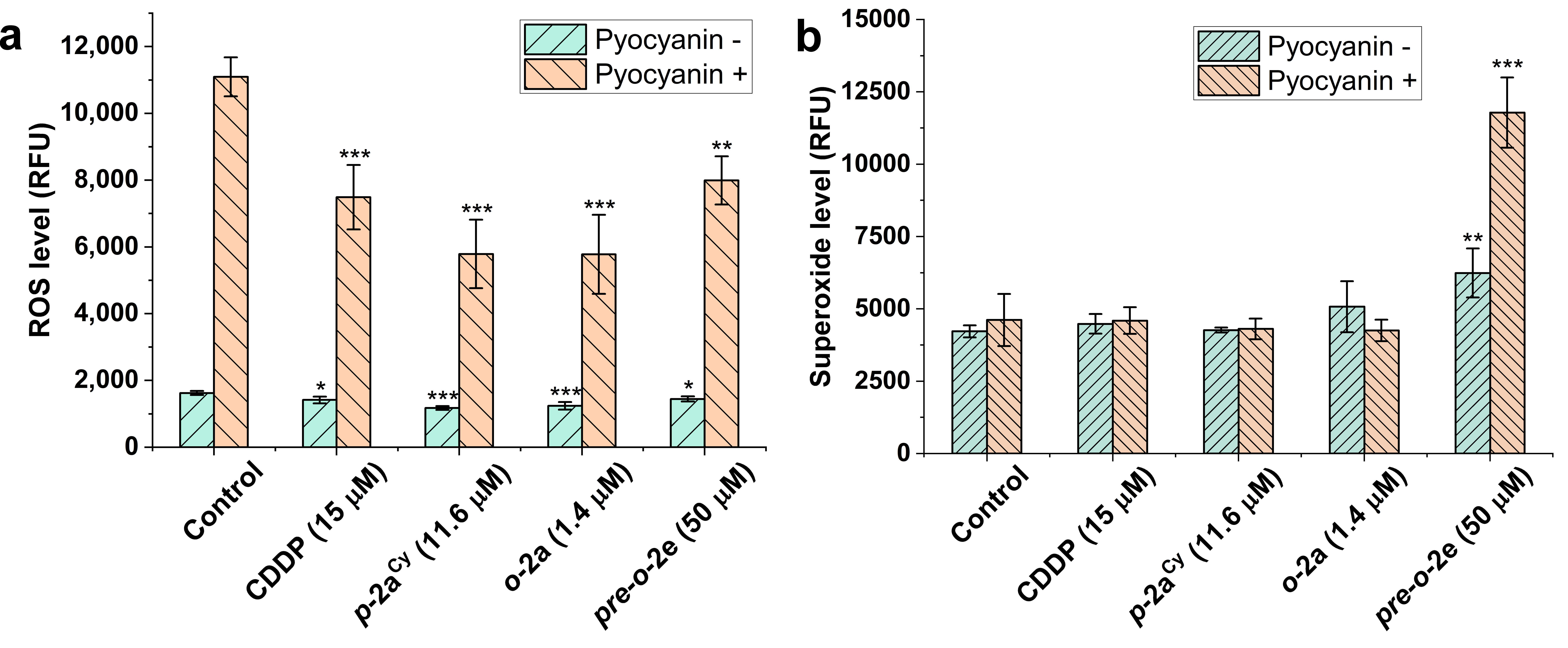


**Figure S133**. **Pro- and anti-oxidant effects of the tested compounds.** (**a**) The effects of the tested compounds on the overall ROS levels. (**b**) The effects of the tested compounds on superoxide levels. A2780 cells were incubated for 24 hours with the half-maximal cytotoxic concentrations of the tested compounds, either in the absence of the oxidative-stress inducer pyocyanin (Pyocyanin−) or in its presence (Pyocyanin+). Significance levels: * p<0.05, ** p<0.01, *** p<0.005.

**References:**

[59] O. L. Tok, M. Bakardjiev, B. Štíbr, D. Hnyk, J. Holub, Z. Padělková, A. Růžička, *Inorg. Chem.* **2016**, *55*, 8839−8843.

[60] J. Holub, T. Jelínek, Z. Janoušek, *Collect. Czech. Chem. Commun.* **2002**, *67*, 949−952.

[61] M. F. Hawthorne, D. Young, T. Andrews, D. V. Howe, R. Piling, A. Pitts, M. Reintjes, L. Warren, P. Wegner, *J. Am. Chem. Soc.* **1968**, *90*, 879–896.

[62] S. L. Holt, *Inorg. Synth.* **1983**, *22*, 227 (1983).

[63] T. Schaub, U. Radius, *Chem. Eur. J.* **2005**, *11*, 5024–5030.

[64] M. Tobisu, A. Yasutome, H. Kinuta, K. Nakamura, N. Chatani, *Org. Lett.* **2014**, *16*, 5572–5575.

[65] G. M. Sheldrick, *Acta Cryst.* **2015**, *A71*, 3–8.

[66] M. J. Frisch, G. W. Trucks, H. B. Schlegel, G. E. Scuseria, M. A. Robb, J. R. Cheeseman, G. Scalmani, V. Barone, G. A. Petersson, H. Nakatsuji, X. Li, M. Caricato, A. V. Marenich, J. Bloino, B. G. Janesko, R. Gomperts, B. Mennucci, H. P. Hratchian, J. V. Ortiz, A. F. Izmaylov, J. L. Sonnenberg, D. Williams-Young, F. Ding, F. Lipparini, F. Egidi, J. Goings, B. Peng, A. Petrone, T. Henderson, D. Ranasinghe, V. G. Zakrzewski, J. Gao, N. Rega, G. Zheng, W. Liang, M. Hada, M. Ehara, K. Toyota, R. Fukuda, J. Hasegawa, M. Ishida, T. Nakajima, Y. Honda, O. Kitao, H. Nakai, T. Vreven, K. Throssell, J. A. Montgomery, Jr., J. E. Peralta, F. Ogliaro, M. J. Bearpark, J. J. Heyd, E. N. Brothers, K. N. Kudin, V. N. Staroverov, T. A. Keith, R. Kobayashi, J. Normand, K. Raghavachari, A. P. Rendell, J. C. Burant, S. S. Iyengar, J. Tomasi, M. Cossi, J. M. Millam, M. Klene, C. Adamo, R. Cammi, J. W. Ochterski, R. L. Martin, K. Morokuma, O. Farkas, J. B. Foresman, D. J. Fox, Gaussian 16, Wallingford, CT, **2016**.

[67] A. D. Becke, *J. Chem. Phys.* **1993**, *98*, 5648–5652.

[68] C. Adamo, V. Barone, J. Chem. Phys. **1999**, *110*, 6158–6170.

[69] F. Weigend, R. Ahlrichs, *PCCP* **2005**, *7*, 3297–3305.

[70] J. Tomasi, B. Mennucci, R. Cammi, *Chem. Rev.* **1998**, *105*, 2999-3094.

[71] S. Grimme, J. Antony, S. Ehrlich, H. Krieg, *J. Chem. Phys.* **2010**, *132*, 154104.

[72] T. A. K. AIMAll (Version 19.10.12), TK Gristmill Software (aim.tkgristmill.com), Overland Park KS, USA, **2019**.
